# Primer-walking sequencing identifies predominantly noncoding TP53 variants in canine cancers

**DOI:** 10.4142/jvs.25325

**Published:** 2026-05-27

**Authors:** Ramya Mathiyalagan, Chanjoong Lee, Yongsuk Jo, Jaebeom Lee, Joong-Hyun Song

**Affiliations:** 1 Department of Chemistry, Chungnam National University, Daejeon 34134, Korea.; 2 Department of Veterinary Internal Medicine, College of Veterinary Medicine, Chungnam National University, Daejeon 34134, Korea.

**Keywords:** Comparative oncology, sanger sequencing, tumor genetics, untranslated region, somatic variation

## Abstract

**Importance:**

Tumor protein p53 (TP53) alterations are frequently reported in canine cancers but remain incompletely characterized due to differences in reference genomes and sequencing cost.

**Objective:**

To evaluate a cost-efficient primer-walking approach for TP53 variant screening in dogs with spontaneous tumors.

**Methods:**

Tumor tissue (n = 10) or blood (n = 16) was collected from 26 dogs with suspected neoplasia. Interpretable TP53 sequences spanning introns and exons 2–11, excluding long intron 1 and non-protein-coding exon 1, were obtained by primer-walking polymerase chain reaction and Sanger sequencing. Bidirectional reads were compared with the Ensembl ROS_Cfam_1.0 v114 reference sequence.

**Results:**

TP53 gene sequence variations were observed in 18/26 sequenced tumor cases, including one exonic frameshift insertion and 17 noncoding variants: 13 intronic variants and 4 variants in the 5′ untranslated region. Recurrent substitutions and deletions occurred in the intron 8–10 region. Variants were detected in lymphomas and carcinomas, including cases from several small breeds, but no significant association with tumor type or breed was demonstrated.

**Conclusions and Relevance:**

Primer-walking Sanger sequencing can screen TP53 regions beyond coding exons in canine tumor samples. Matched tumor-normal sequencing is needed to determine somatic versus germline origin and to evaluate noncoding TP53 variants as potential biomarkers or drivers.

## INTRODUCTION

Canine cancers parallels to humans. The tumor suppressor gene (tumor protein p53 [TP53]) regulates DNA repair, apoptosis, and cell cycle [1]. Its dysfunction drives tumorigenesis and poor clinical outcomes in both canine and human cancers, emphasizing its potential as a biomarker for prognosis and targeted therapies [2,3,4].

Despite significant gaps remaining due to uncharacterized TP53 mutations (potentially oncogenic variants) [5], most studies rely on the outdated CanFam3.1 assembly. Relatively few canine cancer screenings [6,7,8] have used a higher-quality canFam4 reference genome [9].

Challenges in detecting and characterizing TP53 oncogenic variants in canine cancers include breed-specific sequence variations, several incomplete annotations in public databases, and limited standardized methods. Sanger sequencing, next-generation sequencing [4], and polymerase chain reaction (PCR) methods have been employed with varying success [10]. Accurate identification of mutations across the entire TP53 gene, including the coding, non-coding and regulatory regions, remains technically demanding, especially in clinical samples with variable DNA quality and tumor heterogeneity. Thus, this study aimed to answer the question of the optimal canine TP53 gene reference sequence selection and its application in the screening of clinical cancer samples.

## METHODS

### Canine TP53 reference gene sequence analysis and primer walking

Three important canine TP53 gene reference sequences (ROS_Cfam_1.0_NC_051809.1:32665395-32678721, Dog10K_Boxer_Tasha_NC_006587.4:32699559-32713071, and UU_Cfam_GSD_1.0_NC_049226.1:32768467-32781775) from NCBI [11] (Annotation Release_106) of complete 13.3 kb genomic sequence forward strands (Supplementary Data 1) (initially accessed in Jan 2024 and re-accessed in Aug 2025 for figure preparation) were aligned to compare using Clustal Omega [12] (Aug 2025). Furthermore, NCBI_ROS_Cfam_1.0_NC_051809.1:32665395-32678721 was aligned with Ensembl_ROS_Cfam_1.0 (same v111_v114) [13] (Supplementary Data 2) TP53 gene sequences using Clustal Omega [12] (re-accessed in July 2025 for figure preparation). The Ensembl_ROS_Cfam_1.0_forward sequence was used for TP53 primers (Supplementary Table 1).

### Clinical cancer samples collection

Samples (n = 26) (tissue [n = 10] or blood [n = 16]) were collected from privately owned dogs diagnosed with spontaneous neoplasia at the Veterinary Medical Teaching Hospital, Chungnam National University, after obtaining informed owner consent and ethics approval. Tumor tissue was obtained by diagnostic biopsy when available; when only peripheral samples could be collected, whole blood in ethylenediaminetetraacetic acid was used as an alternative DNA source (Supplementary Table 2). Paired blood or non-diseased control samples were not included in this study. Samples were stored at −80°C, and histopathological diagnoses were performed by IDEXX Laboratories and Konkuk University’s Small Animal Tumor Diagnostic Center.

### Genomic DNA extraction and PCR

Genomic DNA was extracted using the Total DNA Mini Kit (Axen, Cat No: MG-P-005; Medicogen, Korea) following the manufacturer's protocol. DNA yield and purity were measured spectrophotometrically, and 20–100 ng of DNA was used for PCR. PCR was performed in 20 µL reactions containing 10 µL of 2× H_Taq_PCR Master Mix (Axen, Cat No: MBAE-110; Medicogen), 1 µL of each primer (10 pmol), 2–5 µL of genomic DNA, and nuclease-free water. Cycling conditions: initial denaturation at 95°C_5 min; 35 cycles of 95°C_30 sec, 60°C_30 sec, 72°C_1 min and final extension at 72°C_10 min in DNAEngineTetrad2-Thermal Cycler (Bio-Rad, USA).

### Sanger sequencing and TP53 gene variant analysis

Amplified PCR products were purified (ExoSAP-IT Express PCR Product Cleanup Reagent; ThermoFisher Scientific, USA), sequenced (BigDye_Terminator_v3.1_Kit; Applied Biosystems, USA), and analyzed (ABI PRISM3730xl_DNA_Analyzer; Applied Biosystems). Sequences were aligned to the reference genome ROS_Cfam_1.0_forward_4.6kb_(ENSCAFT00845043577.1) (Supplementary Data 3) using DNASTAR SeqMan_Ultra, BioEdit (7.2.5; 12/11/2013) [14] and Genious Prime and variants were designated according to the HGVS guidelines relative to the reference. These variants were observed in tumor samples; however, germline variants in the corresponding normal tissues were not determined. Given the descriptive nature of this study and the absence of predefined comparison groups, no statistical analyses were performed.

## RESULTS

### Canine TP53 reference gene sequence analysis

**Fig. 1:**
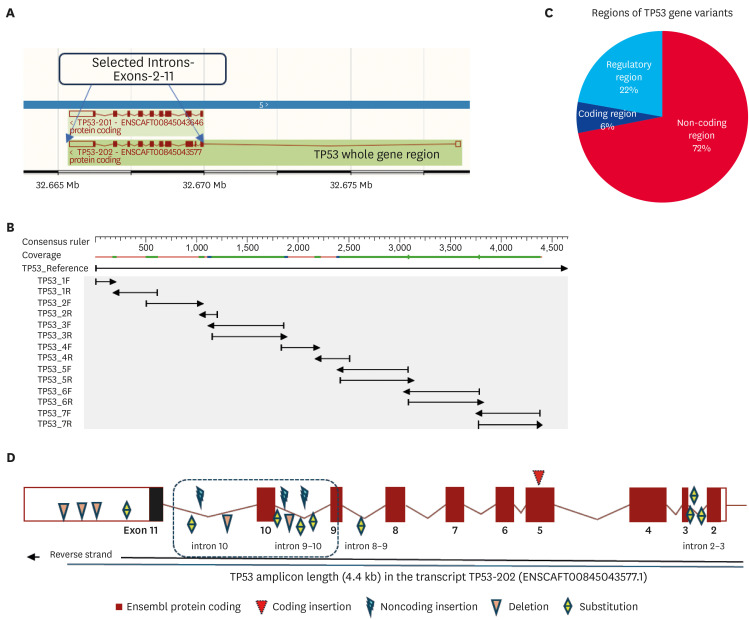
TP53 target region, primer-walking strategy, and distribution of detected variants. (A) TP53 genomic locus showing the amplified target region spanning introns and exons 2–11; intron 1 and exon 1 were excluded. (B) Primer-walking polymerase chain reaction strategy using seven primer pairs to cover the approximately 4.4-kb TP53 target region, with additional flanking sequence used for primer design. (C) Distribution of detected variants by genomic region: coding, non-coding, and regulatory regions. (D) Schematic map of the 18 distinct variants detected across the TP53 target region in this cohort. TP53, tumor protein p53; bp, base pairs.

Alignment of the three primary canine TP53 reference sequences revealed variations in gene length and structural composition (Supplementary Fig. 1A). The ROS_Cfam_1.0 assembly was selected for subsequent variant screening, as it exhibited fewer insertions and deletions than the Dog10K and GSD references. Ensembl_ROS_Cfam_1.0_(ENSCAFT00845043577.1_chr5) was further used as small deletions were observed in NCBI_ROS_Cfam_1.0 (Supplementary Fig. 1B). Consequently, primers were designed to encompass introns_exons_2–11 (excluding the extensive intron_1 and non-protein-coding exon_1) (Fig. 1A), using a primer walking strategy (Fig. 1B).

### Clinical samples overview

The study cohort comprised dogs aged 6–16 years, representing multiple breeds (Golden Retriever, Poodle, Maltese, Shih Tzu, Beagle, Belgian Malinois, Jindo, Labrador Retriever, and Pomeranian). The major tumor types were as follows: mast cell tumors (MCTs) (6/26), lymphoma (5/26), carcinoma (7/26), and sarcoma (6/26) (Table 1). Details are provided in Supplementary Table 2. The histopathological results are shown in Supplementary Data 4. The Maltese breed had a comparatively higher number of cases of MCT, squamous cell carcinoma (SCC), osteosarcoma (OSA), pulmonary carcinoma, and renal cell carcinoma cases in our cohort.

**Table 1:** Cohort characteristics and sequenced materials

Tumor type	Breeds	Age	Sex (M/F)	Sequenced material
Adenoma (1)	Doberman Pinscher	8 (8)	0 M/1 F	Tissue
Adenocarcinoma (1)	Jindo dog	11 (11)	0 M/1 F	Tissue
Carcinoma (7)	Jindo dog, Poodle, Shih Tzu, Maltese	12 (8–16)	4 M/3 F	Tissue (2), blood (5)
Lymphoma (5)^a^	Shih Tzu, Pomeranian, Poodle, Cocker Spaniel	8 (6–14)	3 M/2 F	Blood
MCT (6)^b^	Belgian Malinois, Jindo dog, Maltese, Welshi Corgi, Labrador Retriever	9 (8–14)	4 M/2 F	Tissue (3), blood (3)
Melanoma (1)	Medium Poodle	10 (10)	1 M/0 F	Blood
Myxoid neoplasm (1)	Jindo dog	10 (10)	0 M/1 F	Tissue
Sarcoma (6)^a,b^	Beagle, Poodle, Golden Retriever, Maltese, Labrador Retriever	10 (6–14)	3 M/3 F	Tissue (2), blood (4)

Values are presented as median (interquartile range) or number.

For sex (M/F), neutered/spayed animals were counted as M/F, respectively.

The age range was calculated from the minimum to maximum age for each tumor type.

Detailed per-case data are provided in Supplementary Table 2.

M, male; F, female; MCT, mast cell tumor.

^a^One sample was present in both lymphoma and sarcoma.

^b^Indicates one sample was present in both the MCT and sarcoma groups.

### TP53 gene sequence variations

Totally 18/26 sequenced samples in our cohort showed variations in the TP53 gene sequences. The non-amplified (~200 bp) and remaining samples were excluded because of PCR sequencing failure, including multiple bands, low–DNA concentration/poor sequencing chromatogram quality, and non-tumor samples.

Targeted sequencing identified 18 types of TP53 variants, including one exonic frameshift insertion and 17 (intronic and untranslated region [UTR]) variants (Table 2, Fig. 1D, Supplementary Data 3). The coding variant (Ins_G_3401_Exon 5; c.408; p.136ins) was predicted to have a frameshift within the DNA-binding domain, identified in splenic hemangiosarcoma (HSA). Thirteen other variants clustered in introns 2-10 (g.1023Del_T, g.1041G>A, g.1297Ins_C, g.1782Ins_G, g.1794Ins_G, g.1839Del_A, g.1839_A>G, g.1889Del_GA, g.1891G>A, g.2211_A>G, g.4360_T>G, g.4362C>T, g.4363T>C) and four variants (g.158Del_G, g.159Del_G, g.160Del_G and g.317_T>C) were found near the 5′ UTR promoter region (Table 2).

**Table 2:** Detected tumor protein p53 gene variants in canine clinical tumor samples (tissues or blood)

Variant (gene region)	HGVS g.	HGVS c./p	Cases	Sample ID	Tumor types	Sequenced material	Genomic position	Region (intron/exon/UTR)
g.3401insG	g.32668794insG	c.408/p.136ins	1	S-10	HSA	Tissue	chr5:32668794	Exon 5
g.1023delT	g.32666417del	-	3	S-18, S-21, S-32	MCT, OMM, lymphoma	Blood	chr5:32666417	Intron 10
g.1041G>A	g.32666435G>A	-	2	S-15, S-26	MLO, SCC	Tissue (S-15), blood (S-26)	chr5:32666435	Intron 10
g.1297Ins_C	g.32666687insC	-	1	S-8	MGT	Tissue	chr5:32666687	Intron 10
g.1782Ins_G	g.32667173insG	-	1	S-6	MCT	Tissue	chr5:32667173	Intron 9–10
g.1794Ins_G	g.32667186insG	-	1	S-6	MCT	Tissue	chr5:32667186	Intron 9–10
g.1839Del_A	g.32667233del	-	4	S-10, S-21, S-22, S-37	HS, HSA, lymphoma, OMM	Tissue (S-10), blood (S-21, S-22, S-37)	chr5:32667233	Intron 9–10
g.1839A>G	g.32667233A>G	-	1	S-26	SCC	Blood	chr5:32667233	Intron 9–10
g.1889_1890delGA	g.32667283-32667284del	-	2	S-1, S-8	mammary adenoma, MGT	Tissue	chr5:32667283-32667284	Intron 9–10
g.1891G>A	g.32667285G>A	-	5	S-23, S-24, S-25, S-34, S-37	HSA, OSA CGAC, lymphoma	Blood	chr5:32667285	Intron 9–10
g.2211A>G	g.32667603A>G	-	1	S-32	Lymphoma	Blood	chr5:32667603	Intron 8–9
g.4360T>G	g.32669754T>G	-	1	S-5	MCT	Tissue	chr5:32669754	Intron 2–3
g.4362C>T	g.32669755C>T	-	1	S-4	MCT	Tissue	chr5:32669755	Intron 2–3
g.4363T>C	g.32669756T>C	-	1	S-4	MCT	Tissue	chr5:32669756	Intron 2–3
g.158Del_G	g.32665552del	-	2	S-28, S-32	SCC, lymphoma	Blood	chr5:32665552	5′ UTR
g.159Del_G	g.32665553del	-	2	S-28, S-32	SCC, lymphoma	Blood	chr5:32665553	5′ UTR
g.160Del_G	g.32665554del	-	2	S-8, S-32	MGT, lymphoma	Tissue (S-8), blood (S-32)	chr5:32665554	5′ UTR
g.317T>C	g.32665709T>C	-	1	S-32	Lymphoma	Blood	chr5:32665709	5′ UTR

Variants were identified relative to Ensembl ROS_Cfam_1.0_Forward-4.6kb (Supplementary Data 3) reference genomic assembly coordinates; cancer-specific somatic status was not determined using matched normal tissue. The variants such as g.1041G>A (EVA:rs3329107810), g.1839A>G (EVA:rs3329624656), g.2211A>G (EVA:rs850574130), g.317T>C (EVA:rs3329624656), and g.4360T>G (EVA:rs3329624799) were reported as Ensembl variants (Supplementary Data 5).

HSA, hemangiosarcoma; MCT, mast cell tumor; OMM, oral malignant melanoma; MLO, multilobular osteochondrosarcoma; SCC, squamous cell carcinoma; MGT, mammary gland tumor; HS, histiocytic sarcoma; M_Adenoma, mammary adenoma; OSA, osteosarcoma; CGAC, ceruminous gland adenocarcinoma; EVA, European Variation Archive.

Non-coding variants dominated (72%), whereas only 5% affected the coding sequence and 22% in the regulatory 5′ UTR region (Fig. 1C). MCTs, lymphomas, sarcomas (splenic HSA), and carcinomas (SCC) exhibited the highest variant prevalence (Table 2). The g.1891G>A substitution was the most frequent (5/18), identified in HSA, OSA, ceruminous gland adenocarcinoma, and lymphoma. This was followed by g.1839Del_A (4/18) in HSA, oral malignant melanoma (OMM), lymphoma, and histiocytic sarcoma (HS) and g.1023Del_T in HSA, MCT, lymphoma, and OMM, indicating recurrent intronic (intron 9–10) variations particularly in HSA. Notably, g.1891G>A and g.1023Del were observed in high-grade IV and T-zone lymphomas. The g.1839Del_A variant across HSA, OMM, lymphoma, and HS, suggests potential shared variant mechanisms between hematopoietic and mesenchymal tumors (Table 2). Conversely, g.1889-1890Del_GA was detected in both mammary–carcinomas and adenomas, underscoring the need for matched normal controls to distinguish between somatic and breed-associated variants.

Several breeds including Poodles (3/3), Golden Retrievers (2/2), Beagle (1/1), Cocker Spaniel (1/1), Belgian Malinois (1/1), and Shih Tzus (2/2) showed TP53 variants whereas Labrador Retriever (0/1), Welsh Corgi (0/1), and Pomeranian (0/2) did not, followed by Maltese (5/8) and Jindo dogs (1/4) in our small cohort. However, small sample sizes limit the breed-specific conclusions.

## DISCUSSION

Accurate cancer-specific somatic mutation screening requires a robust reference genome to ensure consistent mapping of variants. Comparative alignment identified Ensembl_ROS_Cfam_1.0 as the most reliable reference sequence for canine TP53 gene. Primer-walking PCR approach amplified 4.4kb TP53 region (introns_exons_2–11) and identified 18 distinct types of TP53 nucleotide variants in 26 canine tumor samples, with ~95% in non-coding regions (intronic or 5′ UTR) and one in the coding exon. Variants were observed across several breeds (Maltese, Poodles, and Shih Tzus) and tumor types (MCTs, carcinomas, sarcomas, and lymphomas). These variants were detected in tumor-bearing dogs rather than that of confirmed germline variants, necessitating future studies that incorporate matched tumor/normal samples for biological relevance. However, the predominance of non-coding variants, including recurrent intron 9–10 variants, suggests a potential regulatory region that influences TP53 transcription or splicing. Such variants may contribute to tumor persistence and therapeutic resistance, paralleling human intron 9–10 variants associated with apoptosis evasion and chemoresistance [15], reported significance of intron_9 variants in Li-Fraumeni syndrome families [16], and others [17,18,19,20]. Heterodimers were observed from 1,888–2,160 bp in eight samples. All reported variants were confirmed using bidirectional Sanger reads and manual chromatogram review to minimize artifact inclusions.

The identified exonic splenic_HSA variant represents a conserved DNA-binding region implicated in sarcoma progression across species [3,4,6].

The recurrent intronic (9–10) and 5′ UTR variants might be linked to lymphoma and epithelial tumor susceptibility; however, somatic status must be confirmed for a stronger interpretation. The Jindo and Labrador samples exhibited minimal TP53 variations, implying alternative oncogenic mechanisms. Sarcomas, MCTs, and lymphomas exhibit the highest gene variations, consistent with their genomic instability and TP53-pathway dependence [2,3,6]. Collectively, these findings highlight the fact that both structural and regulatory TP53 alterations drive canine cancer, with intronic variants emerging as potential pan-cancer biomarkers.

Canine cancer-specific gene mutational diagnosis remains challenging. Here demonstrates a feasible, cost-effective PCR-based primer-walking and Sanger sequencing approach for detecting TP53 gene variations in exonic/intronic/regulatory regions of various clinical canine tumor samples. The dominant non-coding variants (introns_8–10 and 5′ UTR) suggest a regulatory mechanism in TP53 dysregulation, which must be validated for somatic/germline status. These emphasize the need to extend oncogenic variant screening beyond exons in oncology, both veterinary and comparative oncology research.

TP53 variants, including those in noncoding regulatory regions, may affect clinical outcomes, such as chemotherapy response, survival, and recurrence risk. Integration of TP53 genotyping into diagnostic workflows could facilitate precision oncology in veterinary medicine, thereby benefiting comparative oncology research.

## SUPPLEMENTARY MATERIALS

Supplementary Data 1 Canis lupus familiaris NCBI_TP53_reference gene FASTA sequences_3

Supplementary Data 2 Canis lupus familiaris_NCBI_ensembl_TP53_reference gene sequences

Supplementary Data 3 >Sequence of ROS_Cfam_1.0:5:32665394:32678755:1_forward_4617 bp

Supplementary Data 4 Histopathological images of hematoxylin and eosin stain of canine tumors at ×400 magnification

Supplementary Data 5 ROS_Cfam_1.0_Forward-Whole length-Genbank file

Supplementary Table 1 Seven pairs of primers to cover TP53 target region (4.4 kb)

Supplementary Table 2 Detailed samples details and sequenced materials

Supplementary Fig. 1 Multiple sequence alignment of three important Dog TP53 references. (A) Multiple sequence alignment of three important Dog TP53 references canine_TP53_ROS_Cfam_1.0_NC_051809.1, canine_TP53_Dog10K_Boxer_Tasha_NC_006587.4:32699559-32713071 and canine_TP53_UU_Cfam_GSD_1.0_NC_049226.1:32768467-32781775 using Clustal Omega. (B) Alignment of canine TP53 13.3KB whole gene sequence from two different database sources Ensembl_114 and NCBI_ROS_Cfam_1.0_NC_051809.1.
