# Supplementary material for: Primer-walking sequencing identifies predominantly noncoding TP53 variants in canine cancers
Source: J Vet Sci. 2026 May 27;27(4):e42. doi: 10.4142/jvs.25325 (PMC13454805; doi:10.4142/jvs.25325)
Supplement: Supplementary file 1 [file jvs-27-e42-s001.doc]

**Supplementary Data 1.** Canis lupus familiaris NCBI_TP53_reference gene FASTA sequences_3

>Canine TP53_ROS_Cfam_1.0_NC_051809.1:32665395-32678721 Canis lupus familiaris isolate SID07034 breed Labrador retriever chromosome 5, whole genome shotgun sequence

GGTAGTAAAGTTTTATTGTAAAATAAGAGATTGATATAAAAACGGGATATAAAAAGGGAAGAGGAGGAGG

GGTGGGGGTGATCAATGCATGAATGCAGATGTGCCTCACAGAGTGCAAAAGAAATTGATCCTGAGCAATT

GGGGGCGGGGGGGGGGGGGAAGCAGTAAAAGCCCTTGGTAGACAAAGATCCTACACCAGGGATGGGATTC

TGCAGGGTGTGAGATGGGAGTGAGATTTCATCTTAGGTGCAGGGGTCTCCCAAAAAGGCTGGACAGGATT

TGGCCAGGGCAGGGAATGAGCACCCACACAGGCACTAAGTCACAGACATGGGATAAAAGGTGGTTTTGGG

GTCAGACAGACATTATTTCATTCATCCTCACAAGCTCAGTGAGGTAGGTATGGTTAATTAGCAATTCATA

AAAATGGCCTGTGAAATTAGGAAAAATTCAGCCACTGGCTACAATGATAGAGAACTGGGCTCATGACTCA

GTGTCTTAGGGGCTATAAAATGCTCCCAACATTCAGGAACATTTTCCTACCTCTCCCATGGTTCTACACA

GTAAAAAACTTAAAAACCAGGCTGGAACGTCCTTTATCTCCCGACCTACCAACTGTGCAGGCCAACTTCT

TTAGTAGAGACTTACGGGAAAGGCAAATGGAAGAATCTAGGGTGTTTCTGAGGCACATCTGTACCATGCA

AAGTAACACACCGGAATAGGTGTGCTCAAGCTCCCAATTCCAAAAATGCCAGGGAAGGAGGACGAGAGTG

AGGGTGTGCGTGTTGGGGGAGGACAGGAAGCAGGGGAAGTCAGTCTGAGTCGGGCCCTTCTCTCTTGAAC

ATCAGTTTTTTATGGCGAGAGGTAGATTGCCCCTTCTTTGCCTTCAGGTGGCTGTTGGGGACACAAGGTG

GAGGGGAAGAGACGATATTAGCAAGTGGGAGCCTCGGGGCTCCTTGTCCTAGATCTAGGCTTGGAAGGGT

CCAAGGCTGGAGTAAGGGAATTTTTTTTTTTTTTTTTTTTTTGGAATGAGGTAATTTAAGTTAAAATGCT

GTAGACACATTTACTGAGCACCCAGGGAACTTCTGTGTGTTCGCTTCCTCTCCCCTCACCCCATCTGATG

AACAAGGAAACCGAGCCCCAGAAGGGGAGATGCAAGGCCACACGGATCTGCTCATCCTTGGCAGGGCTGG

GGGTCTGGCCCAATCCTGCTGTGTCCAGTATTTTCCCACACGGTTATCAGGTCCCTATTGGAGACCCCAC

TTGGCACTAGGGGTTGCTGGAGATGACAAACACCCATGCTTGTCCCTTAAGGGGTTTGGGGACCTCCCGA

GGCAGAAAAGGGAGTACAGGATCCAACTGGGGGTATGGTAGAAGCAGGTTTGCCCAGGGTGGGACCAACC

ATGAGGGGATCAGTTCCCTTCCACCTTGAAGGTAGGAGTAGAATGGAATCCTGTGGCTTTCCAACATATG

AAGGCAAGGGGGTGGGGCTGCAAGCCGGCCCAGGTCACTCACCTGGAGTGAGCCCTGCTTCCCCCTGGCT

CCTTTCCACTCTGGGCATCCTTCAGCTCCAAGGCTTCATTCAGATTCCTGAACATCTCATAGCGTTCACG

CCCACGGATCTGTGGCAGTGGGGGGGAGGAGGAGAAAGAATAAGAGGGGGAAGCCACAGTACCATTTAAC

TCAGACTAAGGTGTAAGTGCCAATCTGCAGCAAGGCACAGCCCGCAGCCACTGTCAAGGCACCGCTGCCC

CTTGATGGCAAGTGTGCTAATTGCAGGCGGAGCAGTCAAAAGAAAACTGTGCTCTTGGCCCTCAGCTTGA

AACAAATCTTCAATCCAAGAATATCAACTTCAAGAATCAGGGACAGGACCAGAGAAAAGGATGGAGAGAG

GAACAGGCCTAGCCTCACATGTGCTCCATTCACATTTTTTTCTTCCCTCATCTGATCCCGCTAAGCGGGG

GCAAGAGCCTGCCTTATCTGTTCCTCCCAGCCTTGGTACCTGAAGGGTGAAATATTCTCCATCTAGTGGC

TTCTTCTTTTGCGGGGGAGAGGAGCTGGTGCTGGGAGGCAGTGCTGGGAAAGAGAGGAGGAAAAGACAAG

AGAAGAGTATGTTTTGAGCAGAATTGCACCCTCTGTATCTGCCCCACATCCTCCTCTTTTTTTTTTTTTT

TTCAAGATTATATTTATTTATTCATGAGACATACACACAAAGGCAAAAAAATAGGCAGAGGGAGAGGCAG

GCTCCCTACAGGGACCCTGACACGGGACTCGATCCCAGGACCCTGGGATCATGCCTGATGCTCAACCCCT

GAGCCACCCAGGTGTCCCTCCTTCACCTCCTCTTGTCTTGCTTGCTTACCTCGCTTGGTACTCCCGGGGG

GTGGCTCAGGACAAGGCTCCCCCTTCTTGTGGAAATTCTCCTCCTCAGTCCGGCGGTCTCTCCCGGGACA

GGCACAAACGCGTACCTCAAAGCTGTTGCGTCCCAGCACGTTTCCACTACCCAGGTGAGAAGAGAGAAGC

AGGGGGCCGTGAGGGTGGTGGGCCCCACTGACTCCTAGCCACCCTCATCCTACGTAGAGCCAAAGCCTAC

AGACTATTGAGAAGCCACAGGTCAGGGAGGTCCCAAAGCCAGAGGAAAGAAAACCAGGGGAGGCGGATGG

CAGAGGAGATTCCACATTGGGACAGGAATGGATGGGAAGGATGGAGGGGGGTACAGTAAGGAAGTGGCCA

GGAGCAGGGTGGCAGGCAGGTCCCTACCTGGAGTCTTCCAGGGTGATGATAGTGAGGATGGGCCGCCGGT

TCATGCCTCCCATGCAGGAACTGTTACACATGTAGTTGTAGTGGATGGTGGTATAGTCAGAGCCAACCTG

AGGTAGAAGGTAGGCCCAGGGTCGGGCCACTCAGCCCTGGGCAGGCCTGTAGTCTATCAGCCTCAGTCTT

TCCCAACCGCAGAATGGGATGACCCACCTGCCTCTGCACTCCTCACAGGGCTGCGGGGAGGTTGAAGGAG

GAGAAAGCCCCCTCAACTCCCGCCTCACAAATCCCTCCAGCCCCCTCCTCCCAGAGACCCCTCAGATGCC

AAACCAGACCTCGGGTGGCTCATAAGGCACCACCACACTGTGTCGAAAAGTGTTTCTGTCGTCCAGGTAC

TTGGCCCGCAAATTTCCTTCCACTCGGATGAGATGCTGAGGAGGGGCAAGACCTAAGAGCCATCGGGGAG

GAATCAGGGCTTTGGGCCCTCTGGGGAGCAGCCTTGTCCCATCTGTAGCCCCCGATGGCTTACCGTCACT

ACTGTCAGAGCAGCGTTCATGGTGGGGGCAGCGCCGCACAACCTCGGTCACGAACTCCGACTTCTTATAG

ATGGCCATAGCGCGGACGCAGGTATTGGGTGGGGGTGGGGAGCTGACCCACAGCTGCACGGGGCAGGTCT

TCGCCAGCTGGCAAAACAACTTGTTGAGGAGAGGGGAGTACTGTAAGAAAAGGACAGATGGACAGGTCAG

GGCATGTGTGCCCGGGGTGGGTGGTGAGAAGTAGAGGGGTCGGGGTCTATTCCAGAAGCTTACTTTATTT

TAGACACACAGAGAGAGGCAGAGACACAGGCAGAGGGAGAAGTGGTATGGGATTCGATCCCAGGACCTGG

GGCTCACACCCTGAACCAAAGGCAGACGCTCAACTGCTGAGCTGCCCAGGCATCTCCCAGAAGCTTACTT

TAAAAGCCAAGGAATGAACAAGTGAAGGAAAATGAAGAAGAGTCCCTTTGCCCCATGACAGGAAAGGTGA

AAGCCCAAGGTCAAGGGGGGAAATGTCAACTTTGACACAGAGCTGAGGCCAAAGGTCACAGAATACATAG

GTAATGCAGGAAGACTGACAGTGCGGACCGAGAGGAGTCTCAAAATCCCAAACAGAGAAACCCAGGGGCC

ACAGCCAGGCCCCGGCCAGCCCCATGGAAACCCGCCTCGCAGGGCCACTGACCGTCCAAGTAACAGACTT

GGCTGTCCCGGAATGCAGGAACCCCAAACGGAACCCATAGGTGCCAGGGTAGGTCTTCGGGGAAGGGACA

GAGGATGATAGGGGCCAGGAGGGGGCCGGTCCAGGGGCTGTGGGGGCAGAAGTGGCTGGCATCCTGGGAG

CATCATCTGAGTCTTCGTCTAGCCAGTTCACGACGCTCTCTGGGAGCAGCAGCTCATCCACTGCTGGGCA

CAGCTCCGAAGACTGTAGGGGGCGAGACCAGAGTCAAGGGTCAGGTCCCTAGGTCCCCAGCTCCCAACCC

CCGCCCTGGACCTCCCCACACCCAGTCCTTACCAGAACATTGTTTTCAGGAAGCCTGGAAGATGAGAGCA

GAAAGTCAGTGCTGGTTTTGCTTCCTCTGGGCTTCTACTTGGGGGGGCGGGGGGGGGGGCTGGGGCTGGG

GGTTGGGATTGGGGTGGGTGGGCAGGGGTCCCAGAGGGCTGGTGGGGAGCCTGGCCTGCCCTCCACTTAC

AGGTTCCACAATTCTGAAAATGTCTCCTGGCTCAGAGGGGGGTCGATATTGAGCTCTGACTGTGGCTCTT

GCATCGCAGCTCCTGGGAAGGGAGTGTGGGTGCTGTGGAGGGCAAGGCGAGGGGTCAGCGGGAGACAAGC

CCGGGCCCGGGTCCCTCAGCCCCGAGGTGGGAGGGACCCTTCCAGCAACATTATACCAGGGCCTGGGATG

CCTTGGCTCCCCCACCTGGGCCTCCCCAGGAGCAGGGCTGCTCCTGCCCATTCCACCCCTGGGCGGCCTA

CCCCCACACATCCTCAAGGTACAAAGATAACCAGAAAGGGGATTCTAGCCCTCTCATTACAGAGTCCACA

AGCCTTCCTGGTTATAAGGATGAAGAACGTCAGCCCACACCCTCCACCTGGACGAAGATACCTGCATGGA

CCCCCTCATTCCCCACCTGATCCCCTTCATCACGAAGCTTCCTAAGGATCTGCTATCTCCATTCCACCCT

TGGCTTGCAGAGTCTTCAGGACCTCCCGGCCGGCATCCCCGACCTTGTCTTCCAGTATTCTCCAGCCAGT

GGCTGCCCTTCTCTGCTGCCACACACACACCCCAGTTCATTGGCTCGGGGTTCCCTCTATTGGGAACCCT

GGTCCAGCCTCTGCTTGCAGGGGAGTCACCTTCTAGCAGATCATCCCGAATGTCACAGCTCCTCAGGGAT

GTCTGCCCAGACCACCCAGGCAAAGACTGCCAGCCAAACGCTGTCACCAGTTTTTTTAAAAAAGATTTGA

TTAATTAATTTATTTGAGAGAGAGAACAAGAGTGAGAGAGTGGCAGAGGGAGGGGGAGAGGGAGAAGCAG

GTGAGCTACCTGTCCGGCTCACTGCAGCACTAGCAAAATGCCTCTCACCTGGTTGGGCTCACTGTGGTCA

GCAAGTGAATGGTGCCGAAGCCAGGAGCACCTTGTGAGGTGTCCCCAGGAGCCAGCAGGTCAGAGGGAAG

GTCCCAGTCTGGGCCCTGGCGCTGTACTAGGCTGCGCTGCCACCATCTCATGACATCTTCCCCAACTCCC

GAGTTTGTCAAGAGTTTTGCAAAAAAACACACCCACTGCAAACCAAAGTCCCTATTCATTACCTTTTTGT

CTTGTAAAGCACTGACAAGCCTACCGGCCCCACCCTCACCCCCAGCAGCTCTTCCCATTAGCATCTCCAT

GAACATTCACCTCTACCTGGATTGAAGGCCTGCTTTCTTATGTCCTCAGAGTTTGTGAAATCCTTTCTTG

TGGGTTATCTCATTTGTCCCAGAACCACCCTGGCTGCCCAACAGGTGAGGAAGCTGGTCTCAGAGAGGTG

GAGGGACTCGCCCACCGCACACAGGCAAATGGATGAGCTTTTATTCGAAATGACCACTCGTGGGGTGCTT

GGGTTGCTCAGTGGTGGAGCACCTGCCTTTGGCTCAGGTTGTGAGCCAAAGGGTCCTGGGATCGAGTCCC

GCACTGGGGTCCCTGTAGGGAGACTGCTTTTCCCTCTGCCCGTGACTCTGCCGCTCTGTGTCTCTCATGA

ATAAATAAAATTTTAAAAAAAGCTTTTGAGATTTTATGGAAGGGAGCTCAGAAGTCAAAGCCCTAGGTCT

GACATCGTATCATTACTTATATTTGCTGTGCAACTCTACCCATCCCAAGGGCTAGCCTTTTGGCTTCCTT

ACATGCAGGAATTGAGTTTTGCTCATCTTTGTTTAGTGAAAAATACCTAGGATGGGGAGACATGAGCAGC

TGTAGCAGGGCATGGGCTCTGAGGCAGACAGGCTAGGCCTAAATCCCATGCCCACCACCTAATAGGTGGG

GAGGTTACTTAACCTCTGTGCCTGGCACAGTAAGAGCTCAACAGATGTGTTTGTTTATAGGATTTTCCAT

TCTATTAGTTTGATTATTATTTTAGACCCAGAACTTTGTAATTGAGTGGGTGGGGCTTCTGTAGTCCTGC

CTCATTAATGCTAGTGCCCCTCTTTCAGGGATCTCTGTTCAGTAAATATGCCAGGAGGGGCTGTCCTGGG

ATGCTAGGCCCGAGGTCCCAATCCCACTGGGAGGGAGACAGCCTCATAGACAAAGGAGGTCTCTCCCTCT

CTTGGTCCTTGGCAGCAAGTAAATTCTGTTTTTCTCAAAAAAGCAGGCACTATCACCAGGAAACCTTTTT

GGAGCATGTACTTCCCATGGATGAGGATCAAGGGCCATTAAACTAAATTGTCAGAAGCTCAACCAGACCT

TCCAAATGGCTGGGGAGGCAGGACTCATAGATATCCCTGGTGGGAAGGCCAAACTCAGCTGGGACCTCAT

CCAGTTGCCCACCCCACCACCATCCTAACAATGGCTGTTGTTTGTAATCCGCACAGGGGCTCACTGCTCC

GGTGGCTTATCTCATTAATCCTCTCAAGAAACCATAGCAGTGGGTATCTTCAAGCTGAAGGAACTGAGGT

TGAAGGAATTAACTCACTCATTCAGATTCAGAAAACTCCCGAGCCAGGGATTTGAATCTGGGCTGTTCAA

AAGCCACTGTCCAAGCTCTAAATAATTATGCTCCTCTGGCTTCATCCAGAGTCTGTAAAAATAATAATAA

TAATAATATTTGTTTGGTGCAAAAAGGTTTTTTTGGCTGAAAGCTATTTGGGAAAACACGATGATTCTTA

AAAGCAGCAAGGCAGTATCTGGTAAGACTCAACAACTTACTACTTTGCAGAATCACTTCTAGGTAAGACT

CCCTTAGAAGAGTTTTGTATGGAGGGAGCAGGAGTTCTGCGCAAATACGTTCATATCAGTGATGTTCTCC

GTGACAGGAAACATCACTATCCATCCGTAGGGATTGGCTACATGAAATACAAGAGTTTACTGCAGCAGTT

TAAGGAAATGAATGTATCTGTGTGTACGTAACTAGATAGATATAGCTCAGATTGATTAAAAAAATACAAT

TCGAGGGGGAAAAAAAAGAAAAACATGGTAACCTAAGTAGTTTATGTGAATTAAAAATATAAAATGAGTA

CAGTTGACCCTTGAATAATGTGGGGGTTGGGGGCATCGAACCCTGTGCTATAGAAAATTCATGAGTTGGG

GCACCTGAGTGGCTCAGTGGCTGAGCTTCTGTCTTGGGCTCAGGTCGTGATCCTGGGGTCCTGGAATCGA

GTCCCGCATCAGGATCCCCACAGGGAGACTGCTTCTCCCTCTGCCTATGTCTCTGCCTCTCATGAATAAA

TACATTTAAAAAGTCTTAAAAAAAAGGGATCCCTGGGTGGCGCAGCGGTTTGGTGCCTGCCTTTGGCCCA

GGGCGCGATCCTGGAGACCCGGGATCAAATCCCACGTCGGGCTCCCTGCATAGAGCCTGCTTCTCCCTCT

GCCTATGTCTCTCTCTCTCTCTCTCTCTCTCTCTCTCTCTCTCTGTGACTACCATAAATAAATAAAAATT

AAGAAAAAAAAAAGTCTTAAAAAAAAAAAATCCAGGGACGCCTGGGTGGCTCAGTGGTTGAGTGTCTGCC

TTTGGCCCAGGGCATGATCCTGGGGTGCCCAGATGGAGTCCCGCATGGAGCCTGCTTCTCTTTACCTGTG

TCTCTGCCTCTCTCTCTGTGTCTCTCATGAATAAATAAATAAAATCTTAAAAAAAAAAAAAATCCAGGTG

TTATCTCTTGACTGACCCAAAATTTAACAGCTTACTATTGAGAAGCCTTACCGATAACATAAGTAACTAA

AACATATTATATATATATATTATATACTACATTCTTTTTTTAAAAAAAGATTTTATTTATTTGAGAGGGA

GAGAAGCAGACTACCCGCTGAGCAGAGAGCTTGATGCAGGGCTGGATCCCAGGACCCTAAGATAGTGACC

TGAGCTAAAGGCAGACTGGCCACCTAGGTGCCCCCCATATACTATATTCTTAAAGTAAGCTAGAGATAAT

GCTATTAATATAATCACAAGGAAAAAAAGAAACATTCACAATACTGTTACTGTATTTATCCAAAAAACCC

ATGTATAAGTGGTCCTACACAGTTCAATCCTGTGTTTTCTAAGGGTCAACTGAATTTATTTCTTACCAAC

ATATATATGTGAAAGTATGAAAGACAGATAAAAAGAATTAAAACTTACACTCATGAGACTGGGGGTACCA

TCAAAGCGACCTTATTTGATTAATGAAGAAAAATTTAAAAAGATGGTATTCATACATTATTTGTTTATTA

TAATATGAACATGACAAAATGTCAAGTGTTATAATTTCCTCTAGCTTCAAGAATAATCAACATTCTGCCT

GTATTGTCTCCCCTATACTTCTAGTGGCTCCGCAGAAGCCCACAGATATTTTTGAGTTACAATCTATGTA

AGTTGAAATGCACAATCTTAATTTTGCCATTTAGATAACAAAGTTTGACACATTTTGATATAACCTTGTC

ACCCAGCCCTACCAAGCATAAGACACTGCTGCCATGTTTCCTTCTGAAAGTTAATATATTTTATGCTTAG

ATCTACTATCCATCTCAAATTGTGTATAGTATGAGGTAGGGGTCAGAATCCACTCAGCTCTGCTTGTTTC

AGCACTCTCTTCAGACTTTCCTTCCCCCATGGATTCATTTTGGAGCTTTTGTAGAAAACCATTTGATTAC

ACTTATGTGTCTTTTCCTGGGCTCTATTCAGTTCATGGTTGTTGTCTTATGGTAGCTTTACAGAATTCTT

GAAGTCAGCTAGTTTAAGACCTCCAGCTTTGTACTACACTTTCATTTTGGAATTGCTTGTCAGTTTCTAC

AAACAAAACCAGCTAGGATTTTGATGGGGATCAAATGAAATCATCTTGGAGTACTGACATCTCAACACTA

TTGAGTGTTCCATCTATGAATATGGTACTGTTACTTAAATCTTCCGGGCAGCCCGGGTGGCTCAGCGGTT

TGGCACTGCCTTCGGCCTGGGGTGTGGTTCTGGGGACCAGGGATCGAGTCCCACGCAGGCTCCCTGCGTG

GGGCCTGCTTCTTCCTCTGCCTGTGTCTCTGCCTCTCACTTTGTGTCTCTCATTAATAAACAAATAAAAT

AAAATAAAATAAAAATCTTCCTTAAATTCGCTCAGCAATATTTTGCAGTTTCTAACATAGAACTCTTGTC

CGTCTTTCTTCAATTTATCCCTGGGTATGCCATGTTTTTGGATGCTTTGTAAATGCCACTGCTTTTATAA

TTCATTTTCCGACTTCAGATTCAATGGTTGTTATTACATAAAAATACAATTTATGTATATTGCCTTTGTA

ACCTGTGACCTTGCTATAATCCTTTATCAGTCATGATAGTTCTTCTTTTTAAGAGGTTTCATTTTTTAAG

TAATCTCTGCATCCAAGGTGGGCCTCAAATTTACAACTCTGAGATCAAGAGTCATGTATTCTACTGACTG

AGCCAACCAGGTGCCCCTTGTGAGTTTTTCAATGCTTCTTCTATTAGCCTGGTTAACACCTTCAGTATAA

TGTTGAAGGGGTGAGAATCAAATCCTTTCCTAGTTCCTCATCTTAGAAAACAAGCATTCAGTCTGTCACT

GTTACGTATTGATGGTAACTTGATTGGGGGTGCTCTTTATCAGACTAAGTTTTCCCTTTCCTAGTTTGCT

GGGAGGTTGGCTTTTCTTTTCTTTCTTTTTTTAAAATCATGAATAGGTGTTGCGTGATGTCAACTGCTTT

TTATGTATCTGTTGAAATGATCATAGGATTTTTATCCTTCAATCTGTTTATGTGGCAAATTACACTGATT

TTTTGAATGTTAAAACATTGCCGTCTAGAGATAAACTCCACTTGATTAGGATGTATTTATCCGTTTTATA

GATAATCAGATTTACTTTGCTAAGATTTGTGAGATTTTTGCATATATACATATGTGTGTATATATACACA

CATATGTATATATGCTGGCAGGAATATTGGTCTAAATGTTTTCTTGTAACATTCTCGTCTGGTTTTGATA

TGAGGATTCTCAGGATATCATGAAATTAGTTGGGAAGATGTTACCTCTTCTACATTCTGAAAGTTTTTGT

AAGATTGTTATTGTTTCTTCCTCAAGTGTCTGAAGAGAAGAAGAAATAGAATAGAAAATAGCATTAACAC

AGTAGACTTAAACCCAAACCTTACAATAATTGCATTAAATTAAATAGACCAAGCACTCCAATTAAAAGTG

ATCCAGTGAGGGGGGAAGGAGGGCTGTTTCAACAAGTGGCACTAGAAGAACTGGATATACATCTAGGAAA

ACCCCAAAAGAAAACAGAACCCCTACCTCACATCACGATAAAAATTACTCTTAATTAATTAGTTAATTAA

TTATGGCTCAAAGACCTAAATATAAAACCTAAAACCCTAAAGATTCTGGAAGAAAATATCCTAGAATATC

TTCATGATCTTGGGGTCAGCAAGGATTTTCTAAACACACAGAACACATTTGAAAGACAGAAGATAAATCT

GCTGCTGCTCTGCCTTAGAGGCTGACAGAGAGGCAAGAAACAGGTCAGGAGAAAATAGATACTGACAAAA

GACTCCTATCCGGAATATATAAAGAACTGCATGCAGCTGAACGCAAGTTAAAATGGCCAATAAGCACGTG

GAAACTGATCACAGCTAAGAGAATTGCAACTGAAAACCACAATGATGCACCACATTAGAAAGTCTGACAA

AACCAGATGTGAGGAAAGACAGCTGTTGCTGGTGGGTACATAAAACAGAACAACCACTTTGAAATATACT

AATTTTAAAAATCAAATTTAGATCTCTCTCACCTAGCAAATTCCACTCCCAGACGGTTATAAAAAACAAA

AACAAAACAAAACAAAACACACACACACACAAACACCCAAAACCAACCCCATGAAAACATAGGACCACAA

AGAGAATTGTACAAGAATGTTAATAGCAGCTTTATTCAAAATAACCCCGAACGGTTAACAACCCAAGCGG

TCACCGAAAGGAACATGGATTAAAAAAAACCTCACGGTTTATTCATCCAGTGAGATGTGCTAAATACAAA

AATACAAAGGTGCAAAAAACACTGATACACTACTGATACAGTAGTACTGGTTACACAGGAACAAGGATGG

GTGTCACAGGTATTATGCTGCCGGCGAGAAGCCTACACAAAGGAGCACACACTATGTGGGTCTATTTACA

CGACGCTCTCTTCAACAGGCACAAGTAGTGACAGAACTCCAAGATGAGGGTCAGAGACCCCCAGAGGGGA

GAGGCCCAAGAACCTTCTCTGGGGCGATGACAATGTGTAAATTGTGTCATTTGACAGGCTGCTGGTTAGC

AGCCCAAACCTGTCTAATTGCAGTGAGTGTAAATGCTACCTCAATTTCAGAACAAGCCGCACACAAGCAC

CGCGTGCACTGGCCGGTACCGAGCAGGTGCACAGGTAGGTGAGCGAGCGCAGGCTGGAAGCACCGCGCGC

CCGGAGTGACTGACAGGCGGGAGGCGGTATCCCGTCCCCTCGGCTCGGCTCGGCTCGGCTCGGCTCGGCG

GGCCCCGCCCCCTCAAGCCCCGCCCCTTCCGCCCTCACGACGTTCGCTCTGCGTCGGAACTCGGAATGGC

TGAGTCCCGAGCCGGATGCCAGAGAAACCGTGTCTCGGCCCCACACAAACGGCGCATCGTCGCATTCTGC

CATCGTTGTCATTCTCAAATGTACTCGTGCTCGAAAATACCCGGGACTTGCCGTCGGCACGCTTCTGGAG

ACCTCGTCCCTCGCCCGCCCAGATTGCAGGCGATGGGGTGGGGCGCACCATTCAAGGAGTGGGGGAAGGC

TCGAGGTTTGCATCCAAACAGACCCTCCCTCCCGGCCTGCCAAAAGTAGCAGCTAAGGAGTTCAGGGGAA

AAAAAAAAAAAAAAAAAAAAAAAAAGAAAGAAAAGAAAAAATAAGAAAGAAAAAAGAAAAGAAAAGAAAA

GAAAAGAAAGCCAGTAGAGGAGGCGGCGCGCAGCCTCCCTGAGCACGTCAGTCTAAATGACACTTTCCCT

CTCCTCGGGGGAACAGTGAACTGCTCCGGAAAAAAAACCGGAAACGCTGCGGCAAGGGGCAGAAATGTAA

ATGTTCGACCAAACAATAACAGAGCTCGCGGGCCTCCTGGACCCGGACCGTCCCGCCGGGCGTCGCGGGC

AGACTGCTCGTGGAGCGCTACCCACGTCCACGCCAGGCAGTCTACTAGGATCGTCTCCATTTAGTGGAGC

CAGGGGCCGCTGGGGAGATCCCTCCGGGAAGGACAAAGGTCCGGAAGTTCTGGGACCTGAGCGGCTTGGG

CTTCCTGGATCGACCCCCCCGAATGTGATCATGTCGGGACGGAGCCCGAGAGGACCCTCCAGGGATGCTG

CGGGCTCGAAGATCTCGGGGGTCCCCAAAATCCCGAGATCGGGGAAAAAACAATCTCGGATCCTGATGCT

TTGTGCGTCTTATTTCGGGGTTCTTCACACCCGGCACGGGGAGAGAAAGGGAGGGGAAGGGTGAGGAGGA

ACCCCTTCGGCCGGCTGCACGTCCCCCTCCTATAAAGCTGAGTCCGGCTGCTCCCAACAGCCCCTTCATC

TGCACCCAGGCCTCCCAGCGGTGCATTCCGATATTGGTGCCGCCAGCCAAACTTCCATTCCCCCGCGATC

GCTGATCGCGTGCCCCACCCCCAGACCCCAATTCTCCCCAAGCTCAAAACACACAGAGCCGAGAGCCGGT

GACTCAGACAGGACTCATCTGGCTCAGTCAGGAGCTTACCCGAACCTCAGGGGAGCGTGTCACCGTCCTG

GGAAGCACGCTCCGAGGCCGGACGCCAGAGGATCCCGGAGCCCGGCGGCTACCTGCGCCCAGGATGGTGG

CTCTGGACTTTTGAGAAGCTCAAAACT

>Canine TP53_Dog10K_Boxer_Tasha_NC_006587.4:32699559-32713071 Canis lupus familiaris isolate Tasha breed boxer chromosome 5, alternate assembly, whole genome shotgun sequence

GGTAGTAAAGTTTTATTGTAAAATAAGAGATTGATATAAAAACGGGATATAAAAAGGGAAGAGGAGGAGG

GGTGGGGGTGATCAATGCATGAATGCAGATGTGCCTCACAGAGTGCAAAAGAAATTGATCCTGAGCAATT

GGGGGCGGGGGGGGGGGGGAAGCAGTAAAAGCCCTTGGTAGACAAAGATCCTACACCAGGGATGGGATTC

TGCAGGGTGTGAGATGGGAGTGAGATTTCATCTTAGGTGCAGGGGTCTCCCAAAAAGGCTGGACAGGATT

TGGCCAGGGCAGGGAATGAGCACCCACACAGGCACTAAGTCACAGACATGGGATAAAAGGTGGTTTTGGG

GTCAGACAGACATTATTTCATTCATCCTCACAAGCTCAGTGAGGTAGGTATGGTTAATTAGCAATTCATA

AAAATGGCCTGTGAAATTAGGAAAAATTCAGCCACTGGCTACAATGATAGAGAACTGGGCTCATGACTCA

GTGTCTTAGGGGCTATAAAATGCTCCCAACATTCAGGAACATTTTCCTACCTCTCCCATGGTTCTACACA

GTAAAAAACTTAAAAACCAGGCTGGAACGTCCTTTATCTCCCGACCTACCAACTGTGCAGGCCAACTTCT

TTAGTAGAGACTTACGGGAAAGGCAAATGGAAGAATCTAGGGTGTTTCTGAGGCACATCTGTACCATGCA

AAGTAACACACCGGAATAGGTGTGCTCAAGCTCCCAATTCCAAAAATGCCAGGGAAGGAGGACGAGAGTG

AGGGTGTGCGTGTTGGGGGAGGACAGGAAGCAGGGGAAGTCAGTCTGAGTCGGGCCCTTCTCTCTTGAAC

ATCAGTTTTTTATGGCGAGAGGTAGATTGCCCCTTCTTTGCCTTCAGGTGGCTGTTGGGGACACAAGGTG

GAGGGGAAGAGACGATATTAGCAAGTGGGAGCCTCGGGGCTCCTTGTCCTAGATCTAGGCTTGGAAGGGT

CCAAGGCTGGAGTAAGGGAATTTTTTTTTTTTTTTTTTTTTGGAATGAGGTAATTTAAGTTAAAATGCTG

TAGACACATTTACTGAGCACCCAGGGAACTTCTGTGTGTTCGCTTCCTCTCCCCTCACCCCATCTGATGA

ACAAGGAAACCGAGCCCCAGAAGGGGAGATGCAAGGCCACACGGATCTGCTCATCCTTGGCAGGGCTGGG

GGTCTGGCCCAATCCTGCTGTGTCCAGTATTTTCCCACACGGTTATCAGGTCCCTATTGGAGACCCCACT

TGGCACTAGGGGTTGCTGGAGATGACAAACACCCATGCTTGTCCCTTAAGGGGTTTGGGGACCTCCCGAG

GCAGAAAAGGGAGTACAGGATCCAACTGGGGGTATGGTAGAAGCAGGTTTGCCCAGGGTGGGACCAACCA

TGAGGGGATCAGTTCCCTTCCACCTTGAAGGTAGGAGTAGAATGGAATCCTGTGGCTTTCCAACATATGA

AGGCAAGGGGGTGGGGCTGCAAGCCGGCCCAGGTCACTCACCTGGAGTGAGCCCTGCTTCCCCCTGGCTC

CTTTCCACTCTGGGCATCCTTCAGCTCCAAGGCTTCATTCAGATTCCTGAACATCTCATAGCGTTCACGC

CCACGGATCTGTGGCAGTGGGGGGGAGGAGGAGAAAGAATAAGAGGGGGAAGCCACAGTACCATTTAACT

CAGACTAAGGTGTAAGTGCCAATCTGCAGCAAGGCACAGCCCGCAGCCACTGTCAAGGCACCGCTGCCCC

TTGATGGCAAGTGTGCTAATTGCAGGCGGAGCAGTCAAAAGAAAACTGTGCTCTTGGCCCTCAGCTTGAA

ACAAATCTTCAATCCAAGAATATCAACTTCAAGAATCAGGGACAGGACCAGAGAAAAGGATGGAGAGGAA

CAGGCCTAGCCTCACATGTGCTCCATTCACATTTTTTTCTTCCCTCATCTGATCCCGCTAAGCGGGGGCA

AGAGCCTGCCTTATCTGTTCCTCCCAGCCTTGGTACCTGAAGGGTGAAATATTCTCCATCTAGTGGCTTC

TTCTTTTGCGGGGGAGAGGAGCTGGTGCTGGGAGGCAGTGCTGGGAAAGAGAGGAGGAAAAGACAAGAGA

AGAGTATGTTTTGAGCAGAATTGCACCCTCTGTATCTGCCCCACATCCTCCTCTTTTTTTTTTTTTTTTC

AAGATTATATTTATTTATTCATGAGACATACACACAAAGGCAAAAAAATAGGCAGAGGGAGAGGCAGGCT

CCCTACAGGGACCCTGACACGGGACTCGATCCCAGGACCCTGGGATCATGCCTGATGCTCAACCCCTGAG

CCACCCAGGTGTCCCTCCTTCACCTCCTCTTGTCTTGCTTGCTTACCTCGCTTGGTACTCCCGGGGGGTG

GCTCAGGACAAGGCTCCCCCTTCTTGTGGAAATTCTCCTCCTCAGTCCGGCGGTCTCTCCCGGGACAGGC

ACAAACGCGTACCTCAAAGCTGTTGCGTCCCAGCACGTTTCCACTACCCAGGTGAGAAGAGAGAAGCAGG

GGGCCGTGAGGGTGGTGGGCCCCACTGACTCCTAGCCACCCTCATCCTACGTAGAGCCAAAGCCTACAGA

CTATTGAGAAGCCACAGGTCAGGGAGGTCCCAAAGCCAGAGGAAAGAAAACCAGGGGAGGCGGATGGCAG

AGGAGATTCCACATTGGGACAGGAATGGATGGGAAGGATGGAGGGGGGTACAGTAAGGAAGTGGCCAGGA

GCAGGGTGGCAGGCAGGTCCCTACCTGGAGTCTTCCAGGGTGATGATAGTGAGGATGGGCCGCCGGTTCA

TGCCTCCCATGCAGGAACTGTTACACATGTAGTTGTAGTGGATGGTGGTATAGTCAGAGCCAACCTGAGG

TAGAAGGTAGGCCCAGGGTCGGGCCACTCAGCCCTGGGCAGGCCTGTAGTCTATCAGCCTCAGTCTTTCC

CAACCGCAGAATGGGATGACCCACCTGCCTCTGCACTCCTCACAGGGCTGCGGGGAGGTTGAAGGAGGAG

AAAGCCCCCTCAACTCCCGCCTCACAAATCCCTCCAGCCCCCTCCTCCCAGAGACCCCTCAGATGCCAAA

CCAGACCTCGGGTGGCTCATAAGGCACCACCACACTGTGTCGAAAAGTGTTTCTGTCGTCCAGGTACTTG

GCCCGCAAATTTCCTTCCACTCGGATGAGATGCTGAGGAGGGGCAAGACCTAAGAGCCATCGGGGAGGAA

TCAGGGCTTTGGGCCCTCTGGGGAGCAGCCTTGTCCCATCTGTAGCCCCCGATGGCTTACCGTCACTACT

GTCAGAGCAGCGTTCATGGTGGGGGCAGCGCCGCACAACCTCGGTCACGAACTCCGACTTCTTATAGATG

GCCATAGCGCGGACGCAGGTATTGGGTGGGGGTGGGGAGCTGACCCACAGCTGCACGGGGCAGGTCTTCG

CCAGCTGGCAAAACAACTTGTTGAGGAGAGGGGAGTACTGTAAGAAAAGGACAGATGGACAGGTCAGGGC

ATGTGTGCCCGGGGTGGGTGGTGAGAAGTAGAGGGGTCGGGGTCTATTCCAGAAGCTTACTTTATTTTAG

ACACACAGAGAGAGGCAGAGACACAGGCAGAGGGAGAAGTGGTATGGGATTCGATCCCAGGACCTGGGGC

TCACACCCTGAACCAAAGGCAGACGCTCAACTGCTGAGCTGCCCAGGCATCTCCCAGAAGCTTACTTTAA

AAGCCAAGGAATGAACAAGTGAAGGAAAATGAAGAAGAGTCCCTTTGCCCCATGACAGGAAAGGTGAAAG

CCCAAGGTCAAGGGGGGAAATGTCAACTTTGACACAGAGCTGAGGCCAAAGGTCACAGAATACATAGGTA

ATGCAGGAAGACTGACAGTGCGGACCGAGAGGAGTCTCAAAATCCCAAACAGAGAAACCCAGGGGCCACA

GCCAGGCCCCGGCCAGCCCCATGGAAACCCGCCTCGCAGGGCCACTGACCGTCCAAGTAACAGACTTGGC

TGTCCCGGAATGCAGGAACCCCAAACGGAACCCATAGGTGCCAGGGTAGGTCTTCGGGGAAGGGACAGAG

GATGATAGGGGCCAGGAGGGGGCCGGTCCAGGGGCTGTGGGGGCAGAAGTGGCTGGCATCCTGGGAGCAT

CATCTGAGTCTTCGTCTAGCCAGTTCACGACGCTCTCTGGGAGCAGCAGCTCATCCACTGCTGGGCACAG

CTCCGAAGACTGTAGGGGGCGAGACCAGAGTCAAGGGTCAGGTCCCTAGGTCCCCAGCTCCCAACCCCCG

CCCTGGACCTCCCCACACCCAGTCCTTACCAGAACATTGTTTTCAGGAAGCCTGGAAGATGAGAGCAGAA

AGTCAGTGCTGGTTTTGCTTCCTCTGGGCTTCTACTTGGGGGGGCGGGGGGGGGGGTGCTGGGGGTTGGG

ATTGGGGTGGGTGGGCAGGGGTCCCAGAGGGCTGGTGGGGAGCCTGGCCTGCCCTCCACTTACAGGTTCC

ACAATTCTGAAAATGTCTCCTGGCTCAGAGGGGGGTCGATATTGAGCTCTGACTGTGGCTCTTGCATCGC

AGCTCCTGGGAAGGGAGTGTGGGTGCTGTGGAGGGCAAGGCGAGGGGTCAGCGGGAGACAAGCCCGGGCC

CGGGTCCCTCAGCCCCGAGGTGGGAGGGACCCTTCCAGCAACATTATACCAGGGCCTGGGATGCCTTGGC

TCCCCCACCTGGGCCTCCCCAGGAGCAGGGCTGCTCCTGCCCATTCCACCCCTGGGCGGCCTACCCCCAC

ACATCCTCAAGGTACAAAGATAACCAGAAAGGGGATTCTAGCCCTCTCATTACAGAGTCCACAAGCCTTC

CTGGTTATAAGGATGAAGAACGTCAGCCCACACCCTCCACCTGGACGAAGATACCTGCATGGACCCCCTC

ATTCCCCACCTGATCCCCTTCATCACGAAGCTTCCTAAGGATCTGCTATCTCCATTCCACCCTTGGCTTG

CAGAGTCTTCAGGACCTCCCGGCCGGCATCCCCGACCTTGTCTTCCAGTATTCTCCAGCCAGTGGCTGCC

CTTCTCTGCTGCCACACACACACCCCAGTTCATTGGCTCGGGGTTCCCTCTATTGGGAACCCTGGTCCAG

CCTCTGCTTGCAGGGGAGTCACCTTCTAGCAGATCATCCCGAATGTCACAGCTCCTCAGGGATGTCTGCC

CAGACCACCCAGGCAAAGACTGCCAGCCAAACGCTGTCACCAGTTTTTTTAAAAAAGATTTGATTAATTA

ATTTATTTGAGAGAGAGAACAAGAGTGAGAGAGTGGCAGAGGGAGGGGGAGAGGGAGAAGCAGGTGAGCT

ACCTGTCCGGCTCACTGCAGCACTAGCAAAATGCCTCTCACCTGGTTGGGCTCACTGTGGTCAGCAAGTG

AATGGTGCCGAAGCCAGGAGCACCTTGTGAGGTGTCCCCAGGAGCCAGCAGGTCAGAGGGAAGGTCCCAG

TCTGGGCCCTGGCGCTGTACTAGGCTGCGCTGCCACCATCTCATGACATCTTCCCCAACTCCCGAGTTTG

TCAAGAGTTTTGCAAAAAAACACACCCACTGCAAACCAAAGTCCCTATTCATTACCTTTTTGTCTTGTAA

AGCACTGACAAGCCTACCGGCCCCACCCTCACCCCCAGCAGCTCTTCCCATTAGCATCTCCATGAACATT

CACCTCTACCTGGATTGAAGGCCTGCTTTCTTATGTCCTCAGAGTTTGTGAAATCCTTTCTTGTGGGTTA

TCTCATTTGTCCCAGAACCACCCTGGCTGCCCAACAGGTGAGGAAGCTGGTCTCAGAGAGGTGGAGGGAC

TCGCCCACCGCACACAGGCAAATGGATGAGCTTTTATTCGAAATGACCACTCGTGGGGTGCTTGGGTTGC

TCAGTGGTGGAGCACCTGCCTTTGGCTCAGGTTGTGAGCCAAAGGGTCCTGGGATCGAGTCCCGCACTGG

GGTCCCTGTAGGGAGACTGCTTTTCCCTCTGCCCGTGACTCTGCCGCTCTGTGTCTCTCATGAATAAATA

AAATTTTAAAAAAAGCTTTTGAGATTTTATGGAAGGGAGCTCAGAAGTCAAAGCCCTAGGTCTGACATCG

TATCATTACTTATATTTGCTGTGCAACTCTACCCATCCCAAGGGCTAGCCTTTTGGCTTCCTTACATGCA

GGAATTGAGTTTTGCTCATCTTTGTTTAGTGAAAAATACCTAGGATGGGGAGACATGAGCAGCTGTAGCA

GGGCATGGGCTCTGAGGCAGACAGGCTAGGCCTAAATCCCATGCCCACCACCTAATAGGTGGGGAGGTTA

CTTAACCTCTGTGCCTGGCACAGTAAGAGCTCAACAGATGTGTTTGTTTATAGGATTTTCCATTCTATTA

GTTTGATTATTATTTTAGACCCAGAACTTTGTAATTGAGTGGGTGGGGCTTCTGTAGTCCTGCCTCATTA

ATGCTAGTGCCCCTCTTTCAGGGATCTCTGTTCAGTAAATATGCCAGGAGGGGCTGTCCTGGGATGCTAG

GCCCGAGGTCCCAATCCCACTGGGAGGGAGACAGCCTCATAGACAAAGGAGGTCTCTCCCTCTCTTGGTC

CTTGGCAGCAAGTAAATTCTGTTTTTCTCAAAAAAGCAGGCACTATCACCAGGAAACCTTTTTGGAGCAT

GTACTTCCCATGGATGAGGATCAAGGGCCATTAAACTAAATTGTCAGAAGCTCAACCAGACCTTCCAAAT

GGCTGGGGAGGCAGGACTCATAGATATCCCTGGTGGGAAGGCCAAACTCAGCTGGGACCTCATCCAGTTG

CCCACCCCACCACCATCCTAACAATGGCTGTTGTTTGTAATCCGCACAGGGGCTCACTGCTCCGGTGGCT

TATCTCATTAATCCTCTCAAGAAACCATAGCAGTGGGTATCTTCAAGCTGAAGGAACTGAGGTTGAAGGA

ATTAACTCACTCATTCAGATTCAGAAAACTCCCGAGCCAGGGATTTGAATCTGGGCTGTTCAAAAGCCAC

TGTCCAAGCTCTAAATAATTATGCTCCTCTGGCTTCATCCAGAGTCTGTAAAAATAATAATAATAATAAT

ATTTGTTTGGTGCAAAAAGGTTTTTTTGGCTGAAAGCTATTTGGGAAAACACGATGATTCTTAAAAGCAG

CAAGGCAGTATCTGGTAAGACTCAACAACTTACTACTTTGCAGAATCACTTCTAGGTAAGACTCCCTTAG

AAGAGTTTTGTATGGAGGGAGCAGGAGTTCTGCGCAAATACGTTCATATCAGTGATGTTCTCCGTGACAG

GAAACATCACTATCCATCCGTAGGGATTGGCTACATGAAATACAAGAGTTTACTGCAGCAGTTTAAGGAA

ATGAATGTATCTGTGTGTACGTAACTAGATAGATATAGCTCAGATTGATTAAAAAAATACAATTCGAGGG

GGAAAAAAAAGAAAAACATGGTAACCTAAGTAGTTTATGTGAATTAAAAATATAAAATGAGTACAGTTGA

CCCTTGAATAATGTGGGGGTTGGGGGCATCGAACCCTGTGCTATAGAAAATTCATGAGTTGGGGCACCTG

AGTGGCTCAGTGGCTGAGCTTCTGTCTTGGGCTCAGGTCGTGATCCTGGGGTCCTGGAATCGAGTCCCGC

ATCAGGATCCCCACAGGGAGACTGCTTCTCCCTCTGCCTATGTCTCTGCCTCTCATGAATAAATACATTT

AAAAAGTCTTAAAAAAAAGGGATCCCTGGGTGGCGCAGCGGTTTGGTGCCTGCCTTTGGCCCAGGGCGCG

ATCCTGGAGACCCGGGATCAAATCCCACGTCGGGCTCCCTGCATAGAGCCTGCTTCTCCCTCTGCCTATG

TCTCTCTCTCTCTCTCTCTCTCTCTCTCTGTGACTACCATAAATAAATAAAAATTAAGAAAAAAAAAAGT

CTTAAAAAAAAAAAATCCAGGGACGCCTGGGTGGCTCAGTGGTTGAGTGTCTGCCTTTGGCCCAGGGCAT

GATCCTGGGGTGCCCAGATGGAGTCCCGCATGGAGCCTGCTTCTCTTTACCTGTGTCTCTGCCTCTCTCT

CTGTGTCTCTCATGAATAAATAAATAAAATCTTAAAAAAAAAAAAAATCCAGGGGATCCCTGGGTGGCGC

AGCGGTTTAGCGCCTGCCTTTGGCCCAGGGCACGATCCTGGAGACCCGGGATCGAATCCCACGTCAGGCT

CCCGGTGCATGGAGCCTGCTTCTCCCTCTGCCTATGTCTCTGCCTCTCTCTCTCTCTCTCTCTCTCTGTG

TGCCTATCATAAATAAATAAAATTTAAAAAAAAAAAAAAAAAAAAAAATCCAGGTGTTATCTCTTGACTG

ACCCAAAATTTAACAGCTTACTATTGAGAAGCCTTACCGATAACATAAGTAACTAAAACATATTATATAT

ATATATTATATACTACATTCTTTTTTTAAAAAAAGATTTTATTTATTTGAGAGGGAGAGAAGCAGACTAC

CCGCTGAGCAGAGAGCTTGATGCAGGGCTGGATCCCAGGACCCTAAGATAGTGACCTGAGCTAAAGGCAG

ACTGGCCACCTAGGTGCCCCCCATATACTATATTCTTAAAGTAAGCTAGAGATAATGCTATTAATATAAT

CACAAGGAAAAAAAGAAACATTCACAATACTGTTACTGTATTTATCCAAAAAACCCATGTATAAGTGGTC

CTACACAGTTCAATCCTGTGTTTTCTAAGGGTCAACTGAATTTATTTCTTACCAACATATATATGTGAAA

GTATGAAAGACAGATAAAAAGAATTAAAACTTACACTCATGAGACTGGGGGTACCATCAAAGCGACCTTA

TTTGATTAATGAAGAAAAATTTAAAAAGATGGTATTCATACATTATTTGTTTATTATAATATGAACATGA

CAAAATGTCAAGTGTTATAATTTCCTCTAGCTTCAAGAATAATCAACATTCTGCCTGTATTGTCTCCCCT

ATACTTCTAGTGGCTCCGCAGAAGCCCACAGATATTTTTGAGTTACAATCTATGTAAGTTGAAATGCACA

ATCTTAATTTTGCCATTTAGATAACAAAGTTTGACACATTTTGATATAACCTTGTCACCCAGCCCTACCA

AGCATAAGACACTGCTGCCATGTTTCCTTCTGAAAGTTAATATATTTTATGCTTAGATCTACTATCCATC

TCAAATTGTGTATAGTATGAGGTAGGGGTCAGAATCCACTCAGCTCTGCTTGTTTCAGCACTCTCTTCAG

ACTTTCCTTCCCCCATGGATTCATTTTGGAGCTTTTGTAGAAAACCATTTGATTACACTTATGTGTCTTT

TCCTGGGCTCTATTCAGTTCATGGTTGTTGTCTTATGGTAGCTTTACAGAATTCTTGAAGTCAGCTAGTT

TAAGACCTCCAGCTTTGTACTACACTTTCATTTTGGAATTGCTTGTCAGTTTCTACAAACAAAACCAGCT

AGGATTTTGATGGGGATCAAATGAAATCATCTTGGAGTACTGACATCTCAACACTATTGAGTGTTCCATC

TATGAATATGGTACTGTTACTTAAATCTTCCGGGCAGCCCGGGTGGCTCAGCGGTTTGGCACTGCCTTCG

GCCTGGGGTGTGGTTCTGGGGACCAGGGATCGAGTCCCACGCAGGCTCCCTGCGTGGGGCCTGCTTCTTC

CTCTGCCTGTGTCTCTGCCTCTCACTTTGTGTCTCTCATTAATAAACAAATAAAATAAAATAAAATAAAA

ATCTTCCTTAAATTCGCTCAGCAATATTTTGCAGTTTCTAACATAGAACTCTTGTCCGTCTTTCTTCAAT

TTATCCCTGGGTATGCCATGTTTTTGGATGCTTTGTAAATGCCACTGCTTTTATAATTCATTTTCCGACT

TCAGATTCAATGGTTGTTATTACATAAAAATACAATTTATGTATATTGCCTTTGTAACCTGTGACCTTGC

TATAATCCTTTATCAGTCATGATAGTTCTTCTTTTTAAGAGGTTTCATTTTTTAAGTAATCTCTGCATCC

AAGGTGGGCCTCAAATTTACAACTCTGAGATCAAGAGTCATGTATTCTACTGACTGAGCCAACCAGGTGC

CCCTTGTGAGTTTTTCAATGCTTCTTCTATTAGCCTGGTTAACACCTTCAGTATAATGTTGAAGGGGTGA

GAATCAAATCCTTTCCTAGTTCCTCATCTTAGAAAACAAGCATTCAGTCTGTCACTGTTACGTATTGATG

GTAACTTGATTGGGGGTGCTCTTTATCAGACTAAGTTTTCCCTTTCCTAGTTTGCTGGGAGGTTGGCTTT

TCTTTTCTTTCTTTTTTTAAAATCATGAATAGGTGTTGCGTGATGTCAACTGCTTTTTATGTATCTGTTG

AAATGATCATAGGATTTTTATCCTTCAATCTGTTTATGTGGCAAATTACACTGATTTTTTGAATGTTAAA

ACATTGCCGTCTAGAGATAAACTCCACTTGATTAGGATGTATTTATCCGTTTTATAGATAATCAGATTTA

CTTTGCTAAGATTTGTGAGATTTTTGCATATATACATATGTGTGTATATATACACACATATGTATATATG

CTGGCAGGAATATTGGTCTAAATGTTTTCTTGTAACATTCTCGTCTGGTTTTGATATGAGGATTCTCAGG

ATATCATGAAATTAGTTGGGAAGATGTTACCTCTTCTACATTCTGAAAGTTTTTGTAAGATTGTTATTGT

TTCTTCCTCAAGTGTCTGAAGAGAAGAAGAAATAGAATAGAAAATAGCATTAACACAGTAGACTTAAACC

CAAACCTTACAATAATTGCATTAAATTAAATAGACCAAGCACTCCAATTAAAAGTGATCCAGTGAGGGGG

GAAGGAGGGCTGTTTCAACAAGTGGCACTAGAAGAACTGGATATACATCTAGGAAAACCCCAAAAGAAAA

CAGAACCCCTACCTCACATCACGATAAAAATTACTCTTAATTAATTAGTTAATTAATTATGGCTCAAAGA

CCTAAATATAAAACCTAAAACCCTAAAGATTCTGGAAGAAAATATCCTAGAATATCTTCATGATCTTGGG

GTCAGCAAGGATTTTCTAAACACACAGAACACATTTGAAAGACAGAAGATAAATCTGCTGCTGCTCTGCC

TTAGAGGCTGACAGAGAGGCAAGAAACAGGTCAGGAGAAAATAGATACTGACAAAAGACTCCTATCCGGA

ATATATAAAGAACTGCATGCAGCTGAACGCAAGTTAAAATGGCCAATAAGCACGTGGAAACTGATCACAG

CTAAGAGAATTGCAACTGAAAACCACAATGATGCACCACATTAGAAAGTCTGACAAAACCAGATGTGAGG

AAAGACAGCTGTTGCTGGTGGGTACATAAAACAGAACAACCACTTTGAAATATACTAATTTTAAAAATCA

AATTTAGATCTCTCTCACCTAGCAAATTCCACTCCCAGACGGTTATAAAAAACAAAAACAAAACAAAACA

AAACACACACACACAAAACACCCAAAACCAACCCCATGAAAACATAGGACCACAAAGAGAATTGTACAAG

AATGTTAATAGCAGCTTTATTCAAAATAACCCCGAACGGTTAACAACCCAAGCGGTCACCGAAAGGAACA

TGGATTAAAAAAAACCTCACGGTTTATTCATCCAGTGAGATGTGCTAAATACAAAAATACAAAGGTGCAA

AAAACACTGATACACTACTGATACAGTAGTACTGGTTACACAGGAACAAGGATGGGTGTCACAGGTATTA

TGCTGCCGGCGAGAAGCCTACACAAAGGAGCACACACTATGTGGGTCTATTTACACGACGCTCTCTTCAA

CAGGCACAAGTAGTGACAGAACTCCAAGATGAGGGTCAGAGACCCCCAGAGGGGAGAGGCCCAAGAACCT

TCTCTGGGGCGATGACAATGTGTAAATTGTGTCATTTGACAGGCTGCTGGTTAGCAGCCCAAACCTGTCT

AATTGCAGTGAGTGTAAATGCTACCTCAATTTCAGAACAAGCCGCACACAAGCACCGCGTGCACTGGCCG

GTACCGAGCAGGTGCACAGGTAGGTGAGCGAGCGCAGGCTGGAAGCACCGCGCGCCCGGAGTGACTGACA

GGCGGGAGGCGGTATCCCGTCCCCTCGGCTCGGCTCGGCTCGGCTCGGCTCGGCGGGCCCCGCCCCCTCA

AGCCCCGCCCCTTCCGCCCTCACGACGTTCGCTCTGCGTCGGAACTCGGAATGGCTGAGTCCCGAGCCGG

ATGCCAGAGAAACCGTGTCTCGGCCCCACACAAACGGCGCATCGTCGCATTCTGCCATCGTTGTCATTCT

CAAATGTACTCGTGCTCGAAAATACCCGGGACTTGCCGTCGGCACGCTTCTGGAGACCTCGTCCCTCGCC

CGCCCAGATTGCAGGCGATGGGGTGGGGCGCACCATTCAAGGAGTGGGGGAAGGCTCGAGGTTTGCATCC

AAACAGACCCTCCCTCCCGGCCTGCCAAAAGTAGCAGCTAAGGAGTTCAGGGGAAAAAAAAAAAAAAAAA

AGAAAGAAAAGAAAAAATAAGAAAGAAAAAAGAAAAGAAAAGAAAAGAAAAGAAAGCCAGTAGAGGAGGC

GGCGCGCAGCCTCCCTGAGCACGTCAGTCTAAATGACACTTTCCCTCTCCTCGGGGGAACAGTGAACTGC

TCCGGAAAAAAAACCGGAAACGCTGCGGCAAGGGGCAGAAATGTAAATGTTCGACCAAACAATAACAGAG

CTCGCGGGCCTCCTGGACCCGGACCGTCCCGCCGGGCGTCGCGGGCAGACTGCTCGTGGAGCGCTACCCA

CGTCCACGCCAGGCAGTCTACTAGGATCGTCTCCATTTAGTGGAGCCAGGGGCCGCTGGGGAGATCCCTC

CGGGAAGGACAAAGGTCCGGAAGTTCTGGGACCTGAGCGGCTTGGGCTTCCTGGATCGACCCCCCCGAAT

GTGATCATGTCGGGACGGAGCCCGAGAGGACCCTCCAGGGATGCTGCGGGCTCGAAGATCTCGGGGGTCC

CCAAAATCCCGAGATCGGGGAAAAAACAATCTCGGATCCTGATGCTTTGTGCGTCTTATTTCGGGGTTCT

TCACACCCGGCACGGGGAGAGAAAGGGAGGGGAAGGGTGAGGAGGAACCCCTTCGGCCGGCTGCACGTCC

CCCTCCTATAAAGCTGAGTCCGGCTGCTCCCAACAGCCCCTTCATCTGCACCCAGGCCTCCCAGCGGTGC

ATTCCGATATTGGTGCCGCCAGCCAAACTTCCATTCCCCCGCGATCGCTGATCGCGTGCCCCACCCCCAG

ACCCCAATTCTCCCCAAGCTCAAAACACACAGAGCCGAGAGCCGGTGACTCAGACAGGACTCATCTGGCT

CAGTCAGGAGCTTACCCGAACCTCAGGGGAGCGTGTCACCGTCCTGGGAAGCACGCTCCGAGGCCGGACG

CCAGAGGATCCCGGAGCCCGGCGGCTACCTGCGCCCAGGATGGTGGCTCTGGACTTTTGAGAAGCTCAAA

ACT

>Canine TP53_UU_Cfam_GSD_1.0_NC_049226.1:32768467-32781775 Canis lupus familiaris isolate Mischka breed German Shepherd chromosome 5, alternate assembly, whole genome shotgun sequence

GGTAGTAAAGTTTTATTGTAAAATAAGAGATTGATATAAAAACGGGATATAAAAAGGGAAGAGGAGGAGG

GGTGGGGGTGATCAATGCATGAATGCAGATGTGCCTCACAGAGTGCAAAAGAAATTGATCCTGAGCAATT

GGGGGCGGGGGGGGGGGGGAAGCAGTAAAAGCCCTTGGTAGACAAAGATCCTACACCAGGGATGGGATTC

TGCAGGGTGTGAGATGGGAGTGAGATTTCATCTTAGGTGCAGGGGTCTCCCAAAAAGGCTGGACAGGATT

TGGCCAGGGCAGGGAATGAGCACCCACACAGGCACTAAGTCACAGACATGGGATAAAAGGTGGTTTTGGG

GTCAGACAGACATTATTTCATTCATCCTCACAAGCTCAGTGAGGTAGGTATGGTTAATTAGCAATTCATA

AAAATGGCCTGTGAAATTAGGAAAAATTCAGCCACTGGCTACAATGATAGAGAACTGGGCTCATGACTCA

GTGTCTTAGGGGCTATAAAATGCTCCCAACATTCAGGAACATTTTCCTACCTCTCCCATGGTTCTACACA

GTAAAAAACTTAAAAACCAGGCTGGAACGTCCTTTATCTCCCGACCTACCAACTGTGCAGGCCAACTTCT

TTAGTAGAGACTTACGGGAAAGGCAAATGGAAGAATCTAGGGTGTTTCTGAGGCACATCTGTACCATGCA

AAGTAACACACCGGAATAGGTGTGCTCAAGCTCCCAATTCCAAAAATGCCAGGGAAGGAGGACGAGAGTG

AGGGTGTGCGTGTTGGGGGAGGACAGGAAGCAGGGGAAGTCAGTCTGAGTCGGGCCCTTCTCTCTTGAAC

ATCAGTTTTTTATGGCGAGAGGTAGATTGCCCCTTCTTTGCCTTCAGGTGGCTGTTGGGGACACAAGGTG

GAGGGGAAGAGACGATATTAGCAAGTGGGAGCCTCGGGGCTCCTTGTCCTAGATCTAGGCTTGGAAGGGT

CCAAGGCTGGAGTAAGGGAATTTTTTTTTTTTTTTTTTTTTTGGAATGAGGTAATTTAAGTTAAAATGCT

GTAGACACATTTACTGAGCACCCAGGGAACTTCTGTGTGTTCGCTTCCTCTCCCCTCACCCCATCTGATG

AACAAGGAAACCGAGCCCCAGAAGGGGAGATGCAAGGCCACACGGATCTGCTCATCCTTGGCAGGGCTGG

GGGTCTGGCCCAATCCTGCTGTGTCCAGTATTTTCCCACACGGTTATCAGGTCCCTATTGGAGACCCCAC

TTGGCACTAGGGGTTGCTGGAGATGACAAACACCCATGCTTGTCCCTTAAGGGGTTTGGGGACCTCCCGA

GGCAGAAAAGGGAGTACAGGATCCAACTGGGGGTATGGTAGAAGCAGGTTTGCCCAGGGTGGGACCAACC

ATGAGGGGATCAGTTCCCTTCCACCTTGAAGGTAGGAGTAGAATGGAATCCTGTGGCTTTCCAACATATG

AAGGCAAGGGGGTGGGGCTGCAAGCCGGCCCAGGTCACTCACCTGGAGTGAGCCCTGCTTCCCCCTGGCT

CCTTTCCACTCTGGGCATCCTTCAGCTCCAAGGCTTCATTCAGATTCCTGAACATCTCATAGCGTTCACG

CCCACGGATCTGTGGCAGTGGGGGGGAGGAGGAGAAAGAATAAGAGGGGGAAGCCACAGTACCATTTAAC

TCAGACTAAGGTGTAAGTGCCAATCTGCAGCAAGGCACAGCCCGCAGCCACTGTCAAGGCACCGCTGCCC

CTTGATGGCAAGTGTGCTAATTGCAGGCGGAGCAGTCAAAAGAAAACTGTGCTCTTGGCCCTCAGCTTGA

AACAAATCTTCAATCCAAGAATATCAACTTCAAGAATCAGGGACAGGACCAGAGAAAAGGATGGAGAGAG

GAACAGGCCTAGCCTCACATGTGCTCCATTCACATTTTTTTCTTCCCTCATCTGATCCCGCTAAGCGGGG

GCAAGAGCCTGCCTTATCTGTTCCTCCCAGCCTTGGTACCTGAAGGGTGAAATATTCTCCATCTAGTGGC

TTCTTCTTTTGCGGGGGAGAGGAGCTGGTGCTGGGAGGCAGTGCTGGGAAAGAGAGGAGGAAAAGACAAG

AGAAGAGTATGTTTTGAGCAGAATTGCACCCTCTGTATCTGCCCCACATCCTCCTCTTTTTTTTTTTTTT

TTCAAGATTATATTTATTTATTCATGAGACATACACACAAAGGCAAAAAAATAGGCAGAGGGAGAGGCAG

GCTCCCTACAGGGACCCTGACACGGGACTCGATCCCAGGACCCTGGGATCATGCCTGATGCTCAACCCCT

GAGCCACCCAGGTGTCCCTCCTTCACCTCCTCTTGTCTTGCTTGCTTACCTCGCTTGGTACTCCCGGGGG

GTGGCTCAGGACAAGGCTCCCCCTTCTTGTGGAAATTCTCCTCCTCAGTCCGGCGGTCTCTCCCGGGACA

GGCACAAACGCGTACCTCAAAGCTGTTGCGTCCCAGCACGTTTCCACTACCCAGGTGAGAAGAGAGAAGC

AGGGGGCCGTGAGGGTGGTGGGCCCCACTGACTCCTAGCCACCCTCATCCTACGTAGAGCCAAAGCCTAC

AGACTATTGAGAAGCCACAGGTCAGGGAGGTCCCAAAGCCAGAGGAAAGAAAACCAGGGGAGGCGGATGG

CAGAGGAGATTCCACATTGGGACAGGAATGGATGGGAAGGATGGAGGGGGGTACAGTAAGGAAGTGGCCA

GGAGCAGGGTGGCAGGCAGGTCCCTACCTGGAGTCTTCCAGGGTGATGATAGTGAGGATGGGCCGCCGGT

TCATGCCTCCCATGCAGGAACTGTTACACATGTAGTTGTAGTGGATGGTGGTATAGTCAGAGCCAACCTG

AGGTAGAAGGTAGGCCCAGGGTCGGGCCACTCAGCCCTGGGCAGGCCTGTAGTCTATCAGCCTCAGTCTT

TCCCAACCGCAGAATGGGATGACCCACCTGCCTCTGCACTCCTCACAGGGCTGCGGGGAGGTTGAAGGAG

GAGAAAGCCCCCTCAACTCCCGCCTCACAAATCCCTCCAGCCCCCTCCTCCCAGAGACCCCTCAGATGCC

AAACCAGACCTCGGGTGGCTCATAAGGCACCACCACACTGTGTCGAAAAGTGTTTCTGTCGTCCAGGTAC

TTGGCCCGCAAATTTCCTTCCACTCGGATGAGATGCTGAGGAGGGGCAAGACCTAAGAGCCATCGGGGAG

GAATCAGGGCTTTGGGCCCTCTGGGGAGCAGCCTTGTCCCATCTGTAGCCCCCGATGGCTTACCGTCACT

ACTGTCAGAGCAGCGTTCATGGTGGGGGCAGCGCCGCACAACCTCGGTCACGAACTCCGACTTCTTATAG

ATGGCCATAGCGCGGACGCAGGTATTGGGTGGGGGTGGGGAGCTGACCCACAGCTGCACGGGGCAGGTCT

TCGCCAGCTGGCAAAACAACTTGTTGAGGAGAGGGGAGTACTGTAAGAAAAGGACAGATGGACAGGTCAG

GGCATGTGTGCCCGGGGTGGGTGGTGAGAAGTAGAGGGGTCGGGGTCTATTCCAGAAGCTTACTTTATTT

TAGACACACAGAGAGAGGCAGAGACACAGGCAGAGGGAGAAGTGGTATGGGATTCGATCCCAGGACCTGG

GGCTCACACCCTGAACCAAAGGCAGACGCTCAACTGCTGAGCTGCCCAGGCATCTCCCAGAAGCTTACTT

TAAAAGCCAAGGAATGAACAAGTGAAGGAAAATGAAGAAGAGTCCCTTTGCCCCATGACAGGAAAGGTGA

AAGCCCAAGGTCAAGGGGGGAAATGTCAACTTTGACACAGAGCTGAGGCCAAAGGTCACAGAATACATAG

GTAATGCAGGAAGACTGACAGTGCGGACCGAGAGGAGTCTCAAAATCCCAAACAGAGAAACCCAGGGGCC

ACAGCCAGGCCCCGGCCAGCCCCATGGAAACCCGCCTCGCAGGGCCACTGACCGTCCAAGTAACAGACTT

GGCTGTCCCGGAATGCAGGAACCCCAAACGGAACCCATAGGTGCCAGGGTAGGTCTTCGGGGAAGGGACA

GAGGATGATAGGGGCCAGGAGGGGGCCGGTCCAGGGGCTGTGGGGGCAGAAGTGGCTGGCATCCTGGGAG

CATCATCTGAGTCTTCGTCTAGCCAGTTCACGACGCTCTCTGGGAGCAGCAGCTCATCCACTGCTGGGCA

CAGCTCCGAAGACTGTAGGGGGCGAGACCAGAGTCAAGGGTCAGGTCCCTAGGTCCCCAGCTCCCAACCC

CCGCCCTGGACCTCCCCACACCCAGTCCTTACCAGAACATTGTTTTCAGGAAGCCTGGAAGATGAGAGCA

GAAAGTCAGTGCTGGTTTTGCTTCCTCTGGGCTTCTACTTGGGGGGGCGGGGGGGGGGGGTGCTGGGGGT

TGGGATTGGGGTGGGTGGGCAGGGGTCCCAGAGGGCTGGTGGGGAGCCTGGCCTGCCCTCCACTTACAGG

TTCCACAATTCTGAAAATGTCTCCTGGCTCAGAGGGGGGTCGATATTGAGCTCTGACTGTGGCTCTTGCA

TCGCAGCTCCTGGGAAGGGAGTGTGGGTGCTGTGGAGGGCAAGGCGAGGGGTCAGCGGGAGACAAGCCCG

GGCCCGGGTCCCTCAGCCCCGAGGTGGGAGGGACCCTTCCAGCAACATTATACCAGGGCCTGGGATGCCT

TGGCTCCCCCACCTGGGCCTCCCCAGGAGCAGGGCTGCTCCTGCCCATTCCACCCCTGGGCGGCCTACCC

CCACACATCCTCAAGGTACAAAGATAACCAGAAAGGGGATTCTAGCCCTCTCATTACAGAGTCCACAAGC

CTTCCTGGTTATAAGGATGAAGAACGTCAGCCCACACCCTCCACCTGGACGAAGATACCTGCATGGACCC

CCTCATTCCCCACCTGATCCCCTTCATCACGAAGCTTCCTAAGGATCTGCTATCTCCATTCCACCCTTGG

CTTGCAGAGTCTTCAGGACCTCCCGGCCGGCATCCCCGACCTTGTCTTCCAGTATTCTCCAGCCAGTGGC

TGCCCTTCTCTGCTGCCACACACACACCCCAGTTCATTGGCTCGGGGTTCCCTCTATTGGGAACCCTGGT

CCAGCCTCTGCTTGCAGGGGAGTCACCTTCTAGCAGATCATCCCGAATGTCACAGCTCCTCAGGGATGTC

TGCCCAGACCACCCAGGCAAAGACTGCCAGCCAAACGCTGTCACCAGTTTTTTTAAAAAAGATTTGATTA

ATTAATTTATTTGAGAGAGAGAACAAGAGTGAGAGAGTGGCAGAGGGAGGGGGAGAGGGAGAAGCAGGTG

AGCTACCTGTCCGGCTCACTGCAGCACTAGCAAAATGCCTCTCACCTGGTTGGGCTCACTGTGGTCAGCA

AGTGAATGGTGCCGAAGCCAGGAGCACCTTGTGAGGTGTCCCCAGGAGCCAGCAGGTCAGAGGGAAGGTC

CCAGTCTGGGCCCTGGCGCTGTACTAGGCTGCGCTGCCACCATCTCATGACATCTTCCCCAACTCCCGAG

TTTGTCAAGAGTTTTGCAAAAAAACACACCCACTGCAAACCAAAGTCCCTATTCATTACCTTTTTGTCTT

GTAAAGCACTGACAAGCCTACCGGCCCCACCCTCACCCCCAGCAGCTCTTCCCATTAGCATCTCCATGAA

CATTCACCTCTACCTGGATTGAAGGCCTGCTTTCTTATGTCCTCAGAGTTTGTGAAATCCTTTCTTGTGG

GTTATCTCATTTGTCCCAGAACCACCCTGGCTGCCCAACAGGTGAGGAAGCTGGTCTCAGAGAGGTGGAG

GGACTCGCCCACCGCACACAGGCAAATGGATGAGCTTTTATTCGAAATGACCACTCGTGGGGTGCTTGGG

TTGCTCAGTGGTGGAGCACCTGCCTTTGGCTCAGGTTGTGAGCCAAAGGGTCCTGGGATCGAGTCCCGCA

CTGGGGTCCCTGTAGGGAGACTGCTTTTCCCTCTGCCCGTGACTCTGCCGCTCTGTGTCTCTCATGAATA

AATAAAATTTTAAAAAAAGCTTTTGAGATTTTATGGAAGGGAGCTCAGAAGTCAAAGCCCTAGGTCTGAC

ATCGTATCATTACTTATATTTGCTGTGCAACTCTACCCATCCCAAGGGCTAGCCTTTTGGCTTCCTTACA

TGCAGGAATTGAGTTTTGCTCATCTTTGTTTAGTGAAAAATACCTAGGATGGGGAGACATGAGCAGCTGT

AGCAGGGCATGGGCTCTGAGGCAGACAGGCTAGGCCTAAATCCCATGCCCACCACCTAATAGGTGGGGAG

GTTACTTAACCTCTGTGCCTGGCACAGTAAGAGCTCAACAGATGTGTTTGTTTATAGGATTTTCCATTCT

ATTAGTTTGATTATTATTTTAGACCCAGAACTTTGTAATTGAGTGGGTGGGGCTTCTGTAGTCCTGCCTC

ATTAATGCTAGTGCCCCTCTTTCAGGGATCTCTGTTCAGTAAATATGCCAGGAGGGGCTGTCCTGGGATG

CTAGGCCCGAGGTCCCAATCCCACTGGGAGGGAGACAGCCTCATAGACAAAGGAGGTCTCTCCCTCTCTT

GGTCCTTGGCAGCAAGGTAATTCTGTTTTTCTCAAAAAAGCAGGCACTATCACCAGGAAACCTTTTTGGA

GCATGTACTTCCCATGGATGAGGATCAAGGGCCATTAAACTAAATTGTCAGAAGCTCAACCAGACCTTCC

AAATGGCTGGGGAGGCAGGACTCATAGATATCCCTGGTGGGAAGGCCAAACTCAGCTGGGACCTCATCCA

GTTGCCCACCCCACCACCATCCTAACAATGGCTGTTGTTTGTAATCCGCACAGGGGCTCACTGCTCCGGT

GGCTTATCTCATTAATCCTCTCAAGAAACCATAGCAGTGGGTATCTTCAAGCTGAAGGAACTGAGGTTGA

AGGAATTAACTCACTCATTCAGATTCAGAAAACTCCCGAGCCAGGGATTTGAATCTGGGCTGTTCAAAAG

CCACTGTCCAAGCTCTAAATAATTATGCTCCTCTGGCTTCATCCAGAGTCTGTAAAAATAATAATAATAA

TAATATTTGTTTGGTGCAAAAAGGTTTTTTTGGCTGAAAGCTATTTGGGAAAACACGATGATTCTTAAAA

GCAGCAAGGCAGTATCTGGTAAGACTCAACAACTTACTACTTTGCAGAATCACTTCTAGGTAAGACTCCC

TTAGAAGAGTTTTGTATGGAGGGAGCAGGAGTTCTGCGCAAATACGTTCATATCAGTGATGTTCTCCGTG

ACAGGAAACATCACTATCCATCCGTAGGGATTGGCTACATGAAATACAAGAGTTTACTGCAGCAGTTTAA

GGAAATGAATGTATCTGTGTGTACGTAACTAGATAGATATAGCTCAGATTGATTAAAAAAATACAATTCG

AGGGGGAAAAAAAAGAAAAACATGGTAACCTAAGTAGTTTATGTGAATTAAAAATATAAAATGAGTACAG

TTGACCCTTGAATAATGTGGGGGTTGGGGGCATCGAACCCTGTGCTATAGAAAATTCATGAGTTGGGGCA

CCTGAGTGGCTCAGTGGCTGAGCTTCTGTCTTGGGCTCAGGTCGTGATCCTGGGGTCCTGGAATCGAGTC

CCGCATCAGGATCCCCACAGGGAGACTGCTTCTCCCTCTGCCTATGTCTCTGCCTCTCATGAATAAATAC

ATTTAAAAAGTCTTAAAAAAAAGGGATCCCTGGGTGGCGCAGCGGTTTGGTGCCTGCCTTTGGCCCAGGG

CGCGATCCTGGAGACCCGGGATCAAATCCCACGTCGGGCTCCCTGCATAGAGCCTGCTTCTCCCTCTGCC

TATGTCTCTCTCTCTCTCTCTCTCTCTCTCTCTCTGTGACTACCATAAATAAATAAAAATTAAGAAAAAA

AAAAGTCTTAAAAAAAAAAAATCCAGGGACGCCTGGGTGGCTCAGTGGTTGAGTGTCTGCCTTTGGCCCA

GGGCATGATCCTGGGGTGCCCAGATGGAGTCCCGCATGGAGCCTGCTTCTCTTTACCTGTGTCTCTGCCT

CTCTCTCTGTGTCTCTCATGAATAAATAAATAAAATCTTAAAAAAAAAAAAAATCCAGGTGTTATCTCTT

GACTGACCCAAAATTTAACAGCTTACTATTGAGAAGCCTTACCGATAACATAAGTAACTAAAACATATTA

TATATATATATTATATACTACATTCTTTTTTTAAAAAAAGATTTTATTTATTTGAGAGGGAGAGAAGCAG

ACTACCCGCTGAGCAGAGAGCTTGATGCAGGGCTGGATCCCAGGACCCTAAGATAGTGACCTGAGCTAAA

GGCAGACTGGCCACCTAGGTGCCCCCCATATACTATATTCTTAAAGTAAGCTAGAGATAATGCTATTAAT

ATAATCACAAGGAAAAAAAGAAACATTCACAATACTGTTACTGTATTTATCCAAAAAACCCATGTATAAG

TGGTCCTACACAGTTCAATCCTGTGTTTTCTAAGGGTCAACTGAATTTATTTCTTACCAACATATATATG

TGAAAGTATGAAAGACAGATAAAAAGAATTAAAACTTACACTCATGAGACTGGGGGTACCATCAAAGCGA

CCTTATTTGATTAATGAAGAAAAATTTAAAAAGATGGTATTCATACATTATTTGTTTATTATAATATGAA

CATGACAAAATGTCAAGTGTTATAATTTCCTCTAGCTTCAAGAATAATCAACATTCTGCCTGTATTGTCT

CCCCTATACTTCTAGTGGCTCCGCAGAAGCCCACAGATATTTTTGAGTTACAATCTATGTAAGTTGAAAT

GCACAATCTTAATTTTGCCATTTAGATAACAAAGTTTGACACATTTTGATATAACCTTGTCACCCAGCCC

TACCAAGCATAAGACACTGCTGCCATGTTTCCTTCTGAAAGTTAATATATTTTATGCTTAGATCTACTAT

CCATCTCAAATTGTGTATAGTATGAGGTAGGGGTCAGAATCCACTCAGCTCTGCTTGTTTCAGCACTCTC

TTCAGACTTTCCTTCCCCCATGGATTCATTTTGGAGCTTTTGTAGAAAACCATTTGATTACACTTATGTG

TCTTTTCCTGGGCTCTATTCAGTTCATGGTTGTTGTCTTATGGTAGCTTTACAGAATTCTTGAAGTCAGC

TAGTTTAAGACCTCCAGCTTTGTACTACACTTTCATTTTGGAATTGCTTGTCAGTTTCTACAAACAAAAC

CAGCTAGGATTTTGATGGGGATCAAATGAAATCATCTTGGAGTACTGACATCTCAACACTATTGAGTGTT

CCATCTATGAATATGGTACTGTTACTTAAATCTTCCGGGCAGCCCGGGTGGCTCAGCGGTTTGGCACTGC

CTTCGGCCTGGGGTGTGGTTCTGGGGACCAGGGATCGAGTCCCACGCAGGCTCCCTGCGTGGGGCCTGCT

TCTTCCTCTGCCTGTGTCTCTGCCTCTCACTTTGTGTCTCTCATTAATAAACAAATAAAATAAAATAAAA

TAAAAATCTTCCTTAAATTCGCTCAGCAATATTTTGCAGTTTCTAACATAGAACTCTTGTCCGTCTTTCT

TCAATTTATCCCTGGGTATGCCATGTTTTTGGATGCTTTGTAAATGCCACTGCTTTTATAATTCATTTTC

CGACTTCAGATTCAATGGTTGTTATTACATAAAAATACAATTTATGTATATTGCCTTTGTAACCTGTGAC

CTTGCTATAATCCTTTATCAGTCATGATAGTTCTTCTTTTTAAGAGGTTTCATTTTTTAAGTAATCTCTG

CATCCAAGGTGGGCCTCAAATTTACAACTCTGAGATCAAGAGTCATGTATTCTACTGACTGAGCCAACCA

GGTGCCCCTTGTGAGTTTTTCAATGCTTCTTCTATTAGCCTGGTTAACACCTTCAGTATAATGTTGAAGG

GGTGAGAATCAAATCCTTTCCTAGTTCCTCATCTTAGAAAACAAGCATTCAGTCTGTCACTGTTACGTAT

TGATGGTAACTTGATTGGGGGTGCTCTTTATCAGACTAAGTTTTCCCTTTCCTAGTTTGCTGGGAGGTTG

GCTTTTCTTTTCTTTCTTTTTTTAAAATCATGAATAGGTGTTGCGTGATGTCAACTGCTTTTTATGTATC

TGTTGAAATGATCATAGGATTTTTATCCTTCAATCTGTTTATGTGGCAAATTACACTGATTTTTTGAATG

TTAAAACATTGCCGTCTAGAGATAAACTCCACTTGATTAGGATGTATTTATCCGTTTTATAGATAATCAG

ATTTACTTTGCTAAGATTTGTGAGATTTTTGCATATATACATATGTGTGTATATATACACACATATGTAT

ATATGCTGGCAGGAATATTGGTCTAAATGTTTTCTTGTAACATTCTCGTCTGGTTTTGATATGAGGATTC

TCAGGATATCATGAAATTAGTTGGGAAGATGTTACCTCTTCTACATTCTGAAAGTTTTTGTAAGATTGTT

ATTGTTTCTTCCTCAAGTGTCTGAAGAGAAGAAGAAATAGAATAGAAAATAGCATTAACACAGTAGACTT

AAACCCAAACCTTACAATAATTGCATTAAATTAAATAGACCAAGCACTCCAATTAAAAGTGATCCAGTGA

GGGGGGAAGGAGGGCTGTTTCAACAAGTGGCACTAGAAGAACTGGATATACATCTAGGAAAACCCCAAAA

GAAAACAGAACCCCTACCTCACATCACGATAAAAATTACTCTTAATTAATTAGTTAATTAATTATGGCTC

AAAGACCTAAATATAAAACCTAAAACCCTAAAGATTCTGGAAGAAAATATCCTAGAATATCTTCATGATC

TTGGGGTCAGCAAGGATTTTCTAAACACACAGAACACATTTGAAAGACAGAAGATAAATCTGCTGCTGCT

CTGCCTTAGAGGCTGACAGAGAGGCAAGAAACAGGTCAGGAGAAAATAGATACTGACAAAAGACTCCTAT

CCGGAATATATAAAGAACTGCATGCAGCTGAACGCAAGTTAAAATGGCCAATAAGCACGTGGAAACTGAT

CACAGCTAAGAGAATTGCAACTGAAAACCACAATGATGCACCACATTAGAAAGTCTGACAAAACCAGATG

TGAGGAAAGACAGCTGTTGCTGGTGGGTACATAAAACAGAACAACCACTTTGAAATATACTAATTTTAAA

AATCAAATTTAGATCTCTCTCACCTAGCAAATTCCACTCCCAGACGGTTATAAAAAACAAAAACAAAACA

AAACAAAACACACACACACAAAACACCCAAAACCAACCCCATGAAAACATAGGACCACAAAGAGAATTGT

ACAAGAATGTTAATAGCAGCTTTATTCAAAATAACCCCGAACGGTTAACAACCCAAGCGGTCACCGAAAG

GAACATGGATTAAAAAAAACCTCACGGTTTATTCATCCAGTGAGATGTGCTAAATACAAAAATACAAAGG

TGCAAAAAACACTGATACACTACTGATACAGTAGTACTGGTTACACAGGAACAAGGATGGGTGTCACAGG

TATTATGCTGCCGGCGAGAAGCCTACACAAAGGAGCACACACTATGTGGGTCTATTTACACGACGCTCTC

TTCAACAGGCACAAGTAGTGACAGAACTCCAAGATGAGGGTCAGAGACCCCCAGAGGGGAGAGGCCCAAG

AACCTTCTCTGGGGCGATGACAATGTGTAAATTGTGTCATTTGACAGGCTGCTGGTTAGCAGCCCAAACC

TGTCTAATTGCAGTGAGTGTAAATGCTACCTCAATTTCAGAACAAGCCGCACACAAGCACCGCGTGCACT

GGCCGGTACCGAGCAGGTGCACAGGTAGGTGAGCGAGCGCAGGCTGGAAGCACCGCGCGCCCGGAGTGAC

TGACAGGCGGGAGGCGGTATCCCGTCCCCTCGGCTCGGCTCGGCTCGGCTCGGCTCGGCGGGCCCCGCCC

CCTCAAGCCCCGCCCCTTCCGCCCTCACGACGTTCGCTCTGCGTCGGAACTCGGAATGGCTGAGTCCCGA

GCCGGATGCCAGAGAAACCGTGTCTCGGCCCCACACAAACGGCGCATCGTCGCATTCTGCCATCGTTGTC

ATTCTCAAATGTACTCGTGCTCGAAAATACCCGGGACTTGCCGTCGGCACGCTTCTGGAGACCTCGTCCC

TCGCCCGCCCAGATTGCAGGCGATGGGGTGGGGCGCACCATTCAAGGAGTGGGGGAAGGCTCGAGGTTTG

CATCCAAACAGACCCTCCCTCCCGGCCTGCCAAAAGTAGCAGCTAAGGAGTTCAGGGGAAAAAAAAAAAA

AAAAAAAGAAAGAAAAGAAAAAATAAGAAAGAAAAAAGAAAAGAAAAGAAAAGAAAAGAAAGCCAGTAGA

GGAGGCGGCGCGCAGCCTCCCTGAGCACGTCAGTCTAAATGACACTTTCCCTCTCCTCGGGGGAACAGTG

AACTGCTCCGGAAAAAAAACCGGAAACGCTGCGGCAAGGGGCAGAAATGTAAATGTTCGACCAAACAATA

ACAGAGCTCGCGGGCCTCCTGGACCCGGACCGTCCCGCCGGGCGTCGCGGGCAGACTGCTCGTGGAGCGC

TACCCACGTCCACGCCAGGCAGTCTACTAGGATCGTCTCCATTTAGTGGAGCCAGGGGCCGCTGGGGAGA

TCCCTCCGGGAAGGACAAAGGTCCGGAAGTTCTGGGACCTGAGCGGCTTGGGCTTCCTGGATCGACCCCC

CCGAATGTGATCATGTCGGGACGGAGCCCGAGAGGACCCTCCAGGGATGCTGCGGGCTCGAAGATCTCGG

GGGTCCCCAAAATCCCGAGATCGGGGAAAAAACAATCTCGGATCCTGATGCTTTGTGCGTCTTATTTCGG

GGTTCTTCACACCCGGCACGGGGAGAGAAAGGGAGGGGAAGGGTGAGGAGGAACCCCTTCGGCCGGCTGC

ACGTCCCCCTCCTATAAAGCTGAGTCCGGCTGCTCCCAACAGCCCCTTCATCTGCACCCAGGCCTCCCAG

CGGTGCATTCCGATATTGGTGCCGCCAGCCAAACTTCCATTCCCCCGCGATCGCTGATCGCGTGCCCCAC

CCCCAGACCCCAATTCTCCCCAAGCTCAAAACACACAGAGCCGAGAGCCGGTGACTCAGACAGGACTCAT

CTGGCTCAGTCAGGAGCTTACCCGAACCTCAGGGGAGCGTGTCACCGTCCTGGGAAGCACGCTCCGAGGC

CGGACGCCAGAGGATCCCGGAGCCCGGCGGCTACCTGCGCCCAGGATGGTGGCTCTGGACTTTTGAGAAG

CTCAAAACT
