# Supplementary material for: Primer-walking sequencing identifies predominantly noncoding TP53 variants in canine cancers
Source: J Vet Sci. 2026 May 27;27(4):e42. doi: 10.4142/jvs.25325 (PMC13454805; doi:10.4142/jvs.25325)
Supplement: Supplementary file 2 [file jvs-27-e42-s002.doc]

**Supplementary Data 2.** Canis lupus familiaris_NCBI_ensembl_TP53_reference gene sequences

>Canine TP53_ROS_Cfam_1.0_NC_051809.1:32665395-32678721 Canis lupus familiaris isolate SID07034 breed Labrador retriever chromosome 5, whole genome shotgun sequence

GGTAGTAAAGTTTTATTGTAAAATAAGAGATTGATATAAAAACGGGATATAAAAAGGGAAGAGGAGGAGG

GGTGGGGGTGATCAATGCATGAATGCAGATGTGCCTCACAGAGTGCAAAAGAAATTGATCCTGAGCAATT

GGGGGCGGGGGGGGGGGGGAAGCAGTAAAAGCCCTTGGTAGACAAAGATCCTACACCAGGGATGGGATTC

TGCAGGGTGTGAGATGGGAGTGAGATTTCATCTTAGGTGCAGGGGTCTCCCAAAAAGGCTGGACAGGATT

TGGCCAGGGCAGGGAATGAGCACCCACACAGGCACTAAGTCACAGACATGGGATAAAAGGTGGTTTTGGG

GTCAGACAGACATTATTTCATTCATCCTCACAAGCTCAGTGAGGTAGGTATGGTTAATTAGCAATTCATA

AAAATGGCCTGTGAAATTAGGAAAAATTCAGCCACTGGCTACAATGATAGAGAACTGGGCTCATGACTCA

GTGTCTTAGGGGCTATAAAATGCTCCCAACATTCAGGAACATTTTCCTACCTCTCCCATGGTTCTACACA

GTAAAAAACTTAAAAACCAGGCTGGAACGTCCTTTATCTCCCGACCTACCAACTGTGCAGGCCAACTTCT

TTAGTAGAGACTTACGGGAAAGGCAAATGGAAGAATCTAGGGTGTTTCTGAGGCACATCTGTACCATGCA

AAGTAACACACCGGAATAGGTGTGCTCAAGCTCCCAATTCCAAAAATGCCAGGGAAGGAGGACGAGAGTG

AGGGTGTGCGTGTTGGGGGAGGACAGGAAGCAGGGGAAGTCAGTCTGAGTCGGGCCCTTCTCTCTTGAAC

ATCAGTTTTTTATGGCGAGAGGTAGATTGCCCCTTCTTTGCCTTCAGGTGGCTGTTGGGGACACAAGGTG

GAGGGGAAGAGACGATATTAGCAAGTGGGAGCCTCGGGGCTCCTTGTCCTAGATCTAGGCTTGGAAGGGT

CCAAGGCTGGAGTAAGGGAATTTTTTTTTTTTTTTTTTTTTTGGAATGAGGTAATTTAAGTTAAAATGCT

GTAGACACATTTACTGAGCACCCAGGGAACTTCTGTGTGTTCGCTTCCTCTCCCCTCACCCCATCTGATG

AACAAGGAAACCGAGCCCCAGAAGGGGAGATGCAAGGCCACACGGATCTGCTCATCCTTGGCAGGGCTGG

GGGTCTGGCCCAATCCTGCTGTGTCCAGTATTTTCCCACACGGTTATCAGGTCCCTATTGGAGACCCCAC

TTGGCACTAGGGGTTGCTGGAGATGACAAACACCCATGCTTGTCCCTTAAGGGGTTTGGGGACCTCCCGA

GGCAGAAAAGGGAGTACAGGATCCAACTGGGGGTATGGTAGAAGCAGGTTTGCCCAGGGTGGGACCAACC

ATGAGGGGATCAGTTCCCTTCCACCTTGAAGGTAGGAGTAGAATGGAATCCTGTGGCTTTCCAACATATG

AAGGCAAGGGGGTGGGGCTGCAAGCCGGCCCAGGTCACTCACCTGGAGTGAGCCCTGCTTCCCCCTGGCT

CCTTTCCACTCTGGGCATCCTTCAGCTCCAAGGCTTCATTCAGATTCCTGAACATCTCATAGCGTTCACG

CCCACGGATCTGTGGCAGTGGGGGGGAGGAGGAGAAAGAATAAGAGGGGGAAGCCACAGTACCATTTAAC

TCAGACTAAGGTGTAAGTGCCAATCTGCAGCAAGGCACAGCCCGCAGCCACTGTCAAGGCACCGCTGCCC

CTTGATGGCAAGTGTGCTAATTGCAGGCGGAGCAGTCAAAAGAAAACTGTGCTCTTGGCCCTCAGCTTGA

AACAAATCTTCAATCCAAGAATATCAACTTCAAGAATCAGGGACAGGACCAGAGAAAAGGATGGAGAGAG

GAACAGGCCTAGCCTCACATGTGCTCCATTCACATTTTTTTCTTCCCTCATCTGATCCCGCTAAGCGGGG

GCAAGAGCCTGCCTTATCTGTTCCTCCCAGCCTTGGTACCTGAAGGGTGAAATATTCTCCATCTAGTGGC

TTCTTCTTTTGCGGGGGAGAGGAGCTGGTGCTGGGAGGCAGTGCTGGGAAAGAGAGGAGGAAAAGACAAG

AGAAGAGTATGTTTTGAGCAGAATTGCACCCTCTGTATCTGCCCCACATCCTCCTCTTTTTTTTTTTTTT

TTCAAGATTATATTTATTTATTCATGAGACATACACACAAAGGCAAAAAAATAGGCAGAGGGAGAGGCAG

GCTCCCTACAGGGACCCTGACACGGGACTCGATCCCAGGACCCTGGGATCATGCCTGATGCTCAACCCCT

GAGCCACCCAGGTGTCCCTCCTTCACCTCCTCTTGTCTTGCTTGCTTACCTCGCTTGGTACTCCCGGGGG

GTGGCTCAGGACAAGGCTCCCCCTTCTTGTGGAAATTCTCCTCCTCAGTCCGGCGGTCTCTCCCGGGACA

GGCACAAACGCGTACCTCAAAGCTGTTGCGTCCCAGCACGTTTCCACTACCCAGGTGAGAAGAGAGAAGC

AGGGGGCCGTGAGGGTGGTGGGCCCCACTGACTCCTAGCCACCCTCATCCTACGTAGAGCCAAAGCCTAC

AGACTATTGAGAAGCCACAGGTCAGGGAGGTCCCAAAGCCAGAGGAAAGAAAACCAGGGGAGGCGGATGG

CAGAGGAGATTCCACATTGGGACAGGAATGGATGGGAAGGATGGAGGGGGGTACAGTAAGGAAGTGGCCA

GGAGCAGGGTGGCAGGCAGGTCCCTACCTGGAGTCTTCCAGGGTGATGATAGTGAGGATGGGCCGCCGGT

TCATGCCTCCCATGCAGGAACTGTTACACATGTAGTTGTAGTGGATGGTGGTATAGTCAGAGCCAACCTG

AGGTAGAAGGTAGGCCCAGGGTCGGGCCACTCAGCCCTGGGCAGGCCTGTAGTCTATCAGCCTCAGTCTT

TCCCAACCGCAGAATGGGATGACCCACCTGCCTCTGCACTCCTCACAGGGCTGCGGGGAGGTTGAAGGAG

GAGAAAGCCCCCTCAACTCCCGCCTCACAAATCCCTCCAGCCCCCTCCTCCCAGAGACCCCTCAGATGCC

AAACCAGACCTCGGGTGGCTCATAAGGCACCACCACACTGTGTCGAAAAGTGTTTCTGTCGTCCAGGTAC

TTGGCCCGCAAATTTCCTTCCACTCGGATGAGATGCTGAGGAGGGGCAAGACCTAAGAGCCATCGGGGAG

GAATCAGGGCTTTGGGCCCTCTGGGGAGCAGCCTTGTCCCATCTGTAGCCCCCGATGGCTTACCGTCACT

ACTGTCAGAGCAGCGTTCATGGTGGGGGCAGCGCCGCACAACCTCGGTCACGAACTCCGACTTCTTATAG

ATGGCCATAGCGCGGACGCAGGTATTGGGTGGGGGTGGGGAGCTGACCCACAGCTGCACGGGGCAGGTCT

TCGCCAGCTGGCAAAACAACTTGTTGAGGAGAGGGGAGTACTGTAAGAAAAGGACAGATGGACAGGTCAG

GGCATGTGTGCCCGGGGTGGGTGGTGAGAAGTAGAGGGGTCGGGGTCTATTCCAGAAGCTTACTTTATTT

TAGACACACAGAGAGAGGCAGAGACACAGGCAGAGGGAGAAGTGGTATGGGATTCGATCCCAGGACCTGG

GGCTCACACCCTGAACCAAAGGCAGACGCTCAACTGCTGAGCTGCCCAGGCATCTCCCAGAAGCTTACTT

TAAAAGCCAAGGAATGAACAAGTGAAGGAAAATGAAGAAGAGTCCCTTTGCCCCATGACAGGAAAGGTGA

AAGCCCAAGGTCAAGGGGGGAAATGTCAACTTTGACACAGAGCTGAGGCCAAAGGTCACAGAATACATAG

GTAATGCAGGAAGACTGACAGTGCGGACCGAGAGGAGTCTCAAAATCCCAAACAGAGAAACCCAGGGGCC

ACAGCCAGGCCCCGGCCAGCCCCATGGAAACCCGCCTCGCAGGGCCACTGACCGTCCAAGTAACAGACTT

GGCTGTCCCGGAATGCAGGAACCCCAAACGGAACCCATAGGTGCCAGGGTAGGTCTTCGGGGAAGGGACA

GAGGATGATAGGGGCCAGGAGGGGGCCGGTCCAGGGGCTGTGGGGGCAGAAGTGGCTGGCATCCTGGGAG

CATCATCTGAGTCTTCGTCTAGCCAGTTCACGACGCTCTCTGGGAGCAGCAGCTCATCCACTGCTGGGCA

CAGCTCCGAAGACTGTAGGGGGCGAGACCAGAGTCAAGGGTCAGGTCCCTAGGTCCCCAGCTCCCAACCC

CCGCCCTGGACCTCCCCACACCCAGTCCTTACCAGAACATTGTTTTCAGGAAGCCTGGAAGATGAGAGCA

GAAAGTCAGTGCTGGTTTTGCTTCCTCTGGGCTTCTACTTGGGGGGGCGGGGGGGGGGGCTGGGGCTGGG

GGTTGGGATTGGGGTGGGTGGGCAGGGGTCCCAGAGGGCTGGTGGGGAGCCTGGCCTGCCCTCCACTTAC

AGGTTCCACAATTCTGAAAATGTCTCCTGGCTCAGAGGGGGGTCGATATTGAGCTCTGACTGTGGCTCTT

GCATCGCAGCTCCTGGGAAGGGAGTGTGGGTGCTGTGGAGGGCAAGGCGAGGGGTCAGCGGGAGACAAGC

CCGGGCCCGGGTCCCTCAGCCCCGAGGTGGGAGGGACCCTTCCAGCAACATTATACCAGGGCCTGGGATG

CCTTGGCTCCCCCACCTGGGCCTCCCCAGGAGCAGGGCTGCTCCTGCCCATTCCACCCCTGGGCGGCCTA

CCCCCACACATCCTCAAGGTACAAAGATAACCAGAAAGGGGATTCTAGCCCTCTCATTACAGAGTCCACA

AGCCTTCCTGGTTATAAGGATGAAGAACGTCAGCCCACACCCTCCACCTGGACGAAGATACCTGCATGGA

CCCCCTCATTCCCCACCTGATCCCCTTCATCACGAAGCTTCCTAAGGATCTGCTATCTCCATTCCACCCT

TGGCTTGCAGAGTCTTCAGGACCTCCCGGCCGGCATCCCCGACCTTGTCTTCCAGTATTCTCCAGCCAGT

GGCTGCCCTTCTCTGCTGCCACACACACACCCCAGTTCATTGGCTCGGGGTTCCCTCTATTGGGAACCCT

GGTCCAGCCTCTGCTTGCAGGGGAGTCACCTTCTAGCAGATCATCCCGAATGTCACAGCTCCTCAGGGAT

GTCTGCCCAGACCACCCAGGCAAAGACTGCCAGCCAAACGCTGTCACCAGTTTTTTTAAAAAAGATTTGA

TTAATTAATTTATTTGAGAGAGAGAACAAGAGTGAGAGAGTGGCAGAGGGAGGGGGAGAGGGAGAAGCAG

GTGAGCTACCTGTCCGGCTCACTGCAGCACTAGCAAAATGCCTCTCACCTGGTTGGGCTCACTGTGGTCA

GCAAGTGAATGGTGCCGAAGCCAGGAGCACCTTGTGAGGTGTCCCCAGGAGCCAGCAGGTCAGAGGGAAG

GTCCCAGTCTGGGCCCTGGCGCTGTACTAGGCTGCGCTGCCACCATCTCATGACATCTTCCCCAACTCCC

GAGTTTGTCAAGAGTTTTGCAAAAAAACACACCCACTGCAAACCAAAGTCCCTATTCATTACCTTTTTGT

CTTGTAAAGCACTGACAAGCCTACCGGCCCCACCCTCACCCCCAGCAGCTCTTCCCATTAGCATCTCCAT

GAACATTCACCTCTACCTGGATTGAAGGCCTGCTTTCTTATGTCCTCAGAGTTTGTGAAATCCTTTCTTG

TGGGTTATCTCATTTGTCCCAGAACCACCCTGGCTGCCCAACAGGTGAGGAAGCTGGTCTCAGAGAGGTG

GAGGGACTCGCCCACCGCACACAGGCAAATGGATGAGCTTTTATTCGAAATGACCACTCGTGGGGTGCTT

GGGTTGCTCAGTGGTGGAGCACCTGCCTTTGGCTCAGGTTGTGAGCCAAAGGGTCCTGGGATCGAGTCCC

GCACTGGGGTCCCTGTAGGGAGACTGCTTTTCCCTCTGCCCGTGACTCTGCCGCTCTGTGTCTCTCATGA

ATAAATAAAATTTTAAAAAAAGCTTTTGAGATTTTATGGAAGGGAGCTCAGAAGTCAAAGCCCTAGGTCT

GACATCGTATCATTACTTATATTTGCTGTGCAACTCTACCCATCCCAAGGGCTAGCCTTTTGGCTTCCTT

ACATGCAGGAATTGAGTTTTGCTCATCTTTGTTTAGTGAAAAATACCTAGGATGGGGAGACATGAGCAGC

TGTAGCAGGGCATGGGCTCTGAGGCAGACAGGCTAGGCCTAAATCCCATGCCCACCACCTAATAGGTGGG

GAGGTTACTTAACCTCTGTGCCTGGCACAGTAAGAGCTCAACAGATGTGTTTGTTTATAGGATTTTCCAT

TCTATTAGTTTGATTATTATTTTAGACCCAGAACTTTGTAATTGAGTGGGTGGGGCTTCTGTAGTCCTGC

CTCATTAATGCTAGTGCCCCTCTTTCAGGGATCTCTGTTCAGTAAATATGCCAGGAGGGGCTGTCCTGGG

ATGCTAGGCCCGAGGTCCCAATCCCACTGGGAGGGAGACAGCCTCATAGACAAAGGAGGTCTCTCCCTCT

CTTGGTCCTTGGCAGCAAGTAAATTCTGTTTTTCTCAAAAAAGCAGGCACTATCACCAGGAAACCTTTTT

GGAGCATGTACTTCCCATGGATGAGGATCAAGGGCCATTAAACTAAATTGTCAGAAGCTCAACCAGACCT

TCCAAATGGCTGGGGAGGCAGGACTCATAGATATCCCTGGTGGGAAGGCCAAACTCAGCTGGGACCTCAT

CCAGTTGCCCACCCCACCACCATCCTAACAATGGCTGTTGTTTGTAATCCGCACAGGGGCTCACTGCTCC

GGTGGCTTATCTCATTAATCCTCTCAAGAAACCATAGCAGTGGGTATCTTCAAGCTGAAGGAACTGAGGT

TGAAGGAATTAACTCACTCATTCAGATTCAGAAAACTCCCGAGCCAGGGATTTGAATCTGGGCTGTTCAA

AAGCCACTGTCCAAGCTCTAAATAATTATGCTCCTCTGGCTTCATCCAGAGTCTGTAAAAATAATAATAA

TAATAATATTTGTTTGGTGCAAAAAGGTTTTTTTGGCTGAAAGCTATTTGGGAAAACACGATGATTCTTA

AAAGCAGCAAGGCAGTATCTGGTAAGACTCAACAACTTACTACTTTGCAGAATCACTTCTAGGTAAGACT

CCCTTAGAAGAGTTTTGTATGGAGGGAGCAGGAGTTCTGCGCAAATACGTTCATATCAGTGATGTTCTCC

GTGACAGGAAACATCACTATCCATCCGTAGGGATTGGCTACATGAAATACAAGAGTTTACTGCAGCAGTT

TAAGGAAATGAATGTATCTGTGTGTACGTAACTAGATAGATATAGCTCAGATTGATTAAAAAAATACAAT

TCGAGGGGGAAAAAAAAGAAAAACATGGTAACCTAAGTAGTTTATGTGAATTAAAAATATAAAATGAGTA

CAGTTGACCCTTGAATAATGTGGGGGTTGGGGGCATCGAACCCTGTGCTATAGAAAATTCATGAGTTGGG

GCACCTGAGTGGCTCAGTGGCTGAGCTTCTGTCTTGGGCTCAGGTCGTGATCCTGGGGTCCTGGAATCGA

GTCCCGCATCAGGATCCCCACAGGGAGACTGCTTCTCCCTCTGCCTATGTCTCTGCCTCTCATGAATAAA

TACATTTAAAAAGTCTTAAAAAAAAGGGATCCCTGGGTGGCGCAGCGGTTTGGTGCCTGCCTTTGGCCCA

GGGCGCGATCCTGGAGACCCGGGATCAAATCCCACGTCGGGCTCCCTGCATAGAGCCTGCTTCTCCCTCT

GCCTATGTCTCTCTCTCTCTCTCTCTCTCTCTCTCTCTCTCTCTGTGACTACCATAAATAAATAAAAATT

AAGAAAAAAAAAAGTCTTAAAAAAAAAAAATCCAGGGACGCCTGGGTGGCTCAGTGGTTGAGTGTCTGCC

TTTGGCCCAGGGCATGATCCTGGGGTGCCCAGATGGAGTCCCGCATGGAGCCTGCTTCTCTTTACCTGTG

TCTCTGCCTCTCTCTCTGTGTCTCTCATGAATAAATAAATAAAATCTTAAAAAAAAAAAAAATCCAGGTG

TTATCTCTTGACTGACCCAAAATTTAACAGCTTACTATTGAGAAGCCTTACCGATAACATAAGTAACTAA

AACATATTATATATATATATTATATACTACATTCTTTTTTTAAAAAAAGATTTTATTTATTTGAGAGGGA

GAGAAGCAGACTACCCGCTGAGCAGAGAGCTTGATGCAGGGCTGGATCCCAGGACCCTAAGATAGTGACC

TGAGCTAAAGGCAGACTGGCCACCTAGGTGCCCCCCATATACTATATTCTTAAAGTAAGCTAGAGATAAT

GCTATTAATATAATCACAAGGAAAAAAAGAAACATTCACAATACTGTTACTGTATTTATCCAAAAAACCC

ATGTATAAGTGGTCCTACACAGTTCAATCCTGTGTTTTCTAAGGGTCAACTGAATTTATTTCTTACCAAC

ATATATATGTGAAAGTATGAAAGACAGATAAAAAGAATTAAAACTTACACTCATGAGACTGGGGGTACCA

TCAAAGCGACCTTATTTGATTAATGAAGAAAAATTTAAAAAGATGGTATTCATACATTATTTGTTTATTA

TAATATGAACATGACAAAATGTCAAGTGTTATAATTTCCTCTAGCTTCAAGAATAATCAACATTCTGCCT

GTATTGTCTCCCCTATACTTCTAGTGGCTCCGCAGAAGCCCACAGATATTTTTGAGTTACAATCTATGTA

AGTTGAAATGCACAATCTTAATTTTGCCATTTAGATAACAAAGTTTGACACATTTTGATATAACCTTGTC

ACCCAGCCCTACCAAGCATAAGACACTGCTGCCATGTTTCCTTCTGAAAGTTAATATATTTTATGCTTAG

ATCTACTATCCATCTCAAATTGTGTATAGTATGAGGTAGGGGTCAGAATCCACTCAGCTCTGCTTGTTTC

AGCACTCTCTTCAGACTTTCCTTCCCCCATGGATTCATTTTGGAGCTTTTGTAGAAAACCATTTGATTAC

ACTTATGTGTCTTTTCCTGGGCTCTATTCAGTTCATGGTTGTTGTCTTATGGTAGCTTTACAGAATTCTT

GAAGTCAGCTAGTTTAAGACCTCCAGCTTTGTACTACACTTTCATTTTGGAATTGCTTGTCAGTTTCTAC

AAACAAAACCAGCTAGGATTTTGATGGGGATCAAATGAAATCATCTTGGAGTACTGACATCTCAACACTA

TTGAGTGTTCCATCTATGAATATGGTACTGTTACTTAAATCTTCCGGGCAGCCCGGGTGGCTCAGCGGTT

TGGCACTGCCTTCGGCCTGGGGTGTGGTTCTGGGGACCAGGGATCGAGTCCCACGCAGGCTCCCTGCGTG

GGGCCTGCTTCTTCCTCTGCCTGTGTCTCTGCCTCTCACTTTGTGTCTCTCATTAATAAACAAATAAAAT

AAAATAAAATAAAAATCTTCCTTAAATTCGCTCAGCAATATTTTGCAGTTTCTAACATAGAACTCTTGTC

CGTCTTTCTTCAATTTATCCCTGGGTATGCCATGTTTTTGGATGCTTTGTAAATGCCACTGCTTTTATAA

TTCATTTTCCGACTTCAGATTCAATGGTTGTTATTACATAAAAATACAATTTATGTATATTGCCTTTGTA

ACCTGTGACCTTGCTATAATCCTTTATCAGTCATGATAGTTCTTCTTTTTAAGAGGTTTCATTTTTTAAG

TAATCTCTGCATCCAAGGTGGGCCTCAAATTTACAACTCTGAGATCAAGAGTCATGTATTCTACTGACTG

AGCCAACCAGGTGCCCCTTGTGAGTTTTTCAATGCTTCTTCTATTAGCCTGGTTAACACCTTCAGTATAA

TGTTGAAGGGGTGAGAATCAAATCCTTTCCTAGTTCCTCATCTTAGAAAACAAGCATTCAGTCTGTCACT

GTTACGTATTGATGGTAACTTGATTGGGGGTGCTCTTTATCAGACTAAGTTTTCCCTTTCCTAGTTTGCT

GGGAGGTTGGCTTTTCTTTTCTTTCTTTTTTTAAAATCATGAATAGGTGTTGCGTGATGTCAACTGCTTT

TTATGTATCTGTTGAAATGATCATAGGATTTTTATCCTTCAATCTGTTTATGTGGCAAATTACACTGATT

TTTTGAATGTTAAAACATTGCCGTCTAGAGATAAACTCCACTTGATTAGGATGTATTTATCCGTTTTATA

GATAATCAGATTTACTTTGCTAAGATTTGTGAGATTTTTGCATATATACATATGTGTGTATATATACACA

CATATGTATATATGCTGGCAGGAATATTGGTCTAAATGTTTTCTTGTAACATTCTCGTCTGGTTTTGATA

TGAGGATTCTCAGGATATCATGAAATTAGTTGGGAAGATGTTACCTCTTCTACATTCTGAAAGTTTTTGT

AAGATTGTTATTGTTTCTTCCTCAAGTGTCTGAAGAGAAGAAGAAATAGAATAGAAAATAGCATTAACAC

AGTAGACTTAAACCCAAACCTTACAATAATTGCATTAAATTAAATAGACCAAGCACTCCAATTAAAAGTG

ATCCAGTGAGGGGGGAAGGAGGGCTGTTTCAACAAGTGGCACTAGAAGAACTGGATATACATCTAGGAAA

ACCCCAAAAGAAAACAGAACCCCTACCTCACATCACGATAAAAATTACTCTTAATTAATTAGTTAATTAA

TTATGGCTCAAAGACCTAAATATAAAACCTAAAACCCTAAAGATTCTGGAAGAAAATATCCTAGAATATC

TTCATGATCTTGGGGTCAGCAAGGATTTTCTAAACACACAGAACACATTTGAAAGACAGAAGATAAATCT

GCTGCTGCTCTGCCTTAGAGGCTGACAGAGAGGCAAGAAACAGGTCAGGAGAAAATAGATACTGACAAAA

GACTCCTATCCGGAATATATAAAGAACTGCATGCAGCTGAACGCAAGTTAAAATGGCCAATAAGCACGTG

GAAACTGATCACAGCTAAGAGAATTGCAACTGAAAACCACAATGATGCACCACATTAGAAAGTCTGACAA

AACCAGATGTGAGGAAAGACAGCTGTTGCTGGTGGGTACATAAAACAGAACAACCACTTTGAAATATACT

AATTTTAAAAATCAAATTTAGATCTCTCTCACCTAGCAAATTCCACTCCCAGACGGTTATAAAAAACAAA

AACAAAACAAAACAAAACACACACACACACAAACACCCAAAACCAACCCCATGAAAACATAGGACCACAA

AGAGAATTGTACAAGAATGTTAATAGCAGCTTTATTCAAAATAACCCCGAACGGTTAACAACCCAAGCGG

TCACCGAAAGGAACATGGATTAAAAAAAACCTCACGGTTTATTCATCCAGTGAGATGTGCTAAATACAAA

AATACAAAGGTGCAAAAAACACTGATACACTACTGATACAGTAGTACTGGTTACACAGGAACAAGGATGG

GTGTCACAGGTATTATGCTGCCGGCGAGAAGCCTACACAAAGGAGCACACACTATGTGGGTCTATTTACA

CGACGCTCTCTTCAACAGGCACAAGTAGTGACAGAACTCCAAGATGAGGGTCAGAGACCCCCAGAGGGGA

GAGGCCCAAGAACCTTCTCTGGGGCGATGACAATGTGTAAATTGTGTCATTTGACAGGCTGCTGGTTAGC

AGCCCAAACCTGTCTAATTGCAGTGAGTGTAAATGCTACCTCAATTTCAGAACAAGCCGCACACAAGCAC

CGCGTGCACTGGCCGGTACCGAGCAGGTGCACAGGTAGGTGAGCGAGCGCAGGCTGGAAGCACCGCGCGC

CCGGAGTGACTGACAGGCGGGAGGCGGTATCCCGTCCCCTCGGCTCGGCTCGGCTCGGCTCGGCTCGGCG

GGCCCCGCCCCCTCAAGCCCCGCCCCTTCCGCCCTCACGACGTTCGCTCTGCGTCGGAACTCGGAATGGC

TGAGTCCCGAGCCGGATGCCAGAGAAACCGTGTCTCGGCCCCACACAAACGGCGCATCGTCGCATTCTGC

CATCGTTGTCATTCTCAAATGTACTCGTGCTCGAAAATACCCGGGACTTGCCGTCGGCACGCTTCTGGAG

ACCTCGTCCCTCGCCCGCCCAGATTGCAGGCGATGGGGTGGGGCGCACCATTCAAGGAGTGGGGGAAGGC

TCGAGGTTTGCATCCAAACAGACCCTCCCTCCCGGCCTGCCAAAAGTAGCAGCTAAGGAGTTCAGGGGAA

AAAAAAAAAAAAAAAAAAAAAAAAAGAAAGAAAAGAAAAAATAAGAAAGAAAAAAGAAAAGAAAAGAAAA

GAAAAGAAAGCCAGTAGAGGAGGCGGCGCGCAGCCTCCCTGAGCACGTCAGTCTAAATGACACTTTCCCT

CTCCTCGGGGGAACAGTGAACTGCTCCGGAAAAAAAACCGGAAACGCTGCGGCAAGGGGCAGAAATGTAA

ATGTTCGACCAAACAATAACAGAGCTCGCGGGCCTCCTGGACCCGGACCGTCCCGCCGGGCGTCGCGGGC

AGACTGCTCGTGGAGCGCTACCCACGTCCACGCCAGGCAGTCTACTAGGATCGTCTCCATTTAGTGGAGC

CAGGGGCCGCTGGGGAGATCCCTCCGGGAAGGACAAAGGTCCGGAAGTTCTGGGACCTGAGCGGCTTGGG

CTTCCTGGATCGACCCCCCCGAATGTGATCATGTCGGGACGGAGCCCGAGAGGACCCTCCAGGGATGCTG

CGGGCTCGAAGATCTCGGGGGTCCCCAAAATCCCGAGATCGGGGAAAAAACAATCTCGGATCCTGATGCT

TTGTGCGTCTTATTTCGGGGTTCTTCACACCCGGCACGGGGAGAGAAAGGGAGGGGAAGGGTGAGGAGGA

ACCCCTTCGGCCGGCTGCACGTCCCCCTCCTATAAAGCTGAGTCCGGCTGCTCCCAACAGCCCCTTCATC

TGCACCCAGGCCTCCCAGCGGTGCATTCCGATATTGGTGCCGCCAGCCAAACTTCCATTCCCCCGCGATC

GCTGATCGCGTGCCCCACCCCCAGACCCCAATTCTCCCCAAGCTCAAAACACACAGAGCCGAGAGCCGGT

GACTCAGACAGGACTCATCTGGCTCAGTCAGGAGCTTACCCGAACCTCAGGGGAGCGTGTCACCGTCCTG

GGAAGCACGCTCCGAGGCCGGACGCCAGAGGATCCCGGAGCCCGGCGGCTACCTGCGCCCAGGATGGTGG

CTCTGGACTTTTGAGAAGCTCAAAACT

>Ensembl_114_ROS_Cfam_1.0_Forward_:5:32665394:32678755:1_5 dna:primary_assembly:

TGGTAGTAAAGTTTTATTGTAAAATAAGAGATTGATATAAAAACGGGATATAAAAAGGGA

AGAGGAGGAGGGGTGGGGGTGATCAATGCATGAATGCAGATGTGCCTCACAGAGTGCAAA

AGAAATTGATCCTGAGCAATTGGGGGCGGGGGGGGGGGGGAAGCAGTAAAAGCCCTTGGT

AGACAAAGATCCTACACCAGGGATGGGATTCTGCAGGGTGTGAGATGGGAGTGAGATTTC

ATCTTAGGTGCAGGGGTCTCCCAAAAAGGCTGGACAGGATTTGGCCAGGGCAGGGAATGA

GCACCCACACAGGCACTAAGTCACAGACATGGGATAAAAGGTGGTTTTGGGGTCAGACAG

ACATTATTTCATTCATCCTCACAAGCTCAGTGAGGTAGGTATGGTTAATTAGCAATTCAT

AAAAATGGCCTGTGAAATTAGGAAAAATTCAGCCACTGGCTACAATGATAGAGAACTGGG

CTCATGACTCAGTGTCTTAGGGGCTATAAAATGCTCCCAACATTCAGGAACATTTTCCTA

CCTCTCCCATGGTTCTACACAGTAAAAAACTTAAAAACCAGGCTGGAACGTCCTTTATCT

CCCGACCTACCAACTGTGCAGGCCAACTTCTTTAGTAGAGACTTACGGGAAAGGCAAATG

GAAGAATCTAGGGTGTTTCTGAGGCACATCTGTACCATGCAAAGTAACACACCGGAATAG

GTGTGCTCAAGCTCCCAATTCCAAAAATGCCAGGGAAGGAGGACGAGAGTGAGGGTGTGC

GTGTTGGGGGAGGACAGGAAGCAGGGGAAGTCAGTCTGAGTCGGGCCCTTCTCTCTTGAA

CATCAGTTTTTTATGGCGAGAGGTAGATTGCCCCTTCTTTGCCTTCAGGTGGCTGTTGGG

GACACAAGGTGGAGGGGAAGAGACGATATTAGCAAGTGGGAGCCTCGGGGCTCCTTGTCC

TAGATCTAGGCTTGGAAGGGTCCAAGGCTGGAGTAAGGGAATTTTTTTTTTTTTTTTTTT

TTTGGAATGAGGTAATTTAAGTTAAAATGCTGTAGACACATTTACTGAGCACCCAGGGAA

CTTCTGTGTGTTCGCTTCCTCTCCCCTCACCCCATCTGATGAACAAGGAAACCGAGCCCC

AGAAGGGGAGATGCAAGGCCACACGGATCTGCTCATCCTTGGCAGGGCTGGGGGTCTGGC

CCAATCCTGCTGTGTCCAGTATTTTCCCACACGGTTATCAGGTCCCTATTGGAGACCCCA

CTTGGCACTAGGGGTTGCTGGAGATGACAAACACCCATGCTTGTCCCTTAAGGGGTTTGG

GGACCTCCCGAGGCAGAAAAGGGAGTACAGGATCCAACTGGGGGTATGGTAGAAGCAGGT

TTGCCCAGGGTGGGACCAACCATGAGGGGATCAGTTCCCTTCCACCTTGAAGGTAGGAGT

AGAATGGAATCCTGTGGCTTTCCAACATATGAAGGCAAGGGGGTGGGGCTGCAAGCCGGC

CCAGGTCACTCACCTGGAGTGAGCCCTGCTTCCCCCTGGCTCCTTTCCACTCTGGGCATC

CTTCAGCTCCAAGGCTTCATTCAGATTCCTGAACATCTCATAGCGTTCACGCCCACGGAT

CTGTGGCAGTGGGGGGGAGGAGGAGAAAGAATAAGAGGGGGAAGCCACAGTACCATTTAA

CTCAGACTAAGGTGTAAGTGCCAATCTGCAGCAAGGCACAGCCCGCAGCCACTGTCAAGG

CACCGCTGCCCCTTGATGGCAAGTGTGCTAATTGCAGGCGGAGCAGTCAAAAGAAAACTG

TGCTCTTGGCCCTCAGCTTGAAACAAATCTTCAATCCAAGAATATCAACTTCAAGAATCA

GGGACAGGACCAGAGAAAAGGATGGAGAGAGGAACAGGCCTAGCCTCACATGTGCTCCAT

TCACATTTTTTTCTTCCCTCATCTGATCCCGCTAAGCGGGGGCAAGAGCCTGCCTTATCT

GTTCCTCCCAGCCTTGGTACCTGAAGGGTGAAATATTCTCCATCTAGTGGCTTCTTCTTT

TGCGGGGGAGAGGAGCTGGTGCTGGGAGGCAGTGCTGGGAAAGAGAGGAGGAAAAGACAA

GAGAAGAGTATGTTTTGAGCAGAATTGCACCCTCTGTATCTGCCCCACATCCTCCTCTTT

TTTTTTTTTTTTTCAAGATTATATTTATTTATTCATGAGACATACACACAAAGGCAAAAA

AATAGGCAGAGGGAGAGGCAGGCTCCCTACAGGGACCCTGACACGGGACTCGATCCCAGG

ACCCTGGGATCATGCCTGATGCTCAACCCCTGAGCCACCCAGGTGTCCCTCCTTCACCTC

CTCTTGTCTTGCTTGCTTACCTCGCTTGGTACTCCCGGGGGGTGGCTCAGGACAAGGCTC

CCCCTTCTTGTGGAAATTCTCCTCCTCAGTCCGGCGGTCTCTCCCGGGACAGGCACAAAC

GCGTACCTCAAAGCTGTTGCGTCCCAGCACGTTTCCACTACCCAGGTGAGAAGAGAGAAG

CAGGGGGCCGTGAGGGTGGTGGGCCCCACTGACTCCTAGCCACCCTCATCCTACGTAGAG

CCAAAGCCTACAGACTATTGAGAAGCCACAGGTCAGGGAGGTCCCAAAGCCAGAGGAAAG

AAAACCAGGGGAGGCGGATGGCAGAGGAGATTCCACATTGGGACAGGAATGGATGGGAAG

GATGGAGGGGGGTACAGTAAGGAAGTGGCCAGGAGCAGGGTGGCAGGCAGGTCCCTACCT

GGAGTCTTCCAGGGTGATGATAGTGAGGATGGGCCGCCGGTTCATGCCTCCCATGCAGGA

ACTGTTACACATGTAGTTGTAGTGGATGGTGGTATAGTCAGAGCCAACCTGAGGTAGAAG

GTAGGCCCAGGGTCGGGCCACTCAGCCCTGGGCAGGCCTGTAGTCTATCAGCCTCAGTCT

TTCCCAACCGCAGAATGGGATGACCCACCTGCCTCTGCACTCCTCACAGGGCTGCGGGGA

GGTTGAAGGAGGAGAAAGCCCCCTCAACTCCCGCCTCACAAATCCCTCCAGCCCCCTCCT

CCCAGAGACCCCTCAGATGCCAAACCAGACCTCGGGTGGCTCATAAGGCACCACCACACT

GTGTCGAAAAGTGTTTCTGTCGTCCAGGTACTTGGCCCGCAAATTTCCTTCCACTCGGAT

GAGATGCTGAGGAGGGGCAAGACCTAAGAGCCATCGGGGAGGAATCAGGGCTTTGGGCCC

TCTGGGGAGCAGCCTTGTCCCATCTGTAGCCCCCGATGGCTTACCGTCACTACTGTCAGA

GCAGCGTTCATGGTGGGGGCAGCGCCGCACAACCTCGGTCACGAACTCCGACTTCTTATA

GATGGCCATAGCGCGGACGCAGGTATTGGGTGGGGGTGGGGAGCTGACCCACAGCTGCAC

GGGGCAGGTCTTCGCCAGCTGGCAAAACAACTTGTTGAGGAGAGGGGAGTACTGTAAGAA

AAGGACAGATGGACAGGTCAGGGCATGTGTGCCCGGGGTGGGTGGTGAGAAGTAGAGGGG

TCGGGGTCTATTCCAGAAGCTTACTTTATTTTAGACACACAGAGAGAGGCAGAGACACAG

GCAGAGGGAGAAGTGGTATGGGATTCGATCCCAGGACCTGGGGCTCACACCCTGAACCAA

AGGCAGACGCTCAACTGCTGAGCTGCCCAGGCATCTCCCAGAAGCTTACTTTAAAAGCCA

AGGAATGAACAAGTGAAGGAAAATGAAGAAGAGTCCCTTTGCCCCATGACAGGAAAGGTG

AAAGCCCAAGGTCAAGGGGGGAAATGTCAACTTTGACACAGAGCTGAGGCCAAAGGTCAC

AGAATACATAGGTAATGCAGGAAGACTGACAGTGCGGACCGAGAGGAGTCTCAAAATCCC

AAACAGAGAAACCCAGGGGCCACAGCCAGGCCCCGGCCAGCCCCATGGAAACCCGCCTCG

CAGGGCCACTGACCGTCCAAGTAACAGACTTGGCTGTCCCGGAATGCAGGAACCCCAAAC

GGAACCCATAGGTGCCAGGGTAGGTCTTCGGGGAAGGGACAGAGGATGATAGGGGCCAGG

AGGGGGCCGGTCCAGGGGCTGTGGGGGCAGAAGTGGCTGGCATCCTGGGAGCATCATCTG

AGTCTTCGTCTAGCCAGTTCACGACGCTCTCTGGGAGCAGCAGCTCATCCACTGCTGGGC

ACAGCTCCGAAGACTGTAGGGGGCGAGACCAGAGTCAAGGGTCAGGTCCCTAGGTCCCCA

GCTCCCAACCCCCGCCCTGGACCTCCCCACACCCAGTCCTTACCAGAACATTGTTTTCAG

GAAGCCTGGAAGATGAGAGCAGAAAGTCAGTGCTGGTTTTGCTTCCTCTGGGCTTCTACT

TGGGGGGGCGGGGGGGGGGGCTGGGGCTGGGGGTTGGGATTGGGGTGGGTGGGCAGGGGT

CCCAGAGGGCTGGTGGGGAGCCTGGCCTGCCCTCCACTTACAGGTTCCACAATTCTGAAA

ATGTCTCCTGGCTCAGAGGGGGGTCGATATTGAGCTCTGACTGTGGCTCTTGCATCGCAG

CTCCTGGGAAGGGAGTGTGGGTGCTGTGGAGGGCAAGGCGAGGGGTCAGCGGGAGACAAG

CCCGGGCCCGGGTCCCTCAGCCCCGAGGTGGGAGGGACCCTTCCAGCAACATTATACCAG

GGCCTGGGATGCCTTGGCTCCCCCACCTGGGCCTCCCCAGGAGCAGGGCTGCTCCTGCCC

ATTCCACCCCTGGGCGGCCTACCCCCACACATCCTCAAGGTACAAAGATAACCAGAAAGG

GGATTCTAGCCCTCTCATTACAGAGTCCACAAGCCTTCCTGGTTATAAGGATGAAGAACG

TCAGCCCACACCCTCCACCTGGACGAAGATACCTGCATGGACCCCCTCATTCCCCACCTG

ATCCCCTTCATCACGAAGCTTCCTAAGGATCTGCTATCTCCATTCCACCCTTGGCTTGCA

GAGTCTTCAGGACCTCCCGGCCGGCATCCCCGACCTTGTCTTCCAGTATTCTCCAGCCAG

TGGCTGCCCTTCTCTGCTGCCACACACACACCCCAGTTCATTGGCTCGGGGTTCCCTCTA

TTGGGAACCCTGGTCCAGCCTCTGCTTGCAGGGGAGTCACCTTCTAGCAGATCATCCCGA

ATGTCACAGCTCCTCAGGGATGTCTGCCCAGACCACCCAGGCAAAGACTGCCAGCCAAAC

GCTGTCACCAGTTTTTTTAAAAAAGATTTGATTAATTAATTTATTTGAGAGAGAGAACAA

GAGTGAGAGAGTGGCAGAGGGAGGGGGAGAGGGAGAAGCAGGTGAGCTACCTGTCCGGCT

CACTGCAGCACTAGCAAAATGCCTCTCACCTGGTTGGGCTCACTGTGGTCAGCAAGTGAA

TGGTGCCGAAGCCAGGAGCACCTTGTGAGGTGTCCCCAGGAGCCAGCAGGTCAGAGGGAA

GGTCCCAGTCTGGGCCCTGGCGCTGTACTAGGCTGCGCTGCCACCATCTCATGACATCTT

CCCCAACTCCCGAGTTTGTCAAGAGTTTTGCAAAAAAACACACCCACTGCAAACCAAAGT

CCCTATTCATTACCTTTTTGTCTTGTAAAGCACTGACAAGCCTACCGGCCCCACCCTCAC

CCCCAGCAGCTCTTCCCATTAGCATCTCCATGAACATTCACCTCTACCTGGATTGAAGGC

CTGCTTTCTTATGTCCTCAGAGTTTGTGAAATCCTTTCTTGTGGGTTATCTCATTTGTCC

CAGAACCACCCTGGCTGCCCAACAGGTGAGGAAGCTGGTCTCAGAGAGGTGGAGGGACTC

GCCCACCGCACACAGGCAAATGGATGAGCTTTTATTCGAAATGACCACTCGTGGGGTGCT

TGGGTTGCTCAGTGGTGGAGCACCTGCCTTTGGCTCAGGTTGTGAGCCAAAGGGTCCTGG

GATCGAGTCCCGCACTGGGGTCCCTGTAGGGAGACTGCTTTTCCCTCTGCCCGTGACTCT

GCCGCTCTGTGTCTCTCATGAATAAATAAAATTTTAAAAAAAGCTTTTGAGATTTTATGG

AAGGGAGCTCAGAAGTCAAAGCCCTAGGTCTGACATCGTATCATTACTTATATTTGCTGT

GCAACTCTACCCATCCCAAGGGCTAGCCTTTTGGCTTCCTTACATGCAGGAATTGAGTTT

TGCTCATCTTTGTTTAGTGAAAAATACCTAGGATGGGGAGACATGAGCAGCTGTAGCAGG

GCATGGGCTCTGAGGCAGACAGGCTAGGCCTAAATCCCATGCCCACCACCTAATAGGTGG

GGAGGTTACTTAACCTCTGTGCCTGGCACAGTAAGAGCTCAACAGATGTGTTTGTTTATA

GGATTTTCCATTCTATTAGTTTGATTATTATTTTAGACCCAGAACTTTGTAATTGAGTGG

GTGGGGCTTCTGTAGTCCTGCCTCATTAATGCTAGTGCCCCTCTTTCAGGGATCTCTGTT

CAGTAAATATGCCAGGAGGGGCTGTCCTGGGATGCTAGGCCCGAGGTCCCAATCCCACTG

GGAGGGAGACAGCCTCATAGACAAAGGAGGTCTCTCCCTCTCTTGGTCCTTGGCAGCAAG

TAAATTCTGTTTTTCTCAAAAAAGCAGGCACTATCACCAGGAAACCTTTTTGGAGCATGT

ACTTCCCATGGATGAGGATCAAGGGCCATTAAACTAAATTGTCAGAAGCTCAACCAGACC

TTCCAAATGGCTGGGGAGGCAGGACTCATAGATATCCCTGGTGGGAAGGCCAAACTCAGC

TGGGACCTCATCCAGTTGCCCACCCCACCACCATCCTAACAATGGCTGTTGTTTGTAATC

CGCACAGGGGCTCACTGCTCCGGTGGCTTATCTCATTAATCCTCTCAAGAAACCATAGCA

GTGGGTATCTTCAAGCTGAAGGAACTGAGGTTGAAGGAATTAACTCACTCATTCAGATTC

AGAAAACTCCCGAGCCAGGGATTTGAATCTGGGCTGTTCAAAAGCCACTGTCCAAGCTCT

AAATAATTATGCTCCTCTGGCTTCATCCAGAGTCTGTAAAAATAATAATAATAATAATAT

TTGTTTGGTGCAAAAAGGTTTTTTTGGCTGAAAGCTATTTGGGAAAACACGATGATTCTT

AAAAGCAGCAAGGCAGTATCTGGTAAGACTCAACAACTTACTACTTTGCAGAATCACTTC

TAGGTAAGACTCCCTTAGAAGAGTTTTGTATGGAGGGAGCAGGAGTTCTGCGCAAATACG

TTCATATCAGTGATGTTCTCCGTGACAGGAAACATCACTATCCATCCGTAGGGATTGGCT

ACATGAAATACAAGAGTTTACTGCAGCAGTTTAAGGAAATGAATGTATCTGTGTGTACGT

AACTAGATAGATATAGCTCAGATTGATTAAAAAAATACAATTCGAGGGGGAAAAAAAAGA

AAAACATGGTAACCTAAGTAGTTTATGTGAATTAAAAATATAAAATGAGTACAGTTGACC

CTTGAATAATGTGGGGGTTGGGGGCATCGAACCCTGTGCTATAGAAAATTCATGAGTTGG

GGCACCTGAGTGGCTCAGTGGCTGAGCTTCTGTCTTGGGCTCAGGTCGTGATCCTGGGGT

CCTGGAATCGAGTCCCGCATCAGGATCCCCACAGGGAGACTGCTTCTCCCTCTGCCTATG

TCTCTGCCTCTCATGAATAAATACATTTAAAAAGTCTTAAAAAAAAGGGATCCCTGGGTG

GCGCAGCGGTTTGGTGCCTGCCTTTGGCCCAGGGCGCGATCCTGGAGACCCGGGATCAAA

TCCCACGTCGGGCTCCCTGCATAGAGCCTGCTTCTCCCTCTGCCTATGTCTCTCTCTCTC

TCTCTCTCTCTCTCTCTCTCTCTCTGTGACTACCATAAATAAATAAAAATTAAGAAAAAA

AAAAGTCTTAAAAAAAAAAAATCCAGGGACGCCTGGGTGGCTCAGTGGTTGAGTGTCTGC

CTTTGGCCCAGGGCATGATCCTGGGGTGCCCAGATGGAGTCCCGCATGGAGCCTGCTTCT

CTTTACCTGTGTCTCTGCCTCTCTCTCTGTGTCTCTCATGAATAAATAAATAAAATCTTA

AAAAAAAAAAAAATCCAGGTGTTATCTCTTGACTGACCCAAAATTTAACAGCTTACTATT

GAGAAGCCTTACCGATAACATAAGTAACTAAAACATATTATATATATATATTATATACTA

CATTCTTTTTTTAAAAAAAGATTTTATTTATTTGAGAGGGAGAGAAGCAGACTACCCGCT

GAGCAGAGAGCTTGATGCAGGGCTGGATCCCAGGACCCTAAGATAGTGACCTGAGCTAAA

GGCAGACTGGCCACCTAGGTGCCCCCCATATACTATATTCTTAAAGTAAGCTAGAGATAA

TGCTATTAATATAATCACAAGGAAAAAAAGAAACATTCACAATACTGTTACTGTATTTAT

CCAAAAAACCCATGTATAAGTGGTCCTACACAGTTCAATCCTGTGTTTTCTAAGGGTCAA

CTGAATTTATTTCTTACCAACATATATATGTGAAAGTATGAAAGACAGATAAAAAGAATT

AAAACTTACACTCATGAGACTGGGGGTACCATCAAAGCGACCTTATTTGATTAATGAAGA

AAAATTTAAAAAGATGGTATTCATACATTATTTGTTTATTATAATATGAACATGACAAAA

TGTCAAGTGTTATAATTTCCTCTAGCTTCAAGAATAATCAACATTCTGCCTGTATTGTCT

CCCCTATACTTCTAGTGGCTCCGCAGAAGCCCACAGATATTTTTGAGTTACAATCTATGT

AAGTTGAAATGCACAATCTTAATTTTGCCATTTAGATAACAAAGTTTGACACATTTTGAT

ATAACCTTGTCACCCAGCCCTACCAAGCATAAGACACTGCTGCCATGTTTCCTTCTGAAA

GTTAATATATTTTATGCTTAGATCTACTATCCATCTCAAATTGTGTATAGTATGAGGTAG

GGGTCAGAATCCACTCAGCTCTGCTTGTTTCAGCACTCTCTTCAGACTTTCCTTCCCCCA

TGGATTCATTTTGGAGCTTTTGTAGAAAACCATTTGATTACACTTATGTGTCTTTTCCTG

GGCTCTATTCAGTTCATGGTTGTTGTCTTATGGTAGCTTTACAGAATTCTTGAAGTCAGC

TAGTTTAAGACCTCCAGCTTTGTACTACACTTTCATTTTGGAATTGCTTGTCAGTTTCTA

CAAACAAAACCAGCTAGGATTTTGATGGGGATCAAATGAAATCATCTTGGAGTACTGACA

TCTCAACACTATTGAGTGTTCCATCTATGAATATGGTACTGTTACTTAAATCTTCCGGGC

AGCCCGGGTGGCTCAGCGGTTTGGCACTGCCTTCGGCCTGGGGTGTGGTTCTGGGGACCA

GGGATCGAGTCCCACGCAGGCTCCCTGCGTGGGGCCTGCTTCTTCCTCTGCCTGTGTCTC

TGCCTCTCACTTTGTGTCTCTCATTAATAAACAAATAAAATAAAATAAAATAAAAATCTT

CCTTAAATTCGCTCAGCAATATTTTGCAGTTTCTAACATAGAACTCTTGTCCGTCTTTCT

TCAATTTATCCCTGGGTATGCCATGTTTTTGGATGCTTTGTAAATGCCACTGCTTTTATA

ATTCATTTTCCGACTTCAGATTCAATGGTTGTTATTACATAAAAATACAATTTATGTATA

TTGCCTTTGTAACCTGTGACCTTGCTATAATCCTTTATCAGTCATGATAGTTCTTCTTTT

TAAGAGGTTTCATTTTTTAAGTAATCTCTGCATCCAAGGTGGGCCTCAAATTTACAACTC

TGAGATCAAGAGTCATGTATTCTACTGACTGAGCCAACCAGGTGCCCCTTGTGAGTTTTT

CAATGCTTCTTCTATTAGCCTGGTTAACACCTTCAGTATAATGTTGAAGGGGTGAGAATC

AAATCCTTTCCTAGTTCCTCATCTTAGAAAACAAGCATTCAGTCTGTCACTGTTACGTAT

TGATGGTAACTTGATTGGGGGTGCTCTTTATCAGACTAAGTTTTCCCTTTCCTAGTTTGC

TGGGAGGTTGGCTTTTCTTTTCTTTCTTTTTTTAAAATCATGAATAGGTGTTGCGTGATG

TCAACTGCTTTTTATGTATCTGTTGAAATGATCATAGGATTTTTATCCTTCAATCTGTTT

ATGTGGCAAATTACACTGATTTTTTGAATGTTAAAACATTGCCGTCTAGAGATAAACTCC

ACTTGATTAGGATGTATTTATCCGTTTTATAGATAATCAGATTTACTTTGCTAAGATTTG

TGAGATTTTTGCATATATACATATGTGTGTATATATACACACATATGTATATATGCTGGC

AGGAATATTGGTCTAAATGTTTTCTTGTAACATTCTCGTCTGGTTTTGATATGAGGATTC

TCAGGATATCATGAAATTAGTTGGGAAGATGTTACCTCTTCTACATTCTGAAAGTTTTTG

TAAGATTGTTATTGTTTCTTCCTCAAGTGTCTGAAGAGAAGAAGAAATAGAATAGAAAAT

AGCATTAACACAGTAGACTTAAACCCAAACCTTACAATAATTGCATTAAATTAAATAGAC

CAAGCACTCCAATTAAAAGTGATCCAGTGAGGGGGGAAGGAGGGCTGTTTCAACAAGTGG

CACTAGAAGAACTGGATATACATCTAGGAAAACCCCAAAAGAAAACAGAACCCCTACCTC

ACATCACGATAAAAATTACTCTTAATTAATTAGTTAATTAATTATGGCTCAAAGACCTAA

ATATAAAACCTAAAACCCTAAAGATTCTGGAAGAAAATATCCTAGAATATCTTCATGATC

TTGGGGTCAGCAAGGATTTTCTAAACACACAGAACACATTTGAAAGACAGAAGATAAATC

TGCTGCTGCTCTGCCTTAGAGGCTGACAGAGAGGCAAGAAACAGGTCAGGAGAAAATAGA

TACTGACAAAAGACTCCTATCCGGAATATATAAAGAACTGCATGCAGCTGAACGCAAGTT

AAAATGGCCAATAAGCACGTGGAAACTGATCACAGCTAAGAGAATTGCAACTGAAAACCA

CAATGATGCACCACATTAGAAAGTCTGACAAAACCAGATGTGAGGAAAGACAGCTGTTGC

TGGTGGGTACATAAAACAGAACAACCACTTTGAAATATACTAATTTTAAAAATCAAATTT

AGATCTCTCTCACCTAGCAAATTCCACTCCCAGACGGTTATAAAAAACAAAAACAAAACA

AAACAAAACACACACACACACAAACACCCAAAACCAACCCCATGAAAACATAGGACCACA

AAGAGAATTGTACAAGAATGTTAATAGCAGCTTTATTCAAAATAACCCCGAACGGTTAAC

AACCCAAGCGGTCACCGAAAGGAACATGGATTAAAAAAAACCTCACGGTTTATTCATCCA

GTGAGATGTGCTAAATACAAAAATACAAAGGTGCAAAAAACACTGATACACTACTGATAC

AGTAGTACTGGTTACACAGGAACAAGGATGGGTGTCACAGGTATTATGCTGCCGGCGAGA

AGCCTACACAAAGGAGCACACACTATGTGGGTCTATTTACACGACGCTCTCTTCAACAGG

CACAAGTAGTGACAGAACTCCAAGATGAGGGTCAGAGACCCCCAGAGGGGAGAGGCCCAA

GAACCTTCTCTGGGGCGATGACAATGTGTAAATTGTGTCATTTGACAGGCTGCTGGTTAG

CAGCCCAAACCTGTCTAATTGCAGTGAGTGTAAATGCTACCTCAATTTCAGAACAAGCCG

CACACAAGCACCGCGTGCACTGGCCGGTACCGAGCAGGTGCACAGGTAGGTGAGCGAGCG

CAGGCTGGAAGCACCGCGCGCCCGGAGTGACTGACAGGCGGGAGGCGGTATCCCGTCCCC

TCGGCTCGGCTCGGCTCGGCTCGGCTCGGCGGGCCCCGCCCCCTCAAGCCCCGCCCCTTC

CGCCCTCACGACGTTCGCTCTGCGTCGGAACTCGGAATGGCTGAGTCCCGAGCCGGATGC

CAGAGAAACCGTGTCTCGGCCCCACACAAACGGCGCATCGTCGCATTCTGCCATCGTTGT

CATTCTCAAATGTACTCGTGCTCGAAAATACCCGGGACTTGCCGTCGGCACGCTTCTGGA

GACCTCGTCCCTCGCCCGCCCAGATTGCAGGCGATGGGGTGGGGCGCACCATTCAAGGAG

TGGGGGAAGGCTCGAGGTTTGCATCCAAACAGACCCTCCCTCCCGGCCTGCCAAAAGTAG

CAGCTAAGGAGTTCAGGGGAAAAAAAAAAAAAAAAAAAAAAAAAAAGAAAGAAAAGAAAA

AATAAGAAAGAAAAAAGAAAAGAAAAGAAAAGAAAAGAAAGCCAGTAGAGGAGGCGGCGC

GCAGCCTCCCTGAGCACGTCAGTCTAAATGACACTTTCCCTCTCCTCGGGGGAACAGTGA

ACTGCTCCGGAAAAAAAACCGGAAACGCTGCGGCAAGGGGCAGAAATGTAAATGTTCGAC

CAAACAATAACAGAGCTCGCGGGCCTCCTGGACCCGGACCGTCCCGCCGGGCGTCGCGGG

CAGACTGCTCGTGGAGCGCTACCCACGTCCACGCCAGGCAGTCTACTAGGATCGTCTCCA

TTTAGTGGAGCCAGGGGCCGCTGGGGAGATCCCTCCGGGAAGGACAAAGGTCCGGAAGTT

CTGGGACCTGAGCGGCTTGGGCTTCCTGGATCGACCCCCCCGAATGTGATCATGTCGGGA

CGGAGCCCGAGAGGACCCTCCAGGGATGCTGCGGGCTCGAAGATCTCGGGGGTCCCCAAA

ATCCCGAGATCGGGGAAAAAACAATCTCGGATCCTGATGCTTTGTGCGTCTTATTTCGGG

GTTCTTCACACCCGGCACGGGGAGAGAAAGGGAGGGGAAGGGTGAGGAGGAACCCCTTCG

GCCGGCTGCACGTCCCCCTCCTATAAAGCTGAGTCCGGCTGCTCCCAACAGCCCCTTCAT

CTGCACCCAGGCCTCCCAGCGGTGCATTCCGATATTGGTGCCGCCAGCCAAACTTCCATT

CCCCCGCGATCGCTGATCGCGTGCCCCACCCCCAGACCCCAATTCTCCCCAAGCTCAAAA

CACACAGAGCCGAGAGCCGGTGACTCAGACAGGACTCATCTGGCTCAGTCAGGAGCTTAC

CCGAACCTCAGGGGAGCGTGTCACCGTCCTGGGAAGCACGCTCCGAGGCCGGACGCCAGA

GGATCCCGGAGCCCGGCGGCTACCTGCGCCCAGGATGGTGGCTCTGGACTTTTGAGAAGC

TCAAAACTTTTAGCGCCAGTGAGTCCGAGCACATGGGAGGGG
