# Supplementary material for: Primer-walking sequencing identifies predominantly noncoding TP53 variants in canine cancers
Source: J Vet Sci. 2026 May 27;27(4):e42. doi: 10.4142/jvs.25325 (PMC13454805; doi:10.4142/jvs.25325)
Supplement: Supplementary file 3 [file jvs-27-e42-s003.doc]

**Supplementary Data 3.** >Sequence of ROS_Cfam_1.0:5:32665394:32678755:1_forward_4617 bp

TGGTAGTAAAGTTTTATTGTAAAATAAGAGATTGATATAAAAACGGGATATAAAAAGGGA

AGAGGAGGAGGGGTGGGGGTGATCAATGCATGAATGCAGATGTGCCTCACAGAGTGCAAA

AGAAATTGATCCTGAGCAATTGGGGGCGGGGGGGGGGGGGAAGCAGTAAAAGCCCTTGGT

AGACAAAGATCCTACACCAGGGATGGGATTCTGCAGGGTGTGAGATGGGAGTGAGATTTC

ATCTTAGGTGCAGGGGTCTCCCAAAAAGGCTGGACAGGATTTGGCCAGGGCAGGGAATGA

GCACCCACACAGGCACTAAGTCACAGACATGGGATAAAAGGTGGTTTTGGGGTCAGACAG

ACATTATTTCATTCATCCTCACAAGCTCAGTGAGGTAGGTATGGTTAATTAGCAATTCAT

AAAAATGGCCTGTGAAATTAGGAAAAATTCAGCCACTGGCTACAATGATAGAGAACTGGG

CTCATGACTCAGTGTCTTAGGGGCTATAAAATGCTCCCAACATTCAGGAACATTTTCCTA

CCTCTCCCATGGTTCTACACAGTAAAAAACTTAAAAACCAGGCTGGAACGTCCTTTATCT

CCCGACCTACCAACTGTGCAGGCCAACTTCTTTAGTAGAGACTTACGGGAAAGGCAAATG

GAAGAATCTAGGGTGTTTCTGAGGCACATCTGTACCATGCAAAGTAACACACCGGAATAG

GTGTGCTCAAGCTCCCAATTCCAAAAATGCCAGGGAAGGAGGACGAGAGTGAGGGTGTGC

GTGTTGGGGGAGGACAGGAAGCAGGGGAAGTCAGTCTGAGTCGGGCCCTTCTCTCTTGAA

CATCAGTTTTTTATGGCGAGAGGTAGATTGCCCCTTCTTTGCCTTCAGGTGGCTGTTGGG

GACACAAGGTGGAGGGGAAGAGACGATATTAGCAAGTGGGAGCCTCGGGGCTCCTTGTCC

TAGATCTAGGCTTGGAAGGGTCCAAGGCTGGAGTAAGGGAATTTTTTTTTTTTTTTTTTT

TTTGGAATGAGGTAATTTAAGTTAAAATGCTGTAGACACATTTACTGAGCACCCAGGGAA

CTTCTGTGTGTTCGCTTCCTCTCCCCTCACCCCATCTGATGAACAAGGAAACCGAGCCCC

AGAAGGGGAGATGCAAGGCCACACGGATCTGCTCATCCTTGGCAGGGCTGGGGGTCTGGC

CCAATCCTGCTGTGTCCAGTATTTTCCCACACGGTTATCAGGTCCCTATTGGAGACCCCA

CTTGGCACTAGGGGTTGCTGGAGATGACAAACACCCATGCTTGTCCCTTAAGGGGTTTGG

GGACCTCCCGAGGCAGAAAAGGGAGTACAGGATCCAACTGGGGGTATGGTAGAAGCAGGT

TTGCCCAGGGTGGGACCAACCATGAGGGGATCAGTTCCCTTCCACCTTGAAGGTAGGAGT

AGAATGGAATCCTGTGGCTTTCCAACATATGAAGGCAAGGGGGTGGGGCTGCAAGCCGGC

CCAGGTCACTCACCTGGAGTGAGCCCTGCTTCCCCCTGGCTCCTTTCCACTCTGGGCATC

CTTCAGCTCCAAGGCTTCATTCAGATTCCTGAACATCTCATAGCGTTCACGCCCACGGAT

CTGTGGCAGTGGGGGGGAGGAGGAGAAAGAATAAGAGGGGGAAGCCACAGTACCATTTAA

CTCAGACTAAGGTGTAAGTGCCAATCTGCAGCAAGGCACAGCCCGCAGCCACTGTCAAGG

CACCGCTGCCCCTTGATGGCAAGTGTGCTAATTGCAGGCGGAGCAGTCAAAAGAAAACTG

TGCTCTTGGCCCTCAGCTTGAAACAAATCTTCAATCCAAGAATATCAACTTCAAGAATCA

GGGACAGGACCAGAGAAAAGGATGGAGAGAGGAACAGGCCTAGCCTCACATGTGCTCCAT

TCACATTTTTTTCTTCCCTCATCTGATCCCGCTAAGCGGGGGCAAGAGCCTGCCTTATCT

GTTCCTCCCAGCCTTGGTACCTGAAGGGTGAAATATTCTCCATCTAGTGGCTTCTTCTTT

TGCGGGGGAGAGGAGCTGGTGCTGGGAGGCAGTGCTGGGAAAGAGAGGAGGAAAAGACAA

GAGAAGAGTATGTTTTGAGCAGAATTGCACCCTCTGTATCTGCCCCACATCCTCCTCTTT

TTTTTTTTTTTTTCAAGATTATATTTATTTATTCATGAGACATACACACAAAGGCAAAAA

AATAGGCAGAGGGAGAGGCAGGCTCCCTACAGGGACCCTGACACGGGACTCGATCCCAGG

ACCCTGGGATCATGCCTGATGCTCAACCCCTGAGCCACCCAGGTGTCCCTCCTTCACCTC

CTCTTGTCTTGCTTGCTTACCTCGCTTGGTACTCCCGGGGGGTGGCTCAGGACAAGGCTC

CCCCTTCTTGTGGAAATTCTCCTCCTCAGTCCGGCGGTCTCTCCCGGGACAGGCACAAAC

GCGTACCTCAAAGCTGTTGCGTCCCAGCACGTTTCCACTACCCAGGTGAGAAGAGAGAAG

CAGGGGGCCGTGAGGGTGGTGGGCCCCACTGACTCCTAGCCACCCTCATCCTACGTAGAG

CCAAAGCCTACAGACTATTGAGAAGCCACAGGTCAGGGAGGTCCCAAAGCCAGAGGAAAG

AAAACCAGGGGAGGCGGATGGCAGAGGAGATTCCACATTGGGACAGGAATGGATGGGAAG

GATGGAGGGGGGTACAGTAAGGAAGTGGCCAGGAGCAGGGTGGCAGGCAGGTCCCTACCT

GGAGTCTTCCAGGGTGATGATAGTGAGGATGGGCCGCCGGTTCATGCCTCCCATGCAGGA

ACTGTTACACATGTAGTTGTAGTGGATGGTGGTATAGTCAGAGCCAACCTGAGGTAGAAG

GTAGGCCCAGGGTCGGGCCACTCAGCCCTGGGCAGGCCTGTAGTCTATCAGCCTCAGTCT

TTCCCAACCGCAGAATGGGATGACCCACCTGCCTCTGCACTCCTCACAGGGCTGCGGGGA

GGTTGAAGGAGGAGAAAGCCCCCTCAACTCCCGCCTCACAAATCCCTCCAGCCCCCTCCT

CCCAGAGACCCCTCAGATGCCAAACCAGACCTCGGGTGGCTCATAAGGCACCACCACACT

GTGTCGAAAAGTGTTTCTGTCGTCCAGGTACTTGGCCCGCAAATTTCCTTCCACTCGGAT

GAGATGCTGAGGAGGGGCAAGACCTAAGAGCCATCGGGGAGGAATCAGGGCTTTGGGCCC

TCTGGGGAGCAGCCTTGTCCCATCTGTAGCCCCCGATGGCTTACCGTCACTACTGTCAGA

GCAGCGTTCATGGTGGGGGCAGCGCCGCACAACCTCGGTCACGAACTCCGACTTCTTATA

GATGGCCATAGCGCGGACGCAGGTATTGGGTGGGGGTGGGGAGCTGACCCACAGCTGCAC

GGGGCAGGTCTTCGCCAGCTGGCAAAACAACTTGTTGAGGAGAGGGGAGTACTGTAAGAA

AAGGACAGATGGACAGGTCAGGGCATGTGTGCCCGGGGTGGGTGGTGAGAAGTAGAGGGG

TCGGGGTCTATTCCAGAAGCTTACTTTATTTTAGACACACAGAGAGAGGCAGAGACACAG

GCAGAGGGAGAAGTGGTATGGGATTCGATCCCAGGACCTGGGGCTCACACCCTGAACCAA

AGGCAGACGCTCAACTGCTGAGCTGCCCAGGCATCTCCCAGAAGCTTACTTTAAAAGCCA

AGGAATGAACAAGTGAAGGAAAATGAAGAAGAGTCCCTTTGCCCCATGACAGGAAAGGTG

AAAGCCCAAGGTCAAGGGGGGAAATGTCAACTTTGACACAGAGCTGAGGCCAAAGGTCAC

AGAATACATAGGTAATGCAGGAAGACTGACAGTGCGGACCGAGAGGAGTCTCAAAATCCC

AAACAGAGAAACCCAGGGGCCACAGCCAGGCCCCGGCCAGCCCCATGGAAACCCGCCTCG

CAGGGCCACTGACCGTCCAAGTAACAGACTTGGCTGTCCCGGAATGCAGGAACCCCAAAC

GGAACCCATAGGTGCCAGGGTAGGTCTTCGGGGAAGGGACAGAGGATGATAGGGGCCAGG

AGGGGGCCGGTCCAGGGGCTGTGGGGGCAGAAGTGGCTGGCATCCTGGGAGCATCATCTG

AGTCTTCGTCTAGCCAGTTCACGACGCTCTCTGGGAGCAGCAGCTCATCCACTGCTGGGC

ACAGCTCCGAAGACTGTAGGGGGCGAGACCAGAGTCAAGGGTCAGGTCCCTAGGTCCCCA

GCTCCCAACCCCCGCCCTGGACCTCCCCACACCCAGTCCTTACCAGAACATTGTTTTCAG

GAAGCCTGGAAGATGAGAGCAGAAAGTCAGTGCTGGTTTTGCTTCCTCTGGGCTTCTACT

TGGGGGGGCGGGGGGGGGGGCTGGGGCTGGGGGTTGGGATTGGGGTGGGTGGGCAGGGGT

CCCAGAGGGCTGGTGGGGAGCCTGGCCTGCCCTCCACTTACAGGTTCCACAATTCTGAAA

ATGTCTCCTGGCTCAGAGGGGGGTCGATATTGAGCTCTGACTGTGGCTCTTGCATCGCAG

CTCCTGGGAAGGGAGTGTGGGTGCTGTGGAGGGCAAGGCGAGGGGTCAGCGGGAGAC
