# Supplementary material for: Primer-walking sequencing identifies predominantly noncoding TP53 variants in canine cancers
Source: J Vet Sci. 2026 May 27;27(4):e42. doi: 10.4142/jvs.25325 (PMC13454805; doi:10.4142/jvs.25325)
Supplement: Supplementary file 4 [file jvs-27-e42-s005.doc]

**Supplementary Data 5.** ROS_Cfam_1.0_Forward-Whole length-Genbank file

LOCUS	5 13362 bp DNA HTG 12-OCT-2025

DEFINITION Canis lupus familiaris primary_assembly 5 ROS_Cfam_1.0 partial sequence

32665394..32678755 reannotated via EnsEMBL

ACCESSION	primary_assembly:ROS_Cfam_1.0:5:32665394:32678755:1

VERSION	primary_assembly:ROS_Cfam_1.0:5:32665394:32678755:1

KEYWORDS	.

SOURCE	dog

ORGANISM Canis lupus familiaris

Eukaryota; Opisthokonta; Metazoa; Eumetazoa; Bilateria;

Deuterostomia; Chordata; Craniata; Vertebrata; Gnathostomata;

Teleostomi; Euteleostomi; Sarcopterygii; Dipnotetrapodomorpha;

Tetrapoda; Amniota; Mammalia; Theria; Eutheria; Boreoeutheria;

Laurasiatheria; Carnivora; Caniformia; Canidae.

COMMENT	This sequence was annotated by Ensembl (www.ensembl.org). Please visit the Ensembl

or EnsemblGenomes web site, http://www.ensembl.org/ or

http://www.ensemblgenomes.org/ for more information.

COMMENT	All feature locations are relative to the first (5') base of the sequence in this

file. The sequence presented is always the forward strand of the assembly.

Features that lie outside of the sequence contained in this file have clonal

location coordinates in the format: .:..

COMMENT	The /gene indicates a unique id for a gene, /note="transcript_id=..." a unique id

for a transcript, /protein_id a unique id for a peptide and note="exon_id=..." a

unique id for an exon. These ids are maintained wherever possible between

versions.

COMMENT	All the exons and transcripts in Ensembl are confirmed by similarity to either

protein or cDNA sequences.

FEATURES	Location/Qualifiers

source	1..13362

/organism="Canis lupus familiaris"

/db_xref="taxon:9615"

gene	complement(1..13362)

/gene=ENSCAFG00845024647.1

/locus_tag="TP53"

/note="tumor protein p53 [Source:NCBI gene (formerly

Entrezgene);Acc:403869]"

mRNA	join(complement(13201..13362),complement(4482..4583),

complement(4304..4325),complement(3974..4213),

complement(3285..3471),complement(3091..3203),

complement(2759..2868),complement(2361..2497),

complement(2001..2074),complement(1514..1620),

complement(1..892))

/gene="ENSCAFG00845024647.1"

/standard_name="ENSCAFT00845043577.1"

CDS	join(complement(4482..4555),complement(4304..4325),

complement(3974..4213),complement(3285..3471),

complement(3091..3203),complement(2759..2868),

complement(2361..2497),complement(2001..2074),

complement(1514..1620),complement(811..892))

/gene="ENSCAFG00845024647.1"

/protein_id="ENSCAFP00845034138.1"

/note="transcript_id=ENSCAFT00845043577.1"

/db_xref="EntrezGene_trans_name:TP53-202"

/db_xref="RefSeq_peptide:NP_001376147"

/db_xref="RefSeq_mRNA:NM_001389218"

/db_xref="Uniprot/SPTREMBL:A0A8C0RLV2"

/db_xref="Uniprot/SPTREMBL:E7FIY6"

/db_xref="EMBL:AB537893"

/db_xref="EMBL:KJ511265"

/db_xref="GO:0000122"

/db_xref="GO:0000122"

/db_xref="GO:0000423"

/db_xref="GO:0000785"

/db_xref="GO:0000976"

/db_xref="GO:0000976"

/db_xref="GO:0000976"

/db_xref="GO:0000977"

/db_xref="GO:0000978"

/db_xref="GO:0000978"

/db_xref="GO:0000981"

/db_xref="GO:0000987"

/db_xref="GO:0001046"

/db_xref="GO:0001094"

/db_xref="GO:0001223"

/db_xref="GO:0001227"

/db_xref="GO:0001228"

/db_xref="GO:0001228"

/db_xref="GO:0001666"

/db_xref="GO:0001701"

/db_xref="GO:0001756"

/db_xref="GO:0001836"

/db_xref="GO:0002020"

/db_xref="GO:0002039"

/db_xref="GO:0002244"

/db_xref="GO:0002309"

/db_xref="GO:0002326"

/db_xref="GO:0002360"

/db_xref="GO:0002931"

/db_xref="GO:0003677"

/db_xref="GO:0003677"

/db_xref="GO:0003677"

/db_xref="GO:0003677"

/db_xref="GO:0003682"

/db_xref="GO:0003682"

/db_xref="GO:0003700"

/db_xref="GO:0003700"

/db_xref="GO:0003700"

/db_xref="GO:0003700"

/db_xref="GO:0003730"

/db_xref="GO:0005507"

/db_xref="GO:0005515"

/db_xref="GO:0005634"

/db_xref="GO:0005634"

/db_xref="GO:0005634"

/db_xref="GO:0005634"

/db_xref="GO:0005634"

/db_xref="GO:0005654"

/db_xref="GO:0005657"

/db_xref="GO:0005667"

/db_xref="GO:0005730"

/db_xref="GO:0005737"

/db_xref="GO:0005737"

/db_xref="GO:0005737"

/db_xref="GO:0005739"

/db_xref="GO:0005739"

/db_xref="GO:0005739"

/db_xref="GO:0005759"

/db_xref="GO:0005759"

/db_xref="GO:0005783"

/db_xref="GO:0005813"

/db_xref="GO:0005813"

/db_xref="GO:0005829"

/db_xref="GO:0005829"

/db_xref="GO:0005856"

/db_xref="GO:0006289"

/db_xref="GO:0006302"

/db_xref="GO:0006355"

/db_xref="GO:0006355"

/db_xref="GO:0006355"

/db_xref="GO:0006355"

/db_xref="GO:0006357"

/db_xref="GO:0006357"

/db_xref="GO:0006606"

/db_xref="GO:0006914"

/db_xref="GO:0006915"

/db_xref="GO:0006915"

/db_xref="GO:0006915"

/db_xref="GO:0006915"

/db_xref="GO:0006974"

/db_xref="GO:0006974"

/db_xref="GO:0006979"

/db_xref="GO:0006983"

/db_xref="GO:0007179"

/db_xref="GO:0007265"

/db_xref="GO:0007346"

/db_xref="GO:0007369"

/db_xref="GO:0007405"

/db_xref="GO:0007406"

/db_xref="GO:0007417"

/db_xref="GO:0007507"

/db_xref="GO:0007623"

/db_xref="GO:0008104"

/db_xref="GO:0008156"

/db_xref="GO:0008283"

/db_xref="GO:0008285"

/db_xref="GO:0008285"

/db_xref="GO:0008340"

/db_xref="GO:0009299"

/db_xref="GO:0009303"

/db_xref="GO:0009410"

/db_xref="GO:0009411"

/db_xref="GO:0009651"

/db_xref="GO:0009792"

/db_xref="GO:0010165"

/db_xref="GO:0010332"

/db_xref="GO:0010332"

/db_xref="GO:0010628"

/db_xref="GO:0010629"

/db_xref="GO:0010659"

/db_xref="GO:0010666"

/db_xref="GO:0012501"

/db_xref="GO:0014009"

/db_xref="GO:0016032"

/db_xref="GO:0016363"

/db_xref="GO:0016604"

/db_xref="GO:0016605"

/db_xref="GO:0016605"

/db_xref="GO:0017053"

/db_xref="GO:0019661"

/db_xref="GO:0019899"

/db_xref="GO:0021549"

/db_xref="GO:0030308"

/db_xref="GO:0030330"

/db_xref="GO:0030330"

/db_xref="GO:0030512"

/db_xref="GO:0030971"

/db_xref="GO:0031571"

/db_xref="GO:0031571"

/db_xref="GO:0031625"

/db_xref="GO:0031625"

/db_xref="GO:0032211"

/db_xref="GO:0032991"

/db_xref="GO:0033077"

/db_xref="GO:0033209"

/db_xref="GO:0033554"

/db_xref="GO:0034103"

/db_xref="GO:0034644"

/db_xref="GO:0034644"

/db_xref="GO:0035033"

/db_xref="GO:0035264"

/db_xref="GO:0035794"

/db_xref="GO:0035861"

/db_xref="GO:0036310"

/db_xref="GO:0042127"

/db_xref="GO:0042149"

/db_xref="GO:0042771"

/db_xref="GO:0042771"

/db_xref="GO:0042802"

/db_xref="GO:0042826"

/db_xref="GO:0042981"

/db_xref="GO:0043065"

/db_xref="GO:0043065"

/db_xref="GO:0043066"

/db_xref="GO:0043066"

/db_xref="GO:0043073"

/db_xref="GO:0043153"

/db_xref="GO:0043504"

/db_xref="GO:0043516"

/db_xref="GO:0043523"

/db_xref="GO:0043525"

/db_xref="GO:0045815"

/db_xref="GO:0045861"

/db_xref="GO:0045892"

/db_xref="GO:0045892"

/db_xref="GO:0045893"

/db_xref="GO:0045893"

/db_xref="GO:0045899"

/db_xref="GO:0045930"

/db_xref="GO:0045944"

/db_xref="GO:0045944"

/db_xref="GO:0046677"

/db_xref="GO:0046872"

/db_xref="GO:0046982"

/db_xref="GO:0048144"

/db_xref="GO:0048147"

/db_xref="GO:0048147"

/db_xref="GO:0048511"

/db_xref="GO:0048512"

/db_xref="GO:0048539"

/db_xref="GO:0048568"

/db_xref="GO:0050821"

/db_xref="GO:0051087"

/db_xref="GO:0051262"

/db_xref="GO:0051262"

/db_xref="GO:0051276"

/db_xref="GO:0051402"

/db_xref="GO:0051721"

/db_xref="GO:0051726"

/db_xref="GO:0051726"

/db_xref="GO:0060218"

/db_xref="GO:0060253"

/db_xref="GO:0060333"

/db_xref="GO:0060411"

/db_xref="GO:0061629"

/db_xref="GO:0062100"

/db_xref="GO:0065003"

/db_xref="GO:0070059"

/db_xref="GO:0070242"

/db_xref="GO:0070243"

/db_xref="GO:0070245"

/db_xref="GO:0070266"

/db_xref="GO:0071456"

/db_xref="GO:0071466"

/db_xref="GO:0071479"

/db_xref="GO:0071479"

/db_xref="GO:0071480"

/db_xref="GO:0071480"

/db_xref="GO:0071494"

/db_xref="GO:0072089"

/db_xref="GO:0072331"

/db_xref="GO:0072331"

/db_xref="GO:0072332"

/db_xref="GO:0072332"

/db_xref="GO:0072593"

/db_xref="GO:0072717"

/db_xref="GO:0090200"

/db_xref="GO:0090398"

/db_xref="GO:0090399"

/db_xref="GO:0090403"

/db_xref="GO:0097252"

/db_xref="GO:0097371"

/db_xref="GO:0097718"

/db_xref="GO:0140296"

/db_xref="GO:0140677"

/db_xref="GO:0140693"

/db_xref="GO:1900119"

/db_xref="GO:1901525"

/db_xref="GO:1902108"

/db_xref="GO:1902253"

/db_xref="GO:1902749"

/db_xref="GO:1902895"

/db_xref="GO:1903451"

/db_xref="GO:1903799"

/db_xref="GO:1904024"

/db_xref="GO:1905856"

/db_xref="GO:1990144"

/db_xref="GO:1990841"

/db_xref="GO:2000269"

/db_xref="GO:2000378"

/db_xref="GO:2000379"

/db_xref="GO:2000647"

/db_xref="GO:2000772"

/db_xref="GO:2000774"

/db_xref="GO:2000774"

/db_xref="GO:2001244"

/db_xref="HGNC_trans_name:TP53-201"

/db_xref="protein_id:AHZ62767"

/db_xref="protein_id:BAJ72203"

/db_xref="UniParc:UPI000016C14B"

/translation="MQEPQSELNIDPPLSQETFSELWNLLPENNVLSSELCPAVDELL

LPESVVNWLDEDSDDAPRMPATSAPTAPGPAPSWPLSSSVPSPKTYPGTYGFRLGFLH

SGTAKSVTWTYSPLLNKLFCQLAKTCPVQLWVSSPPPPNTCVRAMAIYKKSEFVTEVV

RRCPHHERCSDSSDGLAPPQHLIRVEGNLRAKYLDDRNTFRHSVVVPYEPPEVGSDYT

TIHYNYMCNSSCMGGMNRRPILTIITLEDSSGNVLGRNSFEVRVCACPGRDRRTEEEN

FHKKGEPCPEPPPGSTKRALPPSTSSSPPQKKKPLDGEYFTLQIRGRERYEMFRNLNE

ALELKDAQSGKEPGGSRAHSSHLKAKKGQSTSRHKKLMFKREGPDSD"

mRNA	join(complement(4482..4555),complement(3974..4166),

complement(3285..3471),complement(3091..3203),

complement(2759..2868),complement(2361..2497),

complement(2001..2074),complement(1514..1620),

complement(1..892))

/gene="ENSCAFG00845024647.1"

/standard_name="ENSCAFT00845043646.1"

CDS	join(complement(4482..4555),complement(3974..4166),

complement(3285..3471),complement(3091..3203),

complement(2759..2868),complement(2361..2497),

complement(2001..2074),complement(1514..1620),

complement(811..892))

/gene="ENSCAFG00845024647.1"

/protein_id="ENSCAFP00845034194.1"

/note="transcript_id=ENSCAFT00845043646.1"

/db_xref="EntrezGene_trans_name:TP53-201"

/db_xref="Uniprot/SPTREMBL:A0A8C0NMX3"

/db_xref="Uniprot/SPTREMBL:A0A8I3PMM3"

/db_xref="GO:0000976"

/db_xref="GO:0000976"

/db_xref="GO:0003677"

/db_xref="GO:0003677"

/db_xref="GO:0003677"

/db_xref="GO:0003700"

/db_xref="GO:0003700"

/db_xref="GO:0005515"

/db_xref="GO:0005634"

/db_xref="GO:0005634"

/db_xref="GO:0005634"

/db_xref="GO:0005737"

/db_xref="GO:0005739"

/db_xref="GO:0005759"

/db_xref="GO:0005783"

/db_xref="GO:0005813"

/db_xref="GO:0005856"

/db_xref="GO:0006355"

/db_xref="GO:0006355"

/db_xref="GO:0006915"

/db_xref="GO:0006915"

/db_xref="GO:0006915"

/db_xref="GO:0012501"

/db_xref="GO:0016605"

/db_xref="GO:0046872"

/db_xref="GO:0048511"

/db_xref="GO:0051262"

/db_xref="GO:0051262"

/db_xref="HGNC_trans_name:TP53-202"

/db_xref="UniParc:UPI001236EEBC"

/translation="MQEPQSELNIDPPLSQETFSELWNLVVNWLDEDSDDAPRMPATS

APTAPGPAPSWPLSSSVPSPKTYPGTYGFRLGFLHSGTAKSVTWTYSPLLNKLFCQLA

KTCPVQLWVSSPPPPNTCVRAMAIYKKSEFVTEVVRRCPHHERCSDSSDGLAPPQHLI

RVEGNLRAKYLDDRNTFRHSVVVPYEPPEVGSDYTTIHYNYMCNSSCMGGMNRRPILT

IITLEDSSGNVLGRNSFEVRVCACPGRDRRTEEENFHKKGEPCPEPPPGSTKRALPPS

TSSSPPQKKKPLDGEYFTLQIRGRERYEMFRNLNEALELKDAQSGKEPGGSRAHSSHL

KAKKGQSTSRHKKLMFKREGPDSD"

gene	5:32678578..32690834

/gene=ENSCAFG00845024805.1

/locus_tag="WRAP53"

/note="WD repeat containing antisense to TP53

[Source:UniProtKB Gene Name;Acc:A0A8I3PST8]"

mRNA	join(5:32678578..32680354,5:32680514..32680609,

5:32680882..32680993,5:32688506..32688594,

5:32688795..32688885,5:32688993..32689125,

5:32689688..32689896,5:32690107..32690210,

5:32690351..32690485,5:32690572..32690834)

/gene="ENSCAFG00845024805.1"

/standard_name="ENSCAFT00845043832.1"

CDS	join(5:32679954..32680354,5:32680514..32680609,

5:32680882..32680993,5:32688506..32688594,

5:32688795..32688885,5:32688993..32689125,

5:32689688..32689896,5:32690107..32690210,

5:32690351..32690485,5:32690572..32690752)

/gene="ENSCAFG00845024805.1"

/protein_id="ENSCAFP00845034349.1"

/note="transcript_id=ENSCAFT00845043832.1"

/db_xref="RefSeq_mRNA_predicted:XM_038665443"

/db_xref="RefSeq_peptide_predicted:XP_038521371"

/db_xref="Uniprot/SPTREMBL:A0A8I3PST8"

/db_xref="Uniprot/SPTREMBL:A0A8P0NI36"

/db_xref="GO:0000781"

/db_xref="GO:0003723"

/db_xref="GO:0003723"

/db_xref="GO:0003723"

/db_xref="GO:0005515"

/db_xref="GO:0005634"

/db_xref="GO:0005654"

/db_xref="GO:0005694"

/db_xref="GO:0005697"

/db_xref="GO:0005829"

/db_xref="GO:0006281"

/db_xref="GO:0006974"

/db_xref="GO:0007004"

/db_xref="GO:0015030"

/db_xref="GO:0015030"

/db_xref="GO:0015030"

/db_xref="GO:0016604"

/db_xref="GO:0030576"

/db_xref="GO:0030576"

/db_xref="GO:0031625"

/db_xref="GO:0032203"

/db_xref="GO:0032212"

/db_xref="GO:0034337"

/db_xref="GO:0035861"

/db_xref="GO:0042393"

/db_xref="GO:0042802"

/db_xref="GO:0044877"

/db_xref="GO:0045739"

/db_xref="GO:0051087"

/db_xref="GO:0070034"

/db_xref="GO:0090666"

/db_xref="GO:0090671"

/db_xref="GO:0140597"

/db_xref="GO:0140691"

/db_xref="GO:0140691"

/db_xref="GO:1904851"

/db_xref="GO:1904867"

/db_xref="GO:1905168"

/db_xref="GO:2000781"

/db_xref="GO:2001034"

/db_xref="HGNC_trans_name:WRAP53-201"

/db_xref="Uniprot_gn_trans_name:WRAP53-201"

/db_xref="UniParc:UPI0000EB3FED"

/translation="MSAPEAPRSAPDCLAADQAPAPAPLSPRASPTGESPDSELLPPP

VPPAGDGPARSFPDPAAGSRAPGEGGPVSASTALQAGFSAPTDLSPRIEEPEVGENVS

LPAEEADGPELGPGEAVEGVSEDLAPEDEGYSTRNYCFSQLPRFLSGSWSEFTQPENF

LKGCKWAPDGSCILTNSADNILRIYNLPPELYNEGEQVEYAEMVPVLRMVEGDTIYDY

CWYSLMSSSEPDTSYVASSSRENPIHIWDAFTGELRASFRAYNHLDELTAAHSLCFSP

DGSQLFCGFNRTIRVFSTARPGRDCEVRATFAKKQGQSGIISCIAFSPAQPLYACGSY

SRSLGLYACDDGSPLALLGGHQGGITHLCFHPDGNRFFSGARKDAELLCWDLRQPSHP

LWSLSREVTTNQRIYFDLDSTGQFLVSGSTSGAVSVWDTGGAGQEGKPEPLLSFLPQK

DCTNGVSLHPSLPLLATASGQRVFPEPTESEDEGEREVDLPLLSMRHVHLECQLQLWW

CGGDMEPG"

exon	complement(1..892)

/note="exon_id=ENSCAFE00845204719.1"

exon	complement(2001..2074)

/note="exon_id=ENSCAFE00845204630.1"

exon	complement(3091..3203)

/note="exon_id=ENSCAFE00845204576.1"

exon	complement(2361..2497)

/note="exon_id=ENSCAFE00845204608.1"

exon	complement(13201..13362)

/note="exon_id=ENSCAFE00845204505.1"

exon	complement(1514..1620)

/note="exon_id=ENSCAFE00845204671.1"

exon	complement(3285..3471)

/note="exon_id=ENSCAFE00845204556.1"

exon	complement(4482..4555)

/note="exon_id=ENSCAFE00845205042.1"

exon	complement(3974..4166)

/note="exon_id=ENSCAFE00845205082.1"

exon	complement(2759..2868)

/note="exon_id=ENSCAFE00845204595.1"

exon	complement(3974..4213)

/note="exon_id=ENSCAFE00845204542.1"

exon	complement(4482..4583)

/note="exon_id=ENSCAFE00845204516.1"

exon	complement(4304..4325)

/note="exon_id=ENSCAFE00845204534.1"

exon	5:32680514..32680609

/note="exon_id=ENSCAFE00845205707.1"

exon	5:32678578..32680354

/note="exon_id=ENSCAFE00845205688.1"

exon	5:32690351..32690485

/note="exon_id=ENSCAFE00845206052.1"

exon	5:32688993..32689125

/note="exon_id=ENSCAFE00845205901.1"

exon	5:32688506..32688594

/note="exon_id=ENSCAFE00845205764.1"

exon	5:32690107..32690210

/note="exon_id=ENSCAFE00845206010.1"

exon	5:32680882..32680993

/note="exon_id=ENSCAFE00845205709.1"

exon	5:32689688..32689896

/note="exon_id=ENSCAFE00845205955.1"

exon	5:32688795..32688885

/note="exon_id=ENSCAFE00845205830.1"

exon	5:32690572..32690834

/note="exon_id=ENSCAFE00845206089.1"

gene	complement(811..4560)

/gene=

misc_RNA	join(complement(4482..4560),complement(4304..4325),

complement(3974..4213),complement(3285..3471),

complement(3091..3203),complement(2759..2868),

complement(2361..2497),complement(2001..2074),

complement(1514..1620),complement(811..892))

/gene=""

/note="cDNA"

/standard_name="NM_001003210.1"

gene	1..545

/gene=

misc_RNA	1..545

/gene=""

/note="cDNA"

/standard_name="CO691075.1"

gene	1..629

/gene=

misc_RNA	1..629

/gene=""

/note="cDNA"

/standard_name="CO683288.1"

gene	complement(1066..2078)

/gene=

misc_RNA	join(complement(2001..2078),complement(1066..1620))

/gene=""

/note="cDNA"

/standard_name="DN756500.1"

gene	complement(3175..13304)

/gene=

misc_RNA	join(complement(13201..13304),complement(4482..4583),

complement(4304..4325),complement(3974..4213),

complement(3285..3471),complement(3175..3203))

/gene=""

/note="cDNA"

/standard_name="DN444655.1"

gene	5:32678354..32678808

/gene=

misc_RNA	5:32678354..32678808

/gene=""

/note="cDNA"

/standard_name="DN354659.1"

gene	complement(1525..3995)

/gene=

misc_RNA	join(complement(3974..3995),complement(3285..3471),

complement(3091..3203),complement(2759..2868),

complement(2361..2497),complement(2001..2074),

complement(1525..1620))

/gene=""

/note="cDNA"

/standard_name="AF159062.1"

gene	complement(811..4555)

/gene=

misc_RNA	join(complement(4482..4555),complement(4304..4325),

complement(3974..4213),complement(3285..3471),

complement(3091..3203),complement(2759..2868),

complement(2361..2497),complement(2001..2074),

complement(1514..1620),complement(811..892))

/gene=""

/note="cDNA"

/standard_name="KP279763.1"

gene	complement(811..4555)

/gene=

misc_RNA	join(complement(4482..4555),complement(4304..4325),

complement(3974..4213),complement(3285..3471),

complement(3091..3203),complement(2759..2868),

complement(2361..2497),complement(2001..2074),

complement(1514..1620),complement(811..892))

/gene=""

/note="cDNA"

/standard_name="KP279761.1"

gene	complement(811..4555)

/gene=

misc_RNA	join(complement(4482..4555),complement(4304..4325),

complement(3974..4213),complement(3285..3471),

complement(3091..3203),complement(2759..2868),

complement(2361..2497),complement(2001..2074),

complement(1514..1620),complement(811..892))

/gene=""

/note="cDNA"

/standard_name="KP279764.1"

gene	complement(811..4555)

/gene=

misc_RNA	join(complement(4482..4555),complement(4304..4325),

complement(3974..4213),complement(3285..3471),

complement(3091..3203),complement(2759..2868),

complement(2361..2497),complement(2001..2074),

complement(1514..1620),complement(811..892))

/gene=""

/note="cDNA"

/standard_name="KP279762.1"

gene	complement(829..4025)

/gene=

misc_RNA	join(complement(3974..4025),complement(3285..3471),

complement(3091..3203),complement(2759..2868),

complement(2361..2497),complement(2001..2074),

complement(1514..1620),complement(829..892))

/gene=""

/note="cDNA"

/standard_name="L37107.1"

gene	complement(2..13328)

/gene=

misc_RNA	join(complement(13201..13328),complement(4482..4583),

complement(4304..4325),complement(3974..4213),

complement(3285..3471),complement(3091..3203),

complement(2759..2868),complement(2361..2497),

complement(2001..2074),complement(1514..1620),

complement(2..892))

/gene=""

/note="cDNA"

/standard_name="AB537893.1"

gene	complement(2058..4325)

/gene=

misc_RNA	join(complement(4304..4325),complement(3974..4213),

complement(3285..3471),complement(3091..3203),

complement(2759..2868),complement(2361..2497),

complement(2058..2074))

/gene=""

/note="cDNA"

/standard_name="S77819.1"

gene	complement(811..4560)

/gene=

misc_RNA	join(complement(4482..4560),complement(4304..4325),

complement(3974..4213),complement(3285..3471),

complement(3091..3203),complement(2759..2868),

complement(2361..2497),complement(2001..2074),

complement(1514..1620),complement(811..892))

/gene=""

/note="cDNA"

/standard_name="AF060514.1"

gene	complement(2..13328)

/gene=gene-TP53.1

/locus_tag="TP53"

/note="tumor protein p53 [Source:NCBI gene;Acc:403869]"

mRNA	join(complement(13201..13328),complement(4482..4583),

complement(4304..4325),complement(3974..4213),

complement(3285..3471),complement(3091..3203),

complement(2759..2868),complement(2361..2497),

complement(2001..2074),complement(1514..1620),

complement(2..892))

/gene="gene-TP53.1"

/standard_name="rna-NM_001389218.1.1"

CDS	join(complement(4482..4555),complement(4304..4325),

complement(3974..4213),complement(3285..3471),

complement(3091..3203),complement(2759..2868),

complement(2361..2497),complement(2001..2074),

complement(1514..1620),complement(811..892))

/gene="gene-TP53.1"

/protein_id="cds-NP_001376147.1.1"

/note="transcript_id=rna-NM_001389218.1.1"

/db_xref="EntrezGene:403869"

/db_xref="EntrezGene:403869"

/db_xref="VGNC:VGNC:47719"

/db_xref="VGNC:VGNC:47719"

/db_xref="GenBank:NM_001389218.1"

/db_xref="GenBank:NP_001376147.1"

/translation="MQEPQSELNIDPPLSQETFSELWNLLPENNVLSSELCPAVDELL

LPESVVNWLDEDSDDAPRMPATSAPTAPGPAPSWPLSSSVPSPKTYPGTYGFRLGFLH

SGTAKSVTWTYSPLLNKLFCQLAKTCPVQLWVSSPPPPNTCVRAMAIYKKSEFVTEVV

RRCPHHERCSDSSDGLAPPQHLIRVEGNLRAKYLDDRNTFRHSVVVPYEPPEVGSDYT

TIHYNYMCNSSCMGGMNRRPILTIITLEDSSGNVLGRNSFEVRVCACPGRDRRTEEEN

FHKKGEPCPEPPPGSTKRALPPSTSSSPPQKKKPLDGEYFTLQIRGRERYEMFRNLNE

ALELKDAQSGKEPGGSRAHSSHLKAKKGQSTSRHKKLMFKREGPDSD"

gene	5:32677789..32690832

/gene=gene-WRAP53.1

/locus_tag="WRAP53"

/note="WRAP53 [Source:NCBI gene;Acc:607892]"

mRNA	join(5:32677789..32680354,5:32680514..32680609,

5:32680882..32680993,5:32688506..32688594,

5:32688795..32688885,5:32688993..32689125,

5:32689688..32689896,5:32690107..32690210,

5:32690351..32690485,5:32690572..32690732,

5:32690735..32690832)

/gene="gene-WRAP53.1"

/standard_name="rna-XM_038665443.1.1"

CDS	join(5:32679954..32680354,5:32680514..32680609,

5:32680882..32680993,5:32688506..32688594,

5:32688795..32688885,5:32688993..32689125,

5:32689688..32689896,5:32690107..32690210,

5:32690351..32690485,5:32690572..32690732,

5:32690735..32690817)

/gene="gene-WRAP53.1"

/protein_id="cds-XP_038521371.1.1"

/note="transcript_id=rna-XM_038665443.1.1"

/db_xref="EntrezGene:607892"

/db_xref="EntrezGene:607892"

/db_xref="VGNC:VGNC:48432"

/db_xref="VGNC:VGNC:48432"

/db_xref="GenBank:XM_038665443.1"

/db_xref="GenBank:XP_038521371.1"

/translation="MSAPEAPRSAPDCLAADQAPAPAPLSPRASPTGESPDSELLPPP

VPPAGDGPARSFPDPAAGSRAPGEGGPVSASTALQAGFSAPTDLSPRIEEPEVGENVS

LPAEEADGPELGPGEAVEGVSEDLAPEDEGYSTRNYCFSQLPRFLSGSWSEFTQPENF

LKGCKWAPDGSCILTNSADNILRIYNLPPELYNEGEQVEYAEMVPVLRMVEGDTIYDY

CWYSLMSSSEPDTSYVASSSRENPIHIWDAFTGELRASFRAYNHLDELTAAHSLCFSP

DGSQLFCGFNRTIRVFSTARPGRDCEVRATFAKKQGQSGIISCIAFSPAQPLYACGSY

SRSLGLYACDDGSPLALLGGHQGGITHLCFHPDGNRFFSGARKDAELLCWDLRQPSHP

LWSLSREVTTNQRIYFDLDSTGQFLVSGSTSGAVSVWDTGGAGQEGKPEPLLSFLPQK

DCTNGVSLHPSLPLLATASGQRVFPEPTESEDEGEREVDLPLLSMRHVHLECQLQLWW

CGGHGTRIADAPQGQKGQGGTEESECEFT"

exon	complement(2001..2074)

/note="exon_id="

exon	complement(4482..4560)

/note="exon_id="

exon	complement(811..892)

/note="exon_id="

exon	complement(1514..1620)

/note="exon_id="

exon	complement(3091..3203)

/note="exon_id="

exon	complement(3974..4213)

/note="exon_id="

exon	complement(3285..3471)

/note="exon_id="

exon	complement(4304..4325)

/note="exon_id="

exon	complement(2361..2497)

/note="exon_id="

exon	complement(2759..2868)

/note="exon_id="

exon	1..545

/note="exon_id="

exon	1..629

/note="exon_id="

exon	complement(1066..1620)

/note="exon_id="

exon	complement(2001..2078)

/note="exon_id="

exon	complement(3175..3203)

/note="exon_id="

exon	complement(4482..4583)

/note="exon_id="

exon	complement(3285..3471)

/note="exon_id="

exon	complement(3974..4213)

/note="exon_id="

exon	complement(4304..4325)

/note="exon_id="

exon	complement(13201..13304)

/note="exon_id="

exon	5:32678354..32678808

/note="exon_id="

exon	complement(2759..2868)

/note="exon_id="

exon	complement(1525..1620)

/note="exon_id="

exon	complement(2361..2497)

/note="exon_id="

exon	complement(3285..3471)

/note="exon_id="

exon	complement(3974..3995)

/note="exon_id="

exon	complement(2001..2074)

/note="exon_id="

exon	complement(3091..3203)

/note="exon_id="

exon	complement(3091..3203)

/note="exon_id="

exon	complement(811..892)

/note="exon_id="

exon	complement(1514..1620)

/note="exon_id="

exon	complement(2001..2074)

/note="exon_id="

exon	complement(3285..3471)

/note="exon_id="

exon	complement(3974..4213)

/note="exon_id="

exon	complement(2361..2497)

/note="exon_id="

exon	complement(4304..4325)

/note="exon_id="

exon	complement(4482..4555)

/note="exon_id="

exon	complement(2759..2868)

/note="exon_id="

exon	complement(4304..4325)

/note="exon_id="

exon	complement(2361..2497)

/note="exon_id="

exon	complement(4482..4555)

/note="exon_id="

exon	complement(2759..2868)

/note="exon_id="

exon	complement(3091..3203)

/note="exon_id="

exon	complement(2001..2074)

/note="exon_id="

exon	complement(811..892)

/note="exon_id="

exon	complement(1514..1620)

/note="exon_id="

exon	complement(3974..4213)

/note="exon_id="

exon	complement(3285..3471)

/note="exon_id="

exon	complement(2759..2868)

/note="exon_id="

exon	complement(4482..4555)

/note="exon_id="

exon	complement(4304..4325)

/note="exon_id="

exon	complement(2361..2497)

/note="exon_id="

exon	complement(3974..4213)

/note="exon_id="

exon	complement(3285..3471)

/note="exon_id="

exon	complement(3091..3203)

/note="exon_id="

exon	complement(2001..2074)

/note="exon_id="

exon	complement(811..892)

/note="exon_id="

exon	complement(1514..1620)

/note="exon_id="

exon	complement(3091..3203)

/note="exon_id="

exon	complement(2001..2074)

/note="exon_id="

exon	complement(811..892)

/note="exon_id="

exon	complement(1514..1620)

/note="exon_id="

exon	complement(3974..4213)

/note="exon_id="

exon	complement(3285..3471)

/note="exon_id="

exon	complement(4304..4325)

/note="exon_id="

exon	complement(2361..2497)

/note="exon_id="

exon	complement(2759..2868)

/note="exon_id="

exon	complement(4482..4555)

/note="exon_id="

exon	complement(2759..2868)

/note="exon_id="

exon	complement(2361..2497)

/note="exon_id="

exon	complement(3974..4025)

/note="exon_id="

exon	complement(829..892)

/note="exon_id="

exon	complement(3285..3471)

/note="exon_id="

exon	complement(3091..3203)

/note="exon_id="

exon	complement(1514..1620)

/note="exon_id="

exon	complement(2001..2074)

/note="exon_id="

exon	complement(2..892)

/note="exon_id="

exon	complement(2361..2497)

/note="exon_id="

exon	complement(4304..4325)

/note="exon_id="

exon	complement(13201..13328)

/note="exon_id="

exon	complement(2759..2868)

/note="exon_id="

exon	complement(1514..1620)

/note="exon_id="

exon	complement(2001..2074)

/note="exon_id="

exon	complement(3091..3203)

/note="exon_id="

exon	complement(4482..4583)

/note="exon_id="

exon	complement(3285..3471)

/note="exon_id="

exon	complement(3974..4213)

/note="exon_id="

exon	complement(3974..4213)

/note="exon_id="

exon	complement(3285..3471)

/note="exon_id="

exon	complement(2058..2074)

/note="exon_id="

exon	complement(3091..3203)

/note="exon_id="

exon	complement(2759..2868)

/note="exon_id="

exon	complement(4304..4325)

/note="exon_id="

exon	complement(2361..2497)

/note="exon_id="

exon	complement(3285..3471)

/note="exon_id="

exon	complement(3974..4213)

/note="exon_id="

exon	complement(3091..3203)

/note="exon_id="

exon	complement(4482..4560)

/note="exon_id="

exon	complement(1514..1620)

/note="exon_id="

exon	complement(811..892)

/note="exon_id="

exon	complement(2001..2074)

/note="exon_id="

exon	complement(2759..2868)

/note="exon_id="

exon	complement(2361..2497)

/note="exon_id="

exon	complement(4304..4325)

/note="exon_id="

exon	complement(2759..2868)

/note="exon_id=exon-NM_001389218.1-7.1"

exon	complement(2..892)

/note="exon_id=exon-NM_001389218.1-11.1"

exon	complement(13201..13328)

/note="exon_id=exon-NM_001389218.1-1.1"

exon	complement(3974..4213)

/note="exon_id=exon-NM_001389218.1-4.1"

exon	complement(4482..4583)

/note="exon_id=exon-NM_001389218.1-2.1"

exon	complement(4304..4325)

/note="exon_id=exon-NM_001389218.1-3.1"

exon	complement(2001..2074)

/note="exon_id=exon-NM_001389218.1-9.1"

exon	complement(3091..3203)

/note="exon_id=exon-NM_001389218.1-6.1"

exon	complement(2361..2497)

/note="exon_id=exon-NM_001389218.1-8.1"

exon	complement(1514..1620)

/note="exon_id=exon-NM_001389218.1-10.1"

exon	complement(3285..3471)

/note="exon_id=exon-NM_001389218.1-5.1"

exon	5:32680882..32680993

/note="exon_id=exon-XM_038665443.1-3.1"

exon	5:32689688..32689896

/note="exon_id=exon-XM_038665443.1-7.1"

exon	5:32690107..32690210

/note="exon_id=exon-XM_038665443.1-8.1"

exon	5:32688795..32688885

/note="exon_id=exon-XM_038665443.1-5.1"

exon	5:32677789..32680354

/note="exon_id=exon-XM_038665443.1-1.1"

exon	5:32680514..32680609

/note="exon_id=exon-XM_038665443.1-2.1"

exon	5:32690351..32690485

/note="exon_id=exon-XM_038665443.1-9.1"

exon	5:32690572..32690732

/note="exon_id=exon-XM_038665443.1-10.1"

exon	5:32690735..32690832

/note="exon_id=exon-XM_038665443.1-11.1"

exon	5:32688506..32688594

/note="exon_id=exon-XM_038665443.1-4.1"

exon	5:32688993..32689125

/note="exon_id=exon-XM_038665443.1-6.1"

mRNA	join(complement(5370..5512),complement(4482..4583),

complement(4304..4325),complement(3974..4213),

complement(3285..3471),complement(3091..3203),

complement(2759..2868),complement(2361..2497),

complement(2001..2074),complement(1514..1620),

complement(5:32663928..32663979))

/product="MRWWQRSLVQRQGPDWDLPSDLLAPGDTSQGAPGFGTIHLLTTVSPTS

THTPFPGAAMQEPQSELNIDPPLSQETFSELWNLLPENNVLSSELCPAVDELLLPESV

VNWLDEDSDDAPRMPATSAPTAPGPAPSWPLSSSVPSPKTYPGTYGFRLGFLHSGTAK

SVTWTYSPLLNKLFCQLAKTCPVQLWVSSPPPPNTCVRAMAIYKKSEFVTEVVRRCPH

HERCSDSSDGLAPPQHLIRVEGNLRAKYLDDRNTFRHSVVVPYEPPEVGSDYTTIHYN

YMCNSSCMGGMNRRPILTIITLEDSSGNVLGRNSFEVRVCACPGRDRRTEEENFHKKG

EPCPEPPPGSTKRALPPSTSSSPPQKKKPLDGEYFTLQIRGRERYEMFRNLNEALELK

DAQSGKEPGGSRAHSRMWGPVADMAPAHKFQT"

/note="identifier=.1"

/note="Derived by automated computational analysis using

gene prediction method:genscan"

mRNA	join(11794..11865,5:32678605..32678819,

5:32679540..32679597,5:32680191..32680354,

5:32680514..32680609,5:32680882..32680993,

5:32688506..32688594,5:32688795..32688885,

5:32688993..32689125,5:32689688..32689896,

5:32690107..32690210,5:32690351..32690485,

5:32690572..32690729,5:32692936..32693071,

5:32695293..32695585,5:32695803..32695895,

5:32695987..32696091,5:32696544..32696947,

5:32698528..32698717,5:32699980..32700079,

5:32700462..32700578,5:32704209..32704624,

5:32706530..32706712,5:32709151..32709212,

5:32710831..32711001,5:32718643..32718871,

5:32719195..32719305,5:32719461..32719699,

5:32720638..32720829,5:32721455..32721612,

5:32722061..32722148,5:32722487..32722692,

5:32723132..32723261,5:32723507..32723689,

5:32724495..32724709,5:32735192..32735338,

5:32736149..32736305,5:32736760..32736999,

5:32737269..32737457,5:32737658..32737805,

5:32738265..32738457,5:32740711..32740911,

5:32741000..32741156,5:32741520..32741694,

5:32742090..32742256,5:32743261..32743422,

5:32743525..32743628,5:32745195..32745351,

5:32745492..32745574,5:32745690..32745870,

5:32746254..32746392,5:32747990..32748051,

5:32748328..32748429,5:32748758..32748883,

5:32749176..32749286,5:32749525..32749713,

5:32750076..32750179,5:32750960..32751162,

5:32751849..32751976,5:32752289..32752441,

5:32752693..32752869,5:32753604..32753822,

5:32754082..32754199,5:32754427..32754563,

5:32755034..32755171,5:32755258..32755416,

5:32757890..32758042,5:32758469..32758560,

5:32758821..32759019,5:32759154..32759378,

5:32760172..32760276,5:32761438..32761626,

5:32761786..32761871,5:32761953..32762083,

5:32762179..32762328,5:32762617..32762784,

5:32762998..32763117,5:32763409..32763604,

5:32764939..32765071,5:32765175..32765314,

5:32766579..32766789,5:32766975..32767169,

5:32767250..32767391,5:32768028..32768197,

5:32768304..32768420,5:32777466..32777630,

5:32778133..32778258,5:32778356..32778504,

5:32778618..32778684,5:32778802..32778952,

5:32779088..32779236,5:32779418..32779551,

5:32779698..32779842,5:32779945..32780144,

5:32780858..32780984,5:32783106..32783292,

5:32783446..32783594,5:32783760..32783943,

5:32784176..32784366,5:32784860..32784965,

5:32785212..32785243,5:32789074..32789265,

5:32789426..32789610,5:32789838..32790066,

5:32790145..32790296,5:32790429..32790522,

5:32790993..32791169,5:32791268..32791342,

5:32791925..32792075,5:32792201..32792285,

5:32793631..32793832,5:32794792..32794896,

5:32801742..32801906,5:32803614..32803669,

5:32805132..32805272,5:32805700..32805798,

5:32805901..32806120,5:32806204..32806296,

5:32806384..32806545,5:32806630..32806824,

5:32806917..32807282,5:32807396..32809593,

5:32809691..32809817,5:32809947..32810052,

5:32810789..32810994,5:32811110..32811174,

5:32811259..32811330,5:32811419..32811567,

5:32811731..32811845,5:32811927..32812114,

5:32812271..32812412,5:32812665..32812791,

5:32812887..32813057,5:32813154..32813177)

/product="MLPQFQNKPHTSTACTGRYRAGAQGSVSPSWEARSEAGRQRIPEPGGY

LRPGWWLWTFEKLKTFSASESEHMGGENPNPSNPREAPGTKLDSRHDKGSRTTTPSNH

HEPYGLMGTAGFSAPTDLSPRIEEPEVGENVSLPAEEADGPELGPGEAVEGVSEDLAP

EDEGYSTRNYCFSQLPRFLSGSWSEFTQPENFLKGCKWAPDGSCILTNSADNILRIYN

LPPELYNEGEQVEYAEMVPVLRMVEGDTIYDYCWYSLMSSSEPDTSYVASSSRENPIH

IWDAFTGELRASFRAYNHLDELTAAHSLCFSPDGSQLFCGFNRTIRVFSTARPGRDCE

VRATFAKKQGQSGIISCIAFSPAQPLYACGSYSRSLGLYACDDGSPLALLGGHQGGIT

HLCFHPDGNRFFSGARKDAELLCWDLRQPSHPLWSLSREVTTNQRIYFDLDSTGQFLV

SGSTSGAVSVWDTGGAGQEGKPEPLLSFLPQKDCTNGVSLHPSLPLLATASGQRVFPE

PTESEDEGEREVDLPLLSMRHVHLECQLQLWWCGLGGVMGALRSGPGGVRVGALLLLG

ALGLVSGLSLEPVYWNSANKRFQAEGGYVLYPQIGDRLDLLCPRARPPGPHSSPNYEF

YKLYLVGGAQGRRCEAPPAPNLLLTCDRPDLDLRFTIKFQEYSPNLWGHEFRSHHDYY

IIATSDGTREGLESLQGGVCLTRGMKVLLRVGQSPRGGAAPRKPVSEMPMERDRGAAH

GLEPGKDNMPGDPTSNATSRGAEGPLPPPSMPAVAGAAGGLALLLLGVAGAGGAMCWR

RRRAKPSESRHPGPGSFGRGGSLGLGSGSGMGPREAEPGELGIALRGGGAADPPFCPH

YEKVSGDYGHPVYIVQDGPPQSPPNIYYKLCSIRSELPLGTKSATSITSQDGYFVHQR

SNHLSGTVAPSSFLILLITPSRGQEVDIAMGEVAASRVDGADSPIAGYQPQGLGNRHY

GNPEPSNRAQLCEGTGDLHTEQHMPPHAPSGYKYANMFPGEQRQHNAHGTQPLFSRRT

EPLPGPVTFPRESPPPVPPKTGDASQSRSPGSPKFQFRSSLASPAPRSPVLRDPGPGA

DEGTGGRARVLQPRFFPRTQLRPRLPRNPGAPSRAQTGPAGGTPGATCRTRGERARRR

GRGVGRAGLAGRPGSAHTMSSKAEKKQKVSAWGSGSRGGARKRAAQAALASQAAETTL

PSGEPEVEPEPLKEELEPTLEETVVEKEELQEPEVDVKPLFLSRAVLTGLVDAVWTAE

HDAVLEHFARDPSELVLTIFIDPYAGLKLELSMPVQPQNQIVYFIRCAPVPITTENFE

ATVQFGTMRGSYIPALLRLLSGVFAPQIFTNTTWPESIRNHFASHLHRFLACLTDTRY

KLEGHTVLYIPAEAMNMAPEVVVRDKELVQRLETSMIHWTRQIKEVLSAQESVETGEN

LGPLEEIEFWHNRCMDLSGISKQLVKQGVKHIESILRLAKSSYLAPFMKLAQQIQDGS

RQAQSNLTFLSILKEPYQELAYMRPKDISSKLPKLISLIRIIWVNSPHYNTRERLTSL

FRKMSNEIIRLCCHAISLDRIFEGHVTSSKEELQGCIACCQAWKDHYLRAVQMHTQFS

SRGWVLDQTSIFAQVDAFVQRCKDLIEVCDCQYHFARWEDGKQAPLPCFFGAQGPQIT

RNLLEIEDIFYKNLQMLRAVRGGILDVKNTSWHEDYNRFRTGVKDLEVMTQNLITSAF

ELVRDVEQGVLLLDTFHRLATREAIKRTYDKKAVDLYMLFNSELALVNREVNKKWPYL

EPYMAQYSGQAHWMRILRHRIDRVMNCLSSAHFLPHIGTGEESVHTYQQMVQAIDELV

RKTFQDWTLTLDKDCIRRLDTPLLRISQEKAGMLDVNFDKSLLILFVEIDYWERLLFE

TPHYVMNVAERAEDLRILRENLLLVARDYNRIIAMLSPDEQALFKERIRFLDKKIHPG

LKKLHWALKGASAFFITECRIHASKVQTIVNEFKASTLTIGWRAQEISETLLVHISNK

RVYRDLEFEEDQQEHRTAVQQKLMSLHQDVVAIMTNSYEVFKNDGPEIQQQWMLYTIR

LDHMMEDALRLNVKWSLLELSKAINGDGKTTPNPLFQVLVILQNDTQGGVAQVEFSPT

LQTLAGVVNDIGHHLFSTISVFRHLPEILIKRKFHRDPIHVIVERDEDIKKIQAQISS

GVTNNASLLQNYLKTWDLYREIWEINKDSFIRRYQRLNPPVSSFDADIARYTEVANNV

QKEETVLNIQFVLLDCSHLKFSLVQHCNEWQNKFTTLLKEMAARRLLELHTYLRDNAE

KISHPPQTLEELGVSLQLMETLQHDLPNLETQIPPIHEQFTILEKYEVLVQENVLEML

DSLNGEWVIFQQILLDSEQMLKKHKDKFKTGLIHSADDFKKKAHNLLEDFESKGPFTS

NVGYQTALEQIAQVRAMLNAMQEEENSLRSNLGIFKIEQPASKDLQNLEKVELDALQQ

VWEITRDWEENWNQWKIGQFLTLQTEAMETMAHGLFRRLTRLAKEYKDRNWEIIETTR

SKIEQFKRIMPVISDLRNPALRERHWDQIRDEIQREFDQESESFTLEQIVDLGRDQHV

EKIGEISASATKELAIELALQNIAKTWKVIQLDIVPYKDKGHHRLRGTEEVFQALEDN

QVALSTMKASHFVKAFEKDVDHWERCLSLILEVVEMVLTVQRQWMYLENIFLGEDIRK

QLPSESALFDQVNSNWKTIMDRMSKDSNALRSTHHPEAVQPHLKKCFDNIKLLRIQKV

GGPSSKWEAVGMFSGDGEYIDFLHPVLLEGPVESWLGDVERTMRVTLRDLLRNCRLAL

KKFLNKRDKWVKEWAGQMVITASQIQWTADVTKCLLTAKERGDKKILKVMKKKQVSVL

NKYSEAIRGNLTKIMRLKIVALVTIEVHARDVLEKLYKSGLMDMSSFDWLSQLRFYWE

KDLDDCMIRQTNTQFQYSYEYLGNSGRLVITPLTDSDSASHPQTGAWGCFDEFNRINV

EVLSVVAQQILSILSALAASLTRFYFEGFEINLVWSCGIFITMNPGYAGRTELPDNLK

SMFRPIAMVVPDSTLIAEIILFGEGFGNCKILAKKVYTLYSLAVQQLSRQDHYDFGLR

ALTSLLRYAGKKRRLQPDLSDEEVLLLSMRDMNIAKLTSGDMPLFNAIVQDLFPNVEL

PVIDYGKVSAPRYPHRPHMGMWVQLRETIEQEIHDMGLQSTLFTLTKVIQLYETKNSR

HSTMIVGCTGSGKTTSWRVLQSSLSNLCRAGEPNFNIVREFPLNPKALSLGELYGEYD

LNTNEWTDGVLSSVMRTACADEKPDEKWILFDGPVDTLWIESMNSVMDDNKVLTLING

ERIAMPEQVSLVFEVENLAMASPATVSRCGMVYTDYTDLGWKPYVQSWLDKRPKTEVE

PLQRMFEKFINKMLAFKKDNCTELVPLPEYSGIVSLCKLYSALATPENGVNPADGENY

ISMVELTFVFSMIWSVCASVDEEGRKKIDSYLREIEGSFPNKDTVYEYFVDPKMRNWT

SFEDKLPKSWRYPPNSPFYKIMVPTVDTVRYNYLVSTLVANQNPILLVGPVGTGKTSI

AQSVLQSLPSIQWSVLTVNMSAQTTSNNVQSIIESRVEKRTKGVYVPFGGKSMITFMD

DLNMPAKDTFGSQPPLELIRLWIDYGFWYDRSKQTIKYIRDMFLMAAMGPPGGGRTVI

SPRLQSRFNIINMTFPTESQIIQIFGTMINQKLQDFEEEVKPIGNVVTEATLDVYNTV

VQRFLPTPAKIHYLFNLRDISKVFQGMLRANKDFHDTKSSITRLWIHECFRVFSDRLV

DATDMEAFMAIISDKLGSFFDLTFHNLCPSRRSPIFGDFMREPKVYEDLTDLGVLKAA

METALKEYNLSPAVVPMQLVLFREAIEHITRIVRVIGQPRGNMLLVGIGGSGRQSLAR

LASSICEYITFQIEVTKHYRKQEFREDIKRLYRQAGVELKATSFLFVDTQIADESFLE

DINNILSSGLCHWKWRSPSSAHVALVQIQMLIAEQAKADQVSQSSDSLFTYLIERVRN

NLHIVLCLSPVGDPFRNWIRQYPALVNCTTINWFSEWPREALLEVAEKYLVGVDLGTQ

ENIHKKVAKIFVTMHWSVAQYSQKMLLELRRHNYVTPTNYLELVSGYKKLLGEKRQEL

LDQANKLRTGLFKIDETREKVEVMSLELEDAKKKVAEFQKQCEEYLVIIVQQKREADE

QQKAVTANSEKIAIEEVKCQTLADNAQKDLEEALPALEEAMRVSEQEGQGREWDQSSS

SFKSQGKWEEALESLNKKDIGEIKSYGRPPAQVEIVMQAVMILRGNEPTWAEAKRQLG

EQNFIKSLIHFDKDNISDKVLKKIGAYCAQPDFQPDIIGRVSLAAKSLCMWVRAMELY

GRLYRVVEPKRIRMNAALAQLQEKQAALAEAQEKLREVAEKLEMLKKQYDEKLAQKEE

LRKRSEEMEVKLERAGLLVSGLAGEKARWEETVQGLEKDLGYLVGDCLLAAAFLSYMG

PFLTNYRDEIVNQIWMRKISELQVPCSPRFTFDNFLSNPTKVRDWNIQGLPSDAFSTE

NGIIVTRGNRWALMIDPQAQALKWIKNMEGNQGLQIIDLQMRDYLQVLEKAIQFGYPV

LLQNVQEYLDPTLNPVLNKSVARIGGRLLMRIGDKEVEYNTNFRFYITTKLSNPHYSP

ETSAKTTIVNFAVKEQGLEAQLLGIVVRKERPELEEQKDSLVINIAAGKRKLKELEDE

ILRLLNEATGSLLDDVQLVNTLRTSKMTATEVTEQLETSETTEINIDMAREAYRPCAQ

RASVLFFVLNDMGRIDPMYQFSLDAYIGLFILSIDKSHRSNKLEDRIDYLNEYHTYAV

YRYTCRTLFERHKLLFSFQICAKILETSGKLNMDEYNFFLRGGVVLDREGQMDNPCTS

WLADAYWDNITELDKLTNFHGLMNSFEQYPRDWHLWYTNATPEKAMLPGEWENTCNEM

QRMLIVRSLRQDRVAFCVTSFIVSNLGSRFIESPVLNMKLVMEDSTPRTPLVFILSPG

VDPTSALLQLAEHTGMAQRFHALSLGQGQAPIAARLLREGVIQGHWVFLANCHLSLSW

MPNLDKLVEQLQVEDPHPSFRLWLSSSPHPDFPISILQASIKMTTEPPKKKKKKKKKK

KKKKKKKKKKKKKEEEEEEGRKGKNSTKIKPRPYTACGLKANMTRLYQLMTEPQFSHC

SKPAKYKKLLFALCFFHSVLLERKKFLQLGWNIIYGFNDSDFEVSENLLSLYLDEYEE

TPWDALKYLIAGVNYGGHVTDDWDRRLLTTYINDYFCDQALSTPFYRLSVLEAYVIPK

DGSLASYKEYISMLPSMDPPEAFGQHPNADVASQITEARTLFETLLSLQPQITPTRVG

GQSREEKVLELATDVKQKIPEMIDYEGTRKLLAMDPSPLNVVLLQEIQRYNKLLETIL

SSLTDLEKGIQGLIVMSTSLEEIFNCIFDAHAYPSQKPLASWTRDLAMRVEQFELWAS

RARPPVIFWLSGFTFPTGFLTAVLQSSARQNNIPVDNLSWEFIVSTVDDSNLVYPPKD

GVWVRGLYLEGAGWDRKNSCLVEAEPMQLVCLMPTIHFRPTESRKKSAKGMYSCPCYY

YPNRAGSSERASFVIGIDLRPPALASRSIPGPTPLGRSSPGPEPSAVRTFAAQPRDGE

GETRGPNRGSRVGAVAGRAAAAPARPEGQEERTGYRRPFPGCSHSGSAAVGGGEDAAG

KPPAARVRSERVTPRFHRAECGLAGSGTRRDPGPAPPRRVCLPDYGLPGRPRRRGPVG

GRHTLNGPIPGAEPAPYLGDAGVVCERPQRPSGGSGHSCQSSWSGPSPPRAGAGGRAR

SPGLLGPLLTWCSASIGQPPLSAPLPPSHGSSSGHPNKPYYAPGTPTPRPLHGKLESL

HGCVQALLREPAQPGLWEQLGQLYESEHDSEEAIRCYHSALRYGGSLAELGPRIGRLQ

QAQFWNFHAGSCQHRAKVLPPLEQVWNLLHLEHKRSYGAKRGGPPVKRAAEPPVVQPV

PPAALSGPSGDEGLSPGGKRRRGCSSEQTGLPPGLPLPPPPLPPPPPPPPPPPPLPGL

ATSPPFQLTKPGLWSTLHGDVWGPDRKGSVPPERQEQRHPLPHPYPYPVPAYTTHPPG

HRLVPAAPPGPGPRPPGAESHGCPPATCPPGSDLRESRVQRSRMDSSVSPAATTACVP

YAPSRPPGLPGTTTSSSSSSSSSSSSSSNTGLRGAEPNPGIPGADHYQTPALDISSHQ

GRLGPSAHSSRKPFLAAPAATPHLSLPPGPPSPPPPPCPRLLRPPPPPAWLKGPACRA

AREDGEILEELFFGAEGRPRPPPPPLPHREGFLGPPAPRFSVGTQDSHTPPTPPATSS

SSSNHGSHSSSPTGPVSFPPPPYLARSLDPLPRPPSPTLSPQDPPLAPLTLALPPAPP

SSCHQNTSGSFRRPESPRPRVSFPKTPEVGPGPSPGPLNKAPQPVPPRVGELPARGPR

LFDFPPTPLEDQFEEPAEFKILPDGLANIMKMLDESIRKEEEQQQQEAGVGPPPPLKE

PFPSLQPSFPSDTAPATTAAPAATATTTTATQEEEKKPPPVLPPPPPLAKFPPPQPQP

PPPPPLPPAASPASLLKSLASVLEGQKYCYRGTGAAVATRPGPLPTTQYSPGPPSGAT

APPPASAAPSARGSPQPSASSSSQFSTSGGPWARERRAGEEPARGPTAPAPPPPPPPP

LPLPPARSESEVLEEISRACETLVERVGRSAADLAGPVDTAGPVDSGTERLLPPAQAR

EEGGGAVAAAGPGSGKRRQKEHQKEHQKEHRRHRRACKDSTGRRPREGRVKTKAKAPK

EKSRRVLGNLDLQSEEIQGREKARPDLGGASKAKPPAAPAPPPAPAPAAQPTPPSAPT

PGKKAREEAPGPPGVSRADMLKLRSLSEGPPKELKIRLIKVESGDKETFIASEVEERR

LRMADLTISHCAADVVRASKNAKVKGKFRESYLSPAQSVKPKINTEEKLPREKLNPPT

PSIYLESKRDAFSPVLLQFCTDPRNPITVIRGLAGSLRLNLGLFSTKTLVEASGEHTV

EVRTQVQQPSDENWDLTGTRQIWPCESSRSHTTIAKYAQYQASSFQESLQEEKESEDE

ESEEPDSTTGTPPSSAPDPKNHHIIKFGTNIDLSDAKRWKPQLQELLKLPAFMRVTST

GNMLSHVGHTILGMNTVQLYMKVPGSRTPGHQENNNFCSVNINIGPGDCEWFAVHEHY

WETISAFCDRHGVDYLTGSWWPILDDLYASNIPVYRFVQRPGDLVWINAGTVHWVQAT

GWCNNIAWNVGPLTAYQYQLALERYEWNEVKNVKSIVPMIHVSWNVARTVKISDPDLF

RMIKFCLLQSMKHCQVQRESLVRAGKKIAYQGRVKDEPAYYCNECDVEVFNILFVTSE

NGSRNTYLVHCEACARRRSAGLQGVVVLEQYRTEELAQAYDAFTLAPASTSR"

/note="identifier=.1"

/note="Derived by automated computational analysis using

gene prediction method:genscan"

variation	147..147

/replace="C/G"

/db_xref="EVA:rs5502625334"

variation	148..147

/replace="-/G"

/db_xref="EVA:rs5502615948"

variation	148..148

/replace="G/-/GG/GGG/GGGG"

/db_xref="EVA:rs5502587785"

variation	160..161

/replace="GA/-"

/db_xref="EVA:rs5502633281"

variation	161..161

/replace="A/-"

/db_xref="EVA:rs5502630965"

variation	317..317

/replace="T/C"

/db_xref="EVA:rs3329624656"

variation	590..590

/replace="G/A"

/db_xref="EVA:rs3329624753"

variation	717..717

/replace="A/G"

/db_xref="EVA:rs3329107878"

variation	764..764

/replace="C/T"

/db_xref="EVA:rs3329552780"

variation	1002..1001

/replace="-/T/TT"

/db_xref="EVA:rs3329439857"

variation	1002..1002

/replace="T/-/TT/TTTTTTT/TTTTTTTT"

/db_xref="EVA:rs3329374983"

variation	1024..1024

/replace="G/T"

/db_xref="EVA:rs3329439897"

variation	1026..1026

/replace="A/T"

/db_xref="EVA:rs3329553043"

variation	1041..1041

/replace="G/A"

/db_xref="EVA:rs3329107810"

variation	1441..1440

/replace="-/AA"

/db_xref="EVA:rs5502624191"

variation	1490..1491

/replace="TG/-"

/db_xref="EVA:rs5502576837"

variation	1493..1492

/replace="-/AGG"

/db_xref="EVA:rs5502623942"

variation	1496..1496

/replace="C/-"

/db_xref="EVA:rs5502631029"

variation	1587..1588

/replace="TC/-"

/db_xref="EVA:rs5502624348"

variation	1591..1590

/replace="-/CG"

/db_xref="EVA:rs5502633080"

variation	1673..1673

/replace="C/T"

/db_xref="EVA:rs3329619261"

variation	1724..1724

/replace="C/T"

/db_xref="EVA:rs3329713739"

variation	1885..1886

/replace="GA/-"

/db_xref="EVA:rs5502643917"

variation	2065..2064

/replace="-/TT"

/db_xref="EVA:rs5502624861"

variation	2066..2067

/replace="GA/-"

/db_xref="EVA:rs5502631700"

variation	2086..2085

/replace="-/GCA"

/db_xref="EVA:rs5502625305"

variation	2089..2088

/replace="-/AA"

/db_xref="EVA:rs5502629439"

variation	2092..2091

/replace="-/TGAT"

/db_xref="EVA:rs5502624183"

variation	2096..2095

/replace="-/GT"

/db_xref="EVA:rs5502633176"

variation	2098..2097

/replace="-/ATCTGAGG"

/db_xref="EVA:rs5502643916"

variation	2103..2103

/replace="G/A"

/db_xref="EVA:rs3329439840"

variation	2158..2157

/replace="-/T/TT"

/db_xref="EVA:rs3329638108"

variation	2158..2158

/replace="T/-/TT/TTT"

/db_xref="EVA:rs850854743"

variation	2173..2173

/replace="T/C"

/db_xref="EVA:rs3329619513"

variation	2174..2173

/replace="-/C/TC"

/db_xref="EVA:rs5502588089"

variation	2211..2211

/replace="A/G"

/db_xref="EVA:rs850574130"

variation	2216..2215

/replace="-/A"

/db_xref="EVA:rs5502624827"

variation	2376..2376

/replace="C/T"

/db_xref="EVA:rs3329624606"

variation	2377..2376

/replace="-/G"

/db_xref="EVA:rs852899619"

variation	2435..2435

/replace="C/T"

/db_xref="EVA:rs852440243"

variation	2447..2447

/replace="G/A"

/db_xref="EVA:rs850818639"

variation	2462..2462

/replace="C/T"

/db_xref="EVA:rs852701327"

variation	2463..2463

/replace="G/A"

/db_xref="EVA:rs852416666"

variation	2528..2527

/replace="-/TTGCTGGAGATGACAAACACCCATGCTTGT"

/db_xref="EVA:rs3329624803"

variation	2746..2745

/replace="-/AGT"

/db_xref="EVA:rs5502642787"

variation	2749..2751

/replace="AGG/-"

/db_xref="EVA:rs5502629328"

variation	2782..2782

/replace="A/G"

/db_xref="EVA:rs3329107923"

variation	2795..2795

/replace="C/T"

/db_xref="EVA:rs851620436"

variation	2796..2796

/replace="G/A"

/db_xref="EVA:rs852661628"

variation	2798..2798

/replace="C/T"

/db_xref="EVA:rs851872131"

variation	2799..2799

/replace="G/A"

/db_xref="EVA:rs851255145"

variation	2804..2804

/replace="A/G"

/db_xref="EVA:rs851131395"

variation	2871..2870

/replace="-

/CAGGCGGCTCATAGGGCACCACCACACTATGTCGAAAAGTGTTTCTGTCATCCAAAT

ACTCCACACGCAAATTTCCTTCCACTCGGATAAGATGCTGAG"

/db_xref="EVA:rs5502642893"

variation	2875..2874

/replace="-

/CGGCTCATAGGGCACCACCACACTATGTCGAAAAGTGTTTCTGTCATCCAAATACTC

CACACGCAAATTTCCTTCCACTCGGA"

/db_xref="EVA:rs5502619449"

variation	2932..2932

/replace="C/A"

/db_xref="EVA:rs3329639853"

variation	3029..3029

/replace="T/C"

/db_xref="EVA:rs3329665997"

variation	3072..3073

/replace="CT/-"

/db_xref="EVA:rs5502643924"

variation	3080..3080

/replace="C/-"

/db_xref="EVA:rs5502629457"

variation	3090..3090

/replace="C/T"

/db_xref="EVA:rs853180251"

variation	3142..3142

/replace="G/A"

/db_xref="EVA:rs3329107894"

variation	3153..3155

/replace="TGG/-"

/db_xref="EVA:rs5502642963"

variation	3158..3157

/replace="-/ACA"

/db_xref="EVA:rs5502631694"

variation	3229..3228

/replace="-/A"

/db_xref="EVA:rs5502629397"

variation	3238..3237

/replace="-/A"

/db_xref="EVA:rs5502624108"

variation	3242..3244

/replace="CTG/-"

/db_xref="EVA:rs5502624100"

variation	3248..3247

/replace="-/CA"

/db_xref="EVA:rs5502633279"

variation	3260..3259

/replace="-/GT"

/db_xref="EVA:rs5502611723"

variation	3261..3262

/replace="CA/-"

/db_xref="EVA:rs5502619493"

variation	3266..3298

/replace="GTAGCCCCCGATGGCTTACCGTCACTACTGTCA/-"

/db_xref="EVA:rs5502624136"

variation	3278..3278

/replace="G/-"

/db_xref="EVA:rs5502616022"

variation	3293..3295

/replace="CTG/-"

/db_xref="EVA:rs5502629460"

variation	3304..3304

/replace="G/T"

/db_xref="EVA:rs3329553012"

variation	3323..3323

/replace="C/T"

/db_xref="EVA:rs851387419"

variation	3373..3373

/replace="G/C"

/db_xref="EVA:rs3329454336"

variation	3403..3406

/replace="GCTG/-"

/db_xref="EVA:rs5502624395"

variation	3406..3406

/replace="G/A"

/db_xref="EVA:rs5502643025"

variation	3407..3406

/replace="-/ATCA"

/db_xref="EVA:rs5502633217"

variation	3443..3443

/replace="C/A"

/db_xref="EVA:rs850764404"

variation	3494..3494

/replace="C/T"

/db_xref="EVA:rs3329439705"

variation	3514..3514

/replace="C/G"

/db_xref="EVA:rs3329553444"

variation	3705..3705

/replace="C/T"

/db_xref="EVA:rs3329600235"

variation	4001..4001

/replace="G/A"

/db_xref="EVA:rs3329454402"

variation	4147..4147

/replace="C/T"

/db_xref="EVA:rs3329639995"

variation	4195..4195

/replace="C/G"

/db_xref="EVA:rs3329553301"

variation	4210..4210

/replace="A/-"

/db_xref="EVA:rs852650056"

variation	4225..4225

/replace="G/A"

/db_xref="EVA:rs3329454345"

variation	4388..4387

/replace="-/AC"

/db_xref="EVA:rs3329619396"

variation	4388..4388

/replace="G/A"

/db_xref="EVA:rs852388548"

variation	4414..4414

/replace="T/G"

/db_xref="EVA:rs5502631003"

variation	4415..4414

/replace="-/TGGGGGGGGGGGGGGGGGGGGGGGGGGGGGGGGGG"

/db_xref="EVA:rs5502587850"

variation	4422..4421

/replace="-/G"

/db_xref="EVA:rs5502588073"

variation	4426..4426

/replace="T/G"

/db_xref="EVA:rs3329624799"

variation	4484..4484

/replace="G/A"

/db_xref="EVA:rs3329439929"

variation	4525..4525

/replace="C/T"

/db_xref="EVA:rs3329439873"

variation	4627..4626

/replace="-/C"

/db_xref="EVA:rs5502624350"

variation	4673..4673

/replace="T/C"

/db_xref="EVA:rs3329375045"

variation	4819..4819

/replace="T/C"

/db_xref="EVA:rs3329439748"

variation	4934..4934

/replace="C/T"

/db_xref="EVA:rs3329553333"

variation	4965..4965

/replace="C/T"

/db_xref="EVA:rs3329107888"

variation	5011..5011

/replace="C/T"

/db_xref="EVA:rs3329624827"

variation	5029..5029

/replace="T/C"

/db_xref="EVA:rs3329619474"

variation	5088..5092

/replace="GGGGT/-/GGGGTT"

/db_xref="EVA:rs3329600362"

variation	5093..5093

/replace="T/C"

/db_xref="EVA:rs3329107929"

variation	5097..5097

/replace="T/C"

/db_xref="EVA:rs3329107950"

variation	5221..5221

/replace="G/A"

/db_xref="EVA:rs3329107781"

variation	5232..5231

/replace="-/T"

/db_xref="EVA:rs5502643755"

variation	5238..5238

/replace="T/A"

/db_xref="EVA:rs3329665739"

variation	5488..5488

/replace="C/T"

/db_xref="EVA:rs24181023"

variation	5552..5551

/replace="-/A"

/db_xref="EVA:rs3329439680"

variation	5889..5889

/replace="T/C"

/db_xref="EVA:rs5515137825"

variation	6027..6027

/replace="T/A"

/db_xref="EVA:rs3329624724"

variation	6032..6035

/replace="TTTT/-"

/db_xref="EVA:rs3329553448"

variation	6036..6035

/replace="-/AAAA"

/db_xref="EVA:rs3329374818"

variation	6219..6218

/replace="-/GGATCCCTAGGTGGCGCAGCGGTTTGGCGCCTGCCTTCGGCCT"

/db_xref="EVA:rs5502624860"

variation	6291..6290

/replace="-/TAAT"

/db_xref="EVA:rs5502643051"

variation	6471..6471

/replace="G/A"

/db_xref="EVA:rs3329454282"

variation	6601..6601

/replace="T/G"

/db_xref="EVA:rs3329619518"

variation	6602..6602

/replace="A/T"

/db_xref="EVA:rs3329639736"

variation	6817..6817

/replace="T/G"

/db_xref="EVA:rs3329639977"

variation	6913..6913

/replace="A/T"

/db_xref="EVA:rs3329375077"

variation	7061..7063

/replace="AAT/-"

/db_xref="EVA:rs5502624391"

variation	7063..7063

/replace="T/A"

/db_xref="EVA:rs3329439754"

variation	7099..7098

/replace="-/TTTTTTTT"

/db_xref="EVA:rs5502633297"

variation	7099..7099

/replace="T/-"

/db_xref="EVA:rs5502611733"

variation	7251..7251

/replace="C/A"

/db_xref="EVA:rs3329639960"

variation	7259..7259

/replace="C/G"

/db_xref="EVA:rs3329677715"

variation	7431..7430

/replace="-/A"

/db_xref="EVA:rs5502631045"

variation	7440..7439

/replace="-/A"

/db_xref="EVA:rs5502625211"

variation	7753..7753

/replace="G/A"

/db_xref="EVA:rs3329619326"

variation	7755..7755

/replace="T/C"

/db_xref="EVA:rs3329439734"

variation	7776..7776

/replace="G/A"

/db_xref="EVA:rs3329624668"

variation	7798..7798

/replace="A/G"

/db_xref="EVA:rs3329600388"

variation	7810..7810

/replace="G/A"

/db_xref="EVA:rs3329619501"

variation	7818..7817

/replace="-/GG"

/db_xref="EVA:rs3329759609"

variation	7915..7914

/replace="-/A"

/db_xref="EVA:rs5502624367"

variation	7915..7915

/replace="A/-"

/db_xref="EVA:rs5502619469"

variation	7930..7929

/replace="-/A"

/db_xref="EVA:rs5502611741"

variation	7930..7930

/replace="A/-/AA"

/db_xref="EVA:rs3329553292"

variation	8090..8089

/replace="-/G"

/db_xref="EVA:rs5502631737"

variation	8092..8094

/replace="AAA/-"

/db_xref="EVA:rs5502619389"

variation	8095..8094

/replace="-/TTTATTT"

/db_xref="EVA:rs5502643876"

variation	8100..8099

/replace="-/A/AA"

/db_xref="EVA:rs5502624754"

variation	8100..8100

/replace="A/-"

/db_xref="EVA:rs5502611679"

variation	8120..8119

/replace="-

/GGA/GGATCCC/GGATCCCTGGGTGGCGCAGCGGTTTAGCGCCTGCCTT/GGATCCC

TGGGTGGCGCAGCGGTTTAGCGCCTGCCTTTGGCCCAGGGCACGATCC"

/db_xref="EVA:rs5502611635"

variation	8120..8120

/replace="T/G"

/db_xref="EVA:rs5502629448"

variation	8121..8120

/replace="-/CCCTGGGTGGCGCAGCG"

/db_xref="EVA:rs5502619499"

variation	8122..8121

/replace="-

/GGTGGCGCAGCGGT/GGTGGCGCAGCGGTTTAGCGCCTGCC/GGTGGCGCAGCGGTT

TAGCGCCTGCCT"

/db_xref="EVA:rs5502588055"

variation	8122..8123

/replace="TT/-"

/db_xref="EVA:rs5502611540"

variation	8124..8123

/replace="-/CCCTGGGTGGCGC/TAGCGCCTGCCTTTGGCCCAGGGCACG"

/db_xref="EVA:rs5502624402"

variation	8175..8179

/replace="ATAAC/-"

/db_xref="EVA:rs3329439820"

variation	8199..8198

/replace="-/TA"

/db_xref="EVA:rs5502629382"

variation	8199..8200

/replace="TA/-/TATA"

/db_xref="EVA:rs3329439699"

variation	8211..8212

/replace="TT/-"

/db_xref="EVA:rs5502633132"

variation	8233..8233

/replace="A/T"

/db_xref="EVA:rs3329375092"

variation	8345..8344

/replace="-/GAAGCTTAACC"

/db_xref="EVA:rs3329107886"

variation	8391..8390

/replace="-/A"

/db_xref="EVA:rs8614223"

variation	8671..8674

/replace="TTTG/-"

/db_xref="EVA:rs5502624712"

variation	8725..8725

/replace="G/A"

/db_xref="EVA:rs3329439912"

variation	8752..8752

/replace="G/T"

/db_xref="EVA:rs3329624831"

variation	8761..8761

/replace="C/A"

/db_xref="EVA:rs3329454359"

variation	8808..8808

/replace="T/G"

/db_xref="EVA:rs3329553341"

variation	8856..8856

/replace="A/G"

/db_xref="EVA:rs3329553403"

variation	8883..8882

/replace="-/C"

/db_xref="EVA:rs5502642890"

variation	8887..8887

/replace="T/-"

/db_xref="EVA:rs5502611755"

variation	8892..8892

/replace="A/-"

/db_xref="EVA:rs5502624345"

variation	9013..9013

/replace="A/C"

/db_xref="EVA:rs3329665895"

variation	9117..9117

/replace="C/T"

/db_xref="EVA:rs3329677893"

variation	9323..9323

/replace="A/-"

/db_xref="EVA:rs3329553346"

variation	9385..9385

/replace="C/T"

/db_xref="EVA:rs3329665954"

variation	9438..9438

/replace="A/G"

/db_xref="EVA:rs3329454262"

variation	9449..9449

/replace="G/A"

/db_xref="EVA:rs3329600387"

variation	9513..9512

/replace="-/AAATA"

/db_xref="EVA:rs5502576952"

variation	9513..9517

/replace="AAATA/-"

/db_xref="EVA:rs3329619496"

variation	9551..9551

/replace="G/T"

/db_xref="EVA:rs3329107909"

variation	9642..9642

/replace="A/T"

/db_xref="EVA:rs3329454332"

variation	9671..9671

/replace="C/T"

/db_xref="EVA:rs3329375012"

variation	9930..9929

/replace="-/GG"

/db_xref="EVA:rs3329710170"

variation	9931..9930

/replace="-/TAATGTATAAT"

/db_xref="EVA:rs3329638427"

variation	9931..9931

/replace="C/G"

/db_xref="EVA:rs3329374954"

variation	10017..10017

/replace="G/A"

/db_xref="EVA:rs3329624733"

variation	10028..10028

/replace="A/G"

/db_xref="EVA:rs3329439859"

variation	10065..10065

/replace="C/T"

/db_xref="EVA:rs5515138157"

variation	10092..10096

/replace="CTTTT/-"

/db_xref="EVA:rs3329624820"

variation	10102..10102

/replace="C/T"

/db_xref="EVA:rs3329375063"

variation	10216..10216

/replace="C/G"

/db_xref="EVA:rs3329107855"

variation	10270..10270

/replace="G/T"

/db_xref="EVA:rs3329624847"

variation	10285..10285

/replace="T/C"

/db_xref="EVA:rs3329709838"

variation	10357..10360

/replace="ACAC/-"

/db_xref="EVA:rs5502624432"

variation	10378..10378

/replace="G/A"

/db_xref="EVA:rs3329374995"

variation	10517..10517

/replace="T/C"

/db_xref="EVA:rs3329374987"

variation	10759..10758

/replace="-/CT"

/db_xref="EVA:rs5502611685"

variation	10780..10780

/replace="A/-"

/db_xref="EVA:rs3329375080"

variation	10810..10810

/replace="C/T"

/db_xref="EVA:rs3329638318"

variation	10942..10942

/replace="G/T"

/db_xref="EVA:rs3329374891"

variation	11270..11269

/replace="-/AAAAC"

/db_xref="EVA:rs3329639834"

variation	11288..11287

/replace="-/AC"

/db_xref="EVA:rs3329638397"

variation	11343..11343

/replace="G/A"

/db_xref="EVA:rs3329677895"

variation	11433..11432

/replace="-/A"

/db_xref="EVA:rs5502611696"

variation	11468..11467

/replace="-/GTG"

/db_xref="EVA:rs5502576948"

variation	11679..11678

/replace="-/C"

/db_xref="EVA:rs9170627"

variation	11764..11764

/replace="C/T"

/db_xref="EVA:rs3329619531"

variation	11790..11790

/replace="G/A"

/db_xref="EVA:rs3329624693"

variation	11874..11874

/replace="G/A"

/db_xref="EVA:rs3329600313"

variation	11940..11939

/replace="-

/CTCGG/CTCGGCTCGG/CTCGGCTCGGCTCGG/CTCGGCTCGGCTCGGCTCGG"

/db_xref="EVA:rs3329374890"

variation	11940..11944

/replace="CTCGG/-/CTCGGCTCGGCTCGG"

/db_xref="EVA:rs3329619446"

variation	12014..12014

/replace="T/C"

/db_xref="EVA:rs3356392105"

variation	12316..12315

/replace="-/G"

/db_xref="EVA:rs5502588025"

variation	12361..12361

/replace="A/G"

/db_xref="EVA:rs3329624558"

variation	12363..12363

/replace="T/A"

/db_xref="EVA:rs3329375002"

variation	12366..12366

/replace="G/A"

/db_xref="EVA:rs3329600329"

variation	12370..12370

/replace="G/A"

/db_xref="EVA:rs3329639940"

variation	12373..12372

/replace="-

/AAAAG/AAAAGAAAAG/AAAAGAAAAGAAAAG/AAAAGAAAAGAAAAGAAAAG/AAA

AGAAAAGAAAAGAAAAGAAAAG"

/db_xref="EVA:rs3329107725"

variation	12373..12377

/replace="AAAAG/-"

/db_xref="EVA:rs3329639939"

variation	12375..12375

/replace="A/T"

/db_xref="EVA:rs3329619505"

variation	12377..12377

/replace="G/A"

/db_xref="EVA:rs3329375093"

variation	12385..12387

/replace="AAG/-"

/db_xref="EVA:rs5502624808"

variation	12491..12490

/replace="-/A"

/db_xref="EVA:rs5502615991"

variation	12581..12581

/replace="G/A"

/db_xref="EVA:rs3329107690"

variation	12755..12754

/replace="-/C"

/db_xref="EVA:rs5502624411"

variation	12821..12821

/replace="A/G"

/db_xref="EVA:rs3329638296"

variation	12856..12855

/replace="-/A"

/db_xref="EVA:rs5502624396"

variation	13041..13041

/replace="G/A"

/db_xref="EVA:rs3329553402"

variation	13190..13190

/replace="C/T"

/db_xref="EVA:rs3329677821"

variation	13220..13220

/replace="G/A"

/db_xref="EVA:rs3329713664"

variation	13228..13228

/replace="C/T"

/db_xref="EVA:rs3329759769"

variation	13279..13279

/replace="G/C"

/db_xref="EVA:rs3329600341"

variation	13341..13345

/replace="GAGTC/-"

/db_xref="EVA:rs5502625281"

variation	13345..13344

/replace="-/C"

/db_xref="EVA:rs5502576856"

variation	13347..13346

/replace="-/TT"

/db_xref="EVA:rs5502642973"

misc_feature	complement(814..891)

/note="match: A0A0C5DEW5.1 : 356..381(1)"

misc_feature	complement(814..891)

/note="match: Q29537.2 : 356..381(1)"

misc_feature	complement(814..4555)

/note="match: E7FIY6.1 : 1..381(1)"

misc_feature	complement(814..4555)

/note="match: Q29537.2 : 1..381(1)"

misc_feature	complement(1516..1620)

/note="match: Q29537.2 : 320..354(1)"

misc_feature	complement(2001..2072)

/note="match: Q29537.2 : 296..319(1)"

misc_feature	complement(2362..2496)

/note="match: Q29537.2 : 250..294(1)"

misc_feature	complement(2761..2868)

/note="match: Q29537.2 : 213..248(1)"

misc_feature	complement(3091..3201)

/note="match: Q29537.2 : 176..212(1)"

misc_feature	complement(3286..3471)

/note="match: Q29537.2 : 113..174(1)"

misc_feature	complement(3974..4165)

/note="match: Q29537.2 : 49..112(1)"

misc_feature	complement(4484..4555)

/note="match: A0A0C5DEW5.1 : 1..24(1)"

misc_feature	complement(4484..4555)

/note="match: Q29537.2 : 1..24(1)"

repeat_region	complement(142..160)

/note="dust repeat: matches 2807732..2807750(1) of

consensus"

repeat_region	complement(357..460)

/note="MIRc repeat: matches 102..213(1) of consensus"

repeat_region	complement(1002..1023)

/note="dust repeat: matches 2808592..2808613(1) of

consensus"

repeat_region	complement(1002..1023)

/note="trf repeat: matches 1..22(1) of consensus"

repeat_region	complement(1110..1230)

/note="MIRc repeat: matches 18..149(1) of consensus"

repeat_region	complement(1631..1637)

/note="dust repeat: matches 2809221..2809227(1) of

consensus"

repeat_region	complement(1926..1932)

/note="dust repeat: matches 2809516..2809522(1) of

consensus"

repeat_region	complement(2158..2173)

/note="dust repeat: matches 2809748..2809763(1) of

consensus"

repeat_region	complement(2162..2329)

/note="SINEC1_AMe repeat: matches 1..201(1) of consensus"

repeat_region	complement(2216..2222)

/note="dust repeat: matches 2809806..2809812(1) of

consensus"

repeat_region	2906..3011

/note="MIRc repeat: matches 93..202(1) of consensus"

repeat_region	complement(3019..3074)

/note="dust repeat: matches 2810609..2810664(1) of

consensus"

repeat_region	complement(3565..3697)

/note="SINEC_a2 repeat: matches 1..158(1) of consensus"

repeat_region	complement(3573..3611)

/note="trf repeat: matches 1..39(1) of consensus"

repeat_region	complement(4382..4458)

/note="dust repeat: matches 2811972..2812048(1) of

consensus"

repeat_region	5014..5201

/note="L2c repeat: matches 2960..3168(1) of consensus"

repeat_region	complement(5232..5339)

/note="SINEC_c1 repeat: matches 101..204(1) of consensus"

repeat_region	complement(5232..5238)

/note="dust repeat: matches 2812822..2812828(1) of

consensus"

repeat_region	complement(5267..5316)

/note="dust repeat: matches 2812857..2812906(1) of

consensus"

repeat_region	complement(5552..5558)

/note="dust repeat: matches 2813142..2813148(1) of

consensus"

repeat_region	complement(5782..5860)

/note="MIR3_MarsA repeat: matches 57..140(1) of consensus"

repeat_region	5873..6042

/note="SINEC_a1 repeat: matches 1..177(1) of consensus"

repeat_region	complement(6036..6042)

/note="dust repeat: matches 2813626..2813632(1) of

consensus"

repeat_region	6238..6324

/note="MIR repeat: matches 21..120(1) of consensus"

repeat_region	6319..6359

/note="MIR repeat: matches 219..258(1) of consensus"

repeat_region	complement(6868..7033)

/note="MIR repeat: matches 8..196(1) of consensus"

repeat_region	complement(7061..7079)

/note="dust repeat: matches 2814651..2814669(1) of

consensus"

repeat_region	complement(7099..7105)

/note="dust repeat: matches 2814689..2814695(1) of

consensus"

repeat_region	complement(7409..7415)

/note="dust repeat: matches 2814999..2815005(1) of

consensus"

repeat_region	complement(7431..7444)

/note="dust repeat: matches 2815021..2815034(1) of

consensus"

repeat_region	complement(7492..7558)

/note="Tigger2a_Car repeat: matches 278..341(1) of

consensus"

repeat_region	7559..7726

/note="SINEC1_AMe repeat: matches 1..196(1) of consensus"

repeat_region	complement(7719..7726)

/note="dust repeat: matches 2815309..2815316(1) of

consensus"

repeat_region	7727..7924

/note="SINEC1_AMe repeat: matches 1..201(1) of consensus"

repeat_region	complement(7849..7885)

/note="dust repeat: matches 2815439..2815475(1) of

consensus"

repeat_region	complement(7849..7885)

/note="trf repeat: matches 1..37(1) of consensus"

repeat_region	complement(7896..7941)

/note="dust repeat: matches 2815486..2815531(1) of

consensus"

repeat_region	complement(7925..7945)

/note="Tigger2a_Car repeat: matches 276..277(1) of

consensus"

repeat_region	7946..8112

/note="SINEC1_AMe repeat: matches 1..201(1) of consensus"

repeat_region	complement(8038..8077)

/note="dust repeat: matches 2815628..2815667(1) of

consensus"

repeat_region	complement(8100..8113)

/note="dust repeat: matches 2815690..2815703(1) of

consensus"

repeat_region	complement(8113..8222)

/note="Tigger2a_Car repeat: matches 167..275(1) of

consensus"

repeat_region	complement(8195..8217)

/note="dust repeat: matches 2815785..2815807(1) of

consensus"

repeat_region	complement(8223..8365)

/note="SINEC1D_CF repeat: matches 2..193(1) of consensus"

repeat_region	complement(8226..8239)

/note="dust repeat: matches 2815816..2815829(1) of

consensus"

repeat_region	complement(8366..8523)

/note="Tigger2a_Car repeat: matches 2..166(1) of

consensus"

repeat_region	complement(8423..8429)

/note="dust repeat: matches 2816013..2816019(1) of

consensus"

repeat_region	complement(8911..9356)

/note="L1M5 repeat: matches 4494..4648(1) of consensus"

repeat_region	9357..9536

/note="SINEC1_AMe repeat: matches 2..197(1) of consensus"

repeat_region	complement(9505..9537)

/note="dust repeat: matches 2817095..2817127(1) of

consensus"

repeat_region	complement(9513..9535)

/note="trf repeat: matches 1..23(1) of consensus"

repeat_region	complement(9537..9809)

/note="L1M5 repeat: matches 4220..4493(1) of consensus"

repeat_region	complement(9810..9882)

/note="tRNA-Lys-AAG repeat: matches 2..74(1) of consensus"

repeat_region	complement(9883..10540)

/note="L1M5 repeat: matches 3547..4219(1) of consensus"

repeat_region	complement(10092..10113)

/note="dust repeat: matches 2817682..2817703(1) of

consensus"

repeat_region	complement(10333..10374)

/note="dust repeat: matches 2817923..2817964(1) of

consensus"

repeat_region	complement(10333..10374)

/note="trf repeat: matches 1..42(1) of consensus"

repeat_region	complement(10333..10374)

/note="trf repeat: matches 1..42(1) of consensus"

repeat_region	10533..10639

/note="L1ME2 repeat: matches 2243..2355(1) of consensus"

repeat_region	10642..11727

/note="L1ME2 repeat: matches 5054..6223(1) of consensus"

repeat_region	complement(11262..11330)

/note="dust repeat: matches 2818852..2818920(1) of

consensus"

repeat_region	complement(11264..11290)

/note="trf repeat: matches 1..27(1) of consensus"

repeat_region	complement(11433..11440)

/note="dust repeat: matches 2819023..2819030(1) of

consensus"

repeat_region	complement(11937..12007)

/note="dust repeat: matches 2819527..2819597(1) of

consensus"

repeat_region	complement(11940..11970)

/note="trf repeat: matches 1..31(1) of consensus"

repeat_region	complement(12319..12401)

/note="trf repeat: matches 1..83(1) of consensus"

repeat_region	complement(12320..12401)

/note="dust repeat: matches 2819910..2819991(1) of

consensus"

repeat_region	complement(12320..12400)

/note="trf repeat: matches 1..81(1) of consensus"

repeat_region	complement(12322..12400)

/note="trf repeat: matches 1..79(1) of consensus"

repeat_region	complement(12491..12498)

/note="dust repeat: matches 2820081..2820088(1) of

consensus"

repeat_region	complement(12755..12761)

/note="dust repeat: matches 2820345..2820351(1) of

consensus"

misc_feature	1..13362

/note="contig 5 32665394..32678755(1)"

BASE COUNT	3598 a	3209 c	3343 g	3212 t	0 n

ORIGIN

1 TGGTAGTAAA GTTTTATTGT AAAATAAGAG ATTGATATAA AAACGGGATA TAAAAAGGGA

61 AGAGGAGGAG GGGTGGGGGT GATCAATGCA TGAATGCAGA TGTGCCTCAC AGAGTGCAAA

121 AGAAATTGAT CCTGAGCAAT TGGGGGCGGG GGGGGGGGGG AAGCAGTAAA AGCCCTTGGT

181 AGACAAAGAT CCTACACCAG GGATGGGATT CTGCAGGGTG TGAGATGGGA GTGAGATTTC

241 ATCTTAGGTG CAGGGGTCTC CCAAAAAGGC TGGACAGGAT TTGGCCAGGG CAGGGAATGA

301 GCACCCACAC AGGCACTAAG TCACAGACAT GGGATAAAAG GTGGTTTTGG GGTCAGACAG

361 ACATTATTTC ATTCATCCTC ACAAGCTCAG TGAGGTAGGT ATGGTTAATT AGCAATTCAT

421 AAAAATGGCC TGTGAAATTA GGAAAAATTC AGCCACTGGC TACAATGATA GAGAACTGGG

481 CTCATGACTC AGTGTCTTAG GGGCTATAAA ATGCTCCCAA CATTCAGGAA CATTTTCCTA

541 CCTCTCCCAT GGTTCTACAC AGTAAAAAAC TTAAAAACCA GGCTGGAACG TCCTTTATCT

601 CCCGACCTAC CAACTGTGCA GGCCAACTTC TTTAGTAGAG ACTTACGGGA AAGGCAAATG

661 GAAGAATCTA GGGTGTTTCT GAGGCACATC TGTACCATGC AAAGTAACAC ACCGGAATAG

721 GTGTGCTCAA GCTCCCAATT CCAAAAATGC CAGGGAAGGA GGACGAGAGT GAGGGTGTGC

781 GTGTTGGGGG AGGACAGGAA GCAGGGGAAG TCAGTCTGAG TCGGGCCCTT CTCTCTTGAA

841 CATCAGTTTT TTATGGCGAG AGGTAGATTG CCCCTTCTTT GCCTTCAGGT GGCTGTTGGG

901 GACACAAGGT GGAGGGGAAG AGACGATATT AGCAAGTGGG AGCCTCGGGG CTCCTTGTCC

961 TAGATCTAGG CTTGGAAGGG TCCAAGGCTG GAGTAAGGGA ATTTTTTTTT TTTTTTTTTT

1021 TTTGGAATGA GGTAATTTAA GTTAAAATGC TGTAGACACA TTTACTGAGC ACCCAGGGAA

1081 CTTCTGTGTG TTCGCTTCCT CTCCCCTCAC CCCATCTGAT GAACAAGGAA ACCGAGCCCC

1141 AGAAGGGGAG ATGCAAGGCC ACACGGATCT GCTCATCCTT GGCAGGGCTG GGGGTCTGGC

1201 CCAATCCTGC TGTGTCCAGT ATTTTCCCAC ACGGTTATCA GGTCCCTATT GGAGACCCCA

1261 CTTGGCACTA GGGGTTGCTG GAGATGACAA ACACCCATGC TTGTCCCTTA AGGGGTTTGG

1321 GGACCTCCCG AGGCAGAAAA GGGAGTACAG GATCCAACTG GGGGTATGGT AGAAGCAGGT

1381 TTGCCCAGGG TGGGACCAAC CATGAGGGGA TCAGTTCCCT TCCACCTTGA AGGTAGGAGT

1441 AGAATGGAAT CCTGTGGCTT TCCAACATAT GAAGGCAAGG GGGTGGGGCT GCAAGCCGGC

1501 CCAGGTCACT CACCTGGAGT GAGCCCTGCT TCCCCCTGGC TCCTTTCCAC TCTGGGCATC

1561 CTTCAGCTCC AAGGCTTCAT TCAGATTCCT GAACATCTCA TAGCGTTCAC GCCCACGGAT

1621 CTGTGGCAGT GGGGGGGAGG AGGAGAAAGA ATAAGAGGGG GAAGCCACAG TACCATTTAA

1681 CTCAGACTAA GGTGTAAGTG CCAATCTGCA GCAAGGCACA GCCCGCAGCC ACTGTCAAGG

1741 CACCGCTGCC CCTTGATGGC AAGTGTGCTA ATTGCAGGCG GAGCAGTCAA AAGAAAACTG

1801 TGCTCTTGGC CCTCAGCTTG AAACAAATCT TCAATCCAAG AATATCAACT TCAAGAATCA

1861 GGGACAGGAC CAGAGAAAAG GATGGAGAGA GGAACAGGCC TAGCCTCACA TGTGCTCCAT

1921 TCACATTTTT TTCTTCCCTC ATCTGATCCC GCTAAGCGGG GGCAAGAGCC TGCCTTATCT

1981 GTTCCTCCCA GCCTTGGTAC CTGAAGGGTG AAATATTCTC CATCTAGTGG CTTCTTCTTT

2041 TGCGGGGGAG AGGAGCTGGT GCTGGGAGGC AGTGCTGGGA AAGAGAGGAG GAAAAGACAA

2101 GAGAAGAGTA TGTTTTGAGC AGAATTGCAC CCTCTGTATC TGCCCCACAT CCTCCTCTTT

2161 TTTTTTTTTT TTTCAAGATT ATATTTATTT ATTCATGAGA CATACACACA AAGGCAAAAA

2221 AATAGGCAGA GGGAGAGGCA GGCTCCCTAC AGGGACCCTG ACACGGGACT CGATCCCAGG

2281 ACCCTGGGAT CATGCCTGAT GCTCAACCCC TGAGCCACCC AGGTGTCCCT CCTTCACCTC

2341 CTCTTGTCTT GCTTGCTTAC CTCGCTTGGT ACTCCCGGGG GGTGGCTCAG GACAAGGCTC

2401 CCCCTTCTTG TGGAAATTCT CCTCCTCAGT CCGGCGGTCT CTCCCGGGAC AGGCACAAAC

2461 GCGTACCTCA AAGCTGTTGC GTCCCAGCAC GTTTCCACTA CCCAGGTGAG AAGAGAGAAG

2521 CAGGGGGCCG TGAGGGTGGT GGGCCCCACT GACTCCTAGC CACCCTCATC CTACGTAGAG

2581 CCAAAGCCTA CAGACTATTG AGAAGCCACA GGTCAGGGAG GTCCCAAAGC CAGAGGAAAG

2641 AAAACCAGGG GAGGCGGATG GCAGAGGAGA TTCCACATTG GGACAGGAAT GGATGGGAAG

2701 GATGGAGGGG GGTACAGTAA GGAAGTGGCC AGGAGCAGGG TGGCAGGCAG GTCCCTACCT

2761 GGAGTCTTCC AGGGTGATGA TAGTGAGGAT GGGCCGCCGG TTCATGCCTC CCATGCAGGA

2821 ACTGTTACAC ATGTAGTTGT AGTGGATGGT GGTATAGTCA GAGCCAACCT GAGGTAGAAG

2881 GTAGGCCCAG GGTCGGGCCA CTCAGCCCTG GGCAGGCCTG TAGTCTATCA GCCTCAGTCT

2941 TTCCCAACCG CAGAATGGGA TGACCCACCT GCCTCTGCAC TCCTCACAGG GCTGCGGGGA

3001 GGTTGAAGGA GGAGAAAGCC CCCTCAACTC CCGCCTCACA AATCCCTCCA GCCCCCTCCT

3061 CCCAGAGACC CCTCAGATGC CAAACCAGAC CTCGGGTGGC TCATAAGGCA CCACCACACT

3121 GTGTCGAAAA GTGTTTCTGT CGTCCAGGTA CTTGGCCCGC AAATTTCCTT CCACTCGGAT

3181 GAGATGCTGA GGAGGGGCAA GACCTAAGAG CCATCGGGGA GGAATCAGGG CTTTGGGCCC

3241 TCTGGGGAGC AGCCTTGTCC CATCTGTAGC CCCCGATGGC TTACCGTCAC TACTGTCAGA

3301 GCAGCGTTCA TGGTGGGGGC AGCGCCGCAC AACCTCGGTC ACGAACTCCG ACTTCTTATA

3361 GATGGCCATA GCGCGGACGC AGGTATTGGG TGGGGGTGGG GAGCTGACCC ACAGCTGCAC

3421 GGGGCAGGTC TTCGCCAGCT GGCAAAACAA CTTGTTGAGG AGAGGGGAGT ACTGTAAGAA

3481 AAGGACAGAT GGACAGGTCA GGGCATGTGT GCCCGGGGTG GGTGGTGAGA AGTAGAGGGG

3541 TCGGGGTCTA TTCCAGAAGC TTACTTTATT TTAGACACAC AGAGAGAGGC AGAGACACAG

3601 GCAGAGGGAG AAGTGGTATG GGATTCGATC CCAGGACCTG GGGCTCACAC CCTGAACCAA

3661 AGGCAGACGC TCAACTGCTG AGCTGCCCAG GCATCTCCCA GAAGCTTACT TTAAAAGCCA

3721 AGGAATGAAC AAGTGAAGGA AAATGAAGAA GAGTCCCTTT GCCCCATGAC AGGAAAGGTG

3781 AAAGCCCAAG GTCAAGGGGG GAAATGTCAA CTTTGACACA GAGCTGAGGC CAAAGGTCAC

3841 AGAATACATA GGTAATGCAG GAAGACTGAC AGTGCGGACC GAGAGGAGTC TCAAAATCCC

3901 AAACAGAGAA ACCCAGGGGC CACAGCCAGG CCCCGGCCAG CCCCATGGAA ACCCGCCTCG

3961 CAGGGCCACT GACCGTCCAA GTAACAGACT TGGCTGTCCC GGAATGCAGG AACCCCAAAC

4021 GGAACCCATA GGTGCCAGGG TAGGTCTTCG GGGAAGGGAC AGAGGATGAT AGGGGCCAGG

4081 AGGGGGCCGG TCCAGGGGCT GTGGGGGCAG AAGTGGCTGG CATCCTGGGA GCATCATCTG

4141 AGTCTTCGTC TAGCCAGTTC ACGACGCTCT CTGGGAGCAG CAGCTCATCC ACTGCTGGGC

4201 ACAGCTCCGA AGACTGTAGG GGGCGAGACC AGAGTCAAGG GTCAGGTCCC TAGGTCCCCA

4261 GCTCCCAACC CCCGCCCTGG ACCTCCCCAC ACCCAGTCCT TACCAGAACA TTGTTTTCAG

4321 GAAGCCTGGA AGATGAGAGC AGAAAGTCAG TGCTGGTTTT GCTTCCTCTG GGCTTCTACT

4381 TGGGGGGGCG GGGGGGGGGG CTGGGGCTGG GGGTTGGGAT TGGGGTGGGT GGGCAGGGGT

4441 CCCAGAGGGC TGGTGGGGAG CCTGGCCTGC CCTCCACTTA CAGGTTCCAC AATTCTGAAA

4501 ATGTCTCCTG GCTCAGAGGG GGGTCGATAT TGAGCTCTGA CTGTGGCTCT TGCATCGCAG

4561 CTCCTGGGAA GGGAGTGTGG GTGCTGTGGA GGGCAAGGCG AGGGGTCAGC GGGAGACAAG

4621 CCCGGGCCCG GGTCCCTCAG CCCCGAGGTG GGAGGGACCC TTCCAGCAAC ATTATACCAG

4681 GGCCTGGGAT GCCTTGGCTC CCCCACCTGG GCCTCCCCAG GAGCAGGGCT GCTCCTGCCC

4741 ATTCCACCCC TGGGCGGCCT ACCCCCACAC ATCCTCAAGG TACAAAGATA ACCAGAAAGG

4801 GGATTCTAGC CCTCTCATTA CAGAGTCCAC AAGCCTTCCT GGTTATAAGG ATGAAGAACG

4861 TCAGCCCACA CCCTCCACCT GGACGAAGAT ACCTGCATGG ACCCCCTCAT TCCCCACCTG

4921 ATCCCCTTCA TCACGAAGCT TCCTAAGGAT CTGCTATCTC CATTCCACCC TTGGCTTGCA

4981 GAGTCTTCAG GACCTCCCGG CCGGCATCCC CGACCTTGTC TTCCAGTATT CTCCAGCCAG

5041 TGGCTGCCCT TCTCTGCTGC CACACACACA CCCCAGTTCA TTGGCTCGGG GTTCCCTCTA

5101 TTGGGAACCC TGGTCCAGCC TCTGCTTGCA GGGGAGTCAC CTTCTAGCAG ATCATCCCGA

5161 ATGTCACAGC TCCTCAGGGA TGTCTGCCCA GACCACCCAG GCAAAGACTG CCAGCCAAAC

5221 GCTGTCACCA GTTTTTTTAA AAAAGATTTG ATTAATTAAT TTATTTGAGA GAGAGAACAA

5281 GAGTGAGAGA GTGGCAGAGG GAGGGGGAGA GGGAGAAGCA GGTGAGCTAC CTGTCCGGCT

5341 CACTGCAGCA CTAGCAAAAT GCCTCTCACC TGGTTGGGCT CACTGTGGTC AGCAAGTGAA

5401 TGGTGCCGAA GCCAGGAGCA CCTTGTGAGG TGTCCCCAGG AGCCAGCAGG TCAGAGGGAA

5461 GGTCCCAGTC TGGGCCCTGG CGCTGTACTA GGCTGCGCTG CCACCATCTC ATGACATCTT

5521 CCCCAACTCC CGAGTTTGTC AAGAGTTTTG CAAAAAAACA CACCCACTGC AAACCAAAGT

5581 CCCTATTCAT TACCTTTTTG TCTTGTAAAG CACTGACAAG CCTACCGGCC CCACCCTCAC

5641 CCCCAGCAGC TCTTCCCATT AGCATCTCCA TGAACATTCA CCTCTACCTG GATTGAAGGC

5701 CTGCTTTCTT ATGTCCTCAG AGTTTGTGAA ATCCTTTCTT GTGGGTTATC TCATTTGTCC

5761 CAGAACCACC CTGGCTGCCC AACAGGTGAG GAAGCTGGTC TCAGAGAGGT GGAGGGACTC

5821 GCCCACCGCA CACAGGCAAA TGGATGAGCT TTTATTCGAA ATGACCACTC GTGGGGTGCT

5881 TGGGTTGCTC AGTGGTGGAG CACCTGCCTT TGGCTCAGGT TGTGAGCCAA AGGGTCCTGG

5941 GATCGAGTCC CGCACTGGGG TCCCTGTAGG GAGACTGCTT TTCCCTCTGC CCGTGACTCT

6001 GCCGCTCTGT GTCTCTCATG AATAAATAAA ATTTTAAAAA AAGCTTTTGA GATTTTATGG

6061 AAGGGAGCTC AGAAGTCAAA GCCCTAGGTC TGACATCGTA TCATTACTTA TATTTGCTGT

6121 GCAACTCTAC CCATCCCAAG GGCTAGCCTT TTGGCTTCCT TACATGCAGG AATTGAGTTT

6181 TGCTCATCTT TGTTTAGTGA AAAATACCTA GGATGGGGAG ACATGAGCAG CTGTAGCAGG

6241 GCATGGGCTC TGAGGCAGAC AGGCTAGGCC TAAATCCCAT GCCCACCACC TAATAGGTGG

6301 GGAGGTTACT TAACCTCTGT GCCTGGCACA GTAAGAGCTC AACAGATGTG TTTGTTTATA

6361 GGATTTTCCA TTCTATTAGT TTGATTATTA TTTTAGACCC AGAACTTTGT AATTGAGTGG

6421 GTGGGGCTTC TGTAGTCCTG CCTCATTAAT GCTAGTGCCC CTCTTTCAGG GATCTCTGTT

6481 CAGTAAATAT GCCAGGAGGG GCTGTCCTGG GATGCTAGGC CCGAGGTCCC AATCCCACTG

6541 GGAGGGAGAC AGCCTCATAG ACAAAGGAGG TCTCTCCCTC TCTTGGTCCT TGGCAGCAAG

6601 TAAATTCTGT TTTTCTCAAA AAAGCAGGCA CTATCACCAG GAAACCTTTT TGGAGCATGT

6661 ACTTCCCATG GATGAGGATC AAGGGCCATT AAACTAAATT GTCAGAAGCT CAACCAGACC

6721 TTCCAAATGG CTGGGGAGGC AGGACTCATA GATATCCCTG GTGGGAAGGC CAAACTCAGC

6781 TGGGACCTCA TCCAGTTGCC CACCCCACCA CCATCCTAAC AATGGCTGTT GTTTGTAATC

6841 CGCACAGGGG CTCACTGCTC CGGTGGCTTA TCTCATTAAT CCTCTCAAGA AACCATAGCA

6901 GTGGGTATCT TCAAGCTGAA GGAACTGAGG TTGAAGGAAT TAACTCACTC ATTCAGATTC

6961 AGAAAACTCC CGAGCCAGGG ATTTGAATCT GGGCTGTTCA AAAGCCACTG TCCAAGCTCT

7021 AAATAATTAT GCTCCTCTGG CTTCATCCAG AGTCTGTAAA AATAATAATA ATAATAATAT

7081 TTGTTTGGTG CAAAAAGGTT TTTTTGGCTG AAAGCTATTT GGGAAAACAC GATGATTCTT

7141 AAAAGCAGCA AGGCAGTATC TGGTAAGACT CAACAACTTA CTACTTTGCA GAATCACTTC

7201 TAGGTAAGAC TCCCTTAGAA GAGTTTTGTA TGGAGGGAGC AGGAGTTCTG CGCAAATACG

7261 TTCATATCAG TGATGTTCTC CGTGACAGGA AACATCACTA TCCATCCGTA GGGATTGGCT

7321 ACATGAAATA CAAGAGTTTA CTGCAGCAGT TTAAGGAAAT GAATGTATCT GTGTGTACGT

7381 AACTAGATAG ATATAGCTCA GATTGATTAA AAAAATACAA TTCGAGGGGG AAAAAAAAGA

7441 AAAACATGGT AACCTAAGTA GTTTATGTGA ATTAAAAATA TAAAATGAGT ACAGTTGACC

7501 CTTGAATAAT GTGGGGGTTG GGGGCATCGA ACCCTGTGCT ATAGAAAATT CATGAGTTGG

7561 GGCACCTGAG TGGCTCAGTG GCTGAGCTTC TGTCTTGGGC TCAGGTCGTG ATCCTGGGGT

7621 CCTGGAATCG AGTCCCGCAT CAGGATCCCC ACAGGGAGAC TGCTTCTCCC TCTGCCTATG

7681 TCTCTGCCTC TCATGAATAA ATACATTTAA AAAGTCTTAA AAAAAAGGGA TCCCTGGGTG

7741 GCGCAGCGGT TTGGTGCCTG CCTTTGGCCC AGGGCGCGAT CCTGGAGACC CGGGATCAAA

7801 TCCCACGTCG GGCTCCCTGC ATAGAGCCTG CTTCTCCCTC TGCCTATGTC TCTCTCTCTC

7861 TCTCTCTCTC TCTCTCTCTC TCTCTGTGAC TACCATAAAT AAATAAAAAT TAAGAAAAAA

7921 AAAAGTCTTA AAAAAAAAAA ATCCAGGGAC GCCTGGGTGG CTCAGTGGTT GAGTGTCTGC

7981 CTTTGGCCCA GGGCATGATC CTGGGGTGCC CAGATGGAGT CCCGCATGGA GCCTGCTTCT

8041 CTTTACCTGT GTCTCTGCCT CTCTCTCTGT GTCTCTCATG AATAAATAAA TAAAATCTTA

8101 AAAAAAAAAA AAATCCAGGT GTTATCTCTT GACTGACCCA AAATTTAACA GCTTACTATT

8161 GAGAAGCCTT ACCGATAACA TAAGTAACTA AAACATATTA TATATATATA TTATATACTA

8221 CATTCTTTTT TTAAAAAAAG ATTTTATTTA TTTGAGAGGG AGAGAAGCAG ACTACCCGCT

8281 GAGCAGAGAG CTTGATGCAG GGCTGGATCC CAGGACCCTA AGATAGTGAC CTGAGCTAAA

8341 GGCAGACTGG CCACCTAGGT GCCCCCCATA TACTATATTC TTAAAGTAAG CTAGAGATAA

8401 TGCTATTAAT ATAATCACAA GGAAAAAAAG AAACATTCAC AATACTGTTA CTGTATTTAT

8461 CCAAAAAACC CATGTATAAG TGGTCCTACA CAGTTCAATC CTGTGTTTTC TAAGGGTCAA

8521 CTGAATTTAT TTCTTACCAA CATATATATG TGAAAGTATG AAAGACAGAT AAAAAGAATT

8581 AAAACTTACA CTCATGAGAC TGGGGGTACC ATCAAAGCGA CCTTATTTGA TTAATGAAGA

8641 AAAATTTAAA AAGATGGTAT TCATACATTA TTTGTTTATT ATAATATGAA CATGACAAAA

8701 TGTCAAGTGT TATAATTTCC TCTAGCTTCA AGAATAATCA ACATTCTGCC TGTATTGTCT

8761 CCCCTATACT TCTAGTGGCT CCGCAGAAGC CCACAGATAT TTTTGAGTTA CAATCTATGT

8821 AAGTTGAAAT GCACAATCTT AATTTTGCCA TTTAGATAAC AAAGTTTGAC ACATTTTGAT

8881 ATAACCTTGT CACCCAGCCC TACCAAGCAT AAGACACTGC TGCCATGTTT CCTTCTGAAA

8941 GTTAATATAT TTTATGCTTA GATCTACTAT CCATCTCAAA TTGTGTATAG TATGAGGTAG

9001 GGGTCAGAAT CCACTCAGCT CTGCTTGTTT CAGCACTCTC TTCAGACTTT CCTTCCCCCA

9061 TGGATTCATT TTGGAGCTTT TGTAGAAAAC CATTTGATTA CACTTATGTG TCTTTTCCTG

9121 GGCTCTATTC AGTTCATGGT TGTTGTCTTA TGGTAGCTTT ACAGAATTCT TGAAGTCAGC

9181 TAGTTTAAGA CCTCCAGCTT TGTACTACAC TTTCATTTTG GAATTGCTTG TCAGTTTCTA

9241 CAAACAAAAC CAGCTAGGAT TTTGATGGGG ATCAAATGAA ATCATCTTGG AGTACTGACA

9301 TCTCAACACT ATTGAGTGTT CCATCTATGA ATATGGTACT GTTACTTAAA TCTTCCGGGC

9361 AGCCCGGGTG GCTCAGCGGT TTGGCACTGC CTTCGGCCTG GGGTGTGGTT CTGGGGACCA

9421 GGGATCGAGT CCCACGCAGG CTCCCTGCGT GGGGCCTGCT TCTTCCTCTG CCTGTGTCTC

9481 TGCCTCTCAC TTTGTGTCTC TCATTAATAA ACAAATAAAA TAAAATAAAA TAAAAATCTT

9541 CCTTAAATTC GCTCAGCAAT ATTTTGCAGT TTCTAACATA GAACTCTTGT CCGTCTTTCT

9601 TCAATTTATC CCTGGGTATG CCATGTTTTT GGATGCTTTG TAAATGCCAC TGCTTTTATA

9661 ATTCATTTTC CGACTTCAGA TTCAATGGTT GTTATTACAT AAAAATACAA TTTATGTATA

9721 TTGCCTTTGT AACCTGTGAC CTTGCTATAA TCCTTTATCA GTCATGATAG TTCTTCTTTT

9781 TAAGAGGTTT CATTTTTTAA GTAATCTCTG CATCCAAGGT GGGCCTCAAA TTTACAACTC

9841 TGAGATCAAG AGTCATGTAT TCTACTGACT GAGCCAACCA GGTGCCCCTT GTGAGTTTTT

9901 CAATGCTTCT TCTATTAGCC TGGTTAACAC CTTCAGTATA ATGTTGAAGG GGTGAGAATC

9961 AAATCCTTTC CTAGTTCCTC ATCTTAGAAA ACAAGCATTC AGTCTGTCAC TGTTACGTAT

10021 TGATGGTAAC TTGATTGGGG GTGCTCTTTA TCAGACTAAG TTTTCCCTTT CCTAGTTTGC

10081 TGGGAGGTTG GCTTTTCTTT TCTTTCTTTT TTTAAAATCA TGAATAGGTG TTGCGTGATG

10141 TCAACTGCTT TTTATGTATC TGTTGAAATG ATCATAGGAT TTTTATCCTT CAATCTGTTT

10201 ATGTGGCAAA TTACACTGAT TTTTTGAATG TTAAAACATT GCCGTCTAGA GATAAACTCC

10261 ACTTGATTAG GATGTATTTA TCCGTTTTAT AGATAATCAG ATTTACTTTG CTAAGATTTG

10321 TGAGATTTTT GCATATATAC ATATGTGTGT ATATATACAC ACATATGTAT ATATGCTGGC

10381 AGGAATATTG GTCTAAATGT TTTCTTGTAA CATTCTCGTC TGGTTTTGAT ATGAGGATTC

10441 TCAGGATATC ATGAAATTAG TTGGGAAGAT GTTACCTCTT CTACATTCTG AAAGTTTTTG

10501 TAAGATTGTT ATTGTTTCTT CCTCAAGTGT CTGAAGAGAA GAAGAAATAG AATAGAAAAT

10561 AGCATTAACA CAGTAGACTT AAACCCAAAC CTTACAATAA TTGCATTAAA TTAAATAGAC

10621 CAAGCACTCC AATTAAAAGT GATCCAGTGA GGGGGGAAGG AGGGCTGTTT CAACAAGTGG

10681 CACTAGAAGA ACTGGATATA CATCTAGGAA AACCCCAAAA GAAAACAGAA CCCCTACCTC

10741 ACATCACGAT AAAAATTACT CTTAATTAAT TAGTTAATTA ATTATGGCTC AAAGACCTAA

10801 ATATAAAACC TAAAACCCTA AAGATTCTGG AAGAAAATAT CCTAGAATAT CTTCATGATC

10861 TTGGGGTCAG CAAGGATTTT CTAAACACAC AGAACACATT TGAAAGACAG AAGATAAATC

10921 TGCTGCTGCT CTGCCTTAGA GGCTGACAGA GAGGCAAGAA ACAGGTCAGG AGAAAATAGA

10981 TACTGACAAA AGACTCCTAT CCGGAATATA TAAAGAACTG CATGCAGCTG AACGCAAGTT

11041 AAAATGGCCA ATAAGCACGT GGAAACTGAT CACAGCTAAG AGAATTGCAA CTGAAAACCA

11101 CAATGATGCA CCACATTAGA AAGTCTGACA AAACCAGATG TGAGGAAAGA CAGCTGTTGC

11161 TGGTGGGTAC ATAAAACAGA ACAACCACTT TGAAATATAC TAATTTTAAA AATCAAATTT

11221 AGATCTCTCT CACCTAGCAA ATTCCACTCC CAGACGGTTA TAAAAAACAA AAACAAAACA

11281 AAACAAAACA CACACACACA CAAACACCCA AAACCAACCC CATGAAAACA TAGGACCACA

11341 AAGAGAATTG TACAAGAATG TTAATAGCAG CTTTATTCAA AATAACCCCG AACGGTTAAC

11401 AACCCAAGCG GTCACCGAAA GGAACATGGA TTAAAAAAAA CCTCACGGTT TATTCATCCA

11461 GTGAGATGTG CTAAATACAA AAATACAAAG GTGCAAAAAA CACTGATACA CTACTGATAC

11521 AGTAGTACTG GTTACACAGG AACAAGGATG GGTGTCACAG GTATTATGCT GCCGGCGAGA

11581 AGCCTACACA AAGGAGCACA CACTATGTGG GTCTATTTAC ACGACGCTCT CTTCAACAGG

11641 CACAAGTAGT GACAGAACTC CAAGATGAGG GTCAGAGACC CCCAGAGGGG AGAGGCCCAA

11701 GAACCTTCTC TGGGGCGATG ACAATGTGTA AATTGTGTCA TTTGACAGGC TGCTGGTTAG

11761 CAGCCCAAAC CTGTCTAATT GCAGTGAGTG TAAATGCTAC CTCAATTTCA GAACAAGCCG

11821 CACACAAGCA CCGCGTGCAC TGGCCGGTAC CGAGCAGGTG CACAGGTAGG TGAGCGAGCG

11881 CAGGCTGGAA GCACCGCGCG CCCGGAGTGA CTGACAGGCG GGAGGCGGTA TCCCGTCCCC

11941 TCGGCTCGGC TCGGCTCGGC TCGGCTCGGC GGGCCCCGCC CCCTCAAGCC CCGCCCCTTC

12001 CGCCCTCACG ACGTTCGCTC TGCGTCGGAA CTCGGAATGG CTGAGTCCCG AGCCGGATGC

12061 CAGAGAAACC GTGTCTCGGC CCCACACAAA CGGCGCATCG TCGCATTCTG CCATCGTTGT

12121 CATTCTCAAA TGTACTCGTG CTCGAAAATA CCCGGGACTT GCCGTCGGCA CGCTTCTGGA

12181 GACCTCGTCC CTCGCCCGCC CAGATTGCAG GCGATGGGGT GGGGCGCACC ATTCAAGGAG

12241 TGGGGGAAGG CTCGAGGTTT GCATCCAAAC AGACCCTCCC TCCCGGCCTG CCAAAAGTAG

12301 CAGCTAAGGA GTTCAGGGGA AAAAAAAAAA AAAAAAAAAA AAAAAAGAAA GAAAAGAAAA

12361 AATAAGAAAG AAAAAAGAAA AGAAAAGAAA AGAAAAGAAA GCCAGTAGAG GAGGCGGCGC

12421 GCAGCCTCCC TGAGCACGTC AGTCTAAATG ACACTTTCCC TCTCCTCGGG GGAACAGTGA

12481 ACTGCTCCGG AAAAAAAACC GGAAACGCTG CGGCAAGGGG CAGAAATGTA AATGTTCGAC

12541 CAAACAATAA CAGAGCTCGC GGGCCTCCTG GACCCGGACC GTCCCGCCGG GCGTCGCGGG

12601 CAGACTGCTC GTGGAGCGCT ACCCACGTCC ACGCCAGGCA GTCTACTAGG ATCGTCTCCA

12661 TTTAGTGGAG CCAGGGGCCG CTGGGGAGAT CCCTCCGGGA AGGACAAAGG TCCGGAAGTT

12721 CTGGGACCTG AGCGGCTTGG GCTTCCTGGA TCGACCCCCC CGAATGTGAT CATGTCGGGA

12781 CGGAGCCCGA GAGGACCCTC CAGGGATGCT GCGGGCTCGA AGATCTCGGG GGTCCCCAAA

12841 ATCCCGAGAT CGGGGAAAAA ACAATCTCGG ATCCTGATGC TTTGTGCGTC TTATTTCGGG

12901 GTTCTTCACA CCCGGCACGG GGAGAGAAAG GGAGGGGAAG GGTGAGGAGG AACCCCTTCG

12961 GCCGGCTGCA CGTCCCCCTC CTATAAAGCT GAGTCCGGCT GCTCCCAACA GCCCCTTCAT

13021 CTGCACCCAG GCCTCCCAGC GGTGCATTCC GATATTGGTG CCGCCAGCCA AACTTCCATT

13081 CCCCCGCGAT CGCTGATCGC GTGCCCCACC CCCAGACCCC AATTCTCCCC AAGCTCAAAA

13141 CACACAGAGC CGAGAGCCGG TGACTCAGAC AGGACTCATC TGGCTCAGTC AGGAGCTTAC

13201 CCGAACCTCA GGGGAGCGTG TCACCGTCCT GGGAAGCACG CTCCGAGGCC GGACGCCAGA

13261 GGATCCCGGA GCCCGGCGGC TACCTGCGCC CAGGATGGTG GCTCTGGACT TTTGAGAAGC

13321 TCAAAACTTT TAGCGCCAGT GAGTCCGAGC ACATGGGAGG GG

//
